# Adverse events associated with the delivery of telerehabilitation across rehabilitation populations: A scoping review

**DOI:** 10.1371/journal.pone.0313440

**Published:** 2024-11-19

**Authors:** Thomas Yau, Josh Chan, McKyla McIntyre, Damanveer Bhogal, Angie Andreoli, Carl Froilan D. Leochico, Mark Bayley, Ailene Kua, Meiqi Guo, Sarah Munce

**Affiliations:** 1 Temerty Faculty of Medicine, University of Toronto, Toronto, Ontario, Canada; 2 Western University, London, Ontario, Canada; 3 Toronto Rehabilitation Institute-University Health Network, Toronto, Ontario, Canada; 4 Division of Physical Medicine and Rehabilitation, Department of Medicine, Faculty of Medicine, University of Toronto, Toronto, Ontario, Canada; 5 Department of Physical Medicine and Rehabilitation, St. Luke’s Medical Center, Global City and Quezon City, Philippines; 6 Department of Rehabilitation Medicine, Philippine General Hospital, University of the Philippines Manila, Manila, Philippines; 7 KITE Research Institute, Toronto Rehabilitation Institute-University Health Network, Toronto, Ontario, Canada; 8 Department of Occupational Science and Occupational Therapy, University of Toronto Rehabilitation Sciences Institute, University of Toronto, Toronto, Ontario, Canada; 9 Institute of Health Policy, Management and Evaluation, University of Toronto, Toronto, Ontario, Canada; 10 Rehabilitation Sciences Institute, University of Toronto, Toronto, Ontario, Canada; 11 Bloorview Research Institute, Holland Bloorview Kids Rehabilitation Hospital, Toronto, Ontario, Canada; Wroclaw University of Science and Technology, POLAND

## Abstract

**Objective:**

This scoping review aimed to map existing research on adverse events encountered during telerehabilitation delivery, across rehabilitation populations. This includes identifying characteristics of adverse events (frequency/physical/non-physical, relatedness, severity) and examining adverse events by different modes of telerehabilitation delivery and disease states.

**Introduction:**

Telerehabilitation, a subset of telemedicine, has gained traction during the COVID-19 pandemic for remote service delivery. However, no prior scoping review, systematic review, or meta-analysis has identified and summarized the current primary research on adverse events in telerehabilitation. Understanding adverse events, such as falls during physiotherapy or aspiration pneumonia during speech therapy, is crucial for identifying limitations and optimizing delivery through risk mitigation and quality indicators. This understanding could also help to improve the uptake of telerehabilitation among clinicians and patients. This review addresses this gap by summarizing published literature on adverse events during telerehabilitation.

**Methods:**

The review followed the Joanna Briggs Institute framework and adhered to the Preferred Reporting Items for Systematic Reviews and Meta-Analyses Extension for Scoping Reviews guidelines. The review protocol was registered and published on Open Science Framework. A comprehensive search across multiple databases (MEDLINE ALL/EMBASE/APA PsycINFO/CENTRAL/CINAHL) was conducted. Screening, extraction, and synthesis were performed in duplicate and independently. Data extraction followed the Template for Intervention Description and Replication framework and also involved extraction on authors, publication year (pre- or post-COVID), population, sample size, and modes of telerehabilitation delivery (asynchronous, synchronous, hybrid). For synthesis, data were summarized quantitatively using numerical counts and qualitatively via content analysis. The data were grouped by intervention type and by type of adverse event.

**Inclusion criteria:**

This scoping review included qualitative and quantitative studies published between 2013–2023, written in English, and conducted in any geographic area. All modes of telerehabilitation delivery were included. Systematic reviews, meta-analyses, commentaries, protocols, opinion pieces, conference abstracts, and case series with fewer than five participants were excluded.

**Results:**

The search identified 11,863 references, and 81 studies were included in this review with a total of 3,057 participants (mean age:59.3 years; females:44.6%). Modes of telerehabilitation delivery (whether asynchronous, synchronous or hybrid) used in the studies included videoconferencing (52), phone calls (25), text messaging (4), email (6), mobile apps (10), and internet-based virtual reality systems (3). A total of 295 adverse events occurred during 84,534 sessions (0.3%), with the majority being physical (e.g., falls or musculoskeletal pain), non-serious/non-severe/mild, and unrelated to (i.e., not caused by) to the telerehabilitation provided.

**Conclusions:**

From the 81 included studies, telerehabilitation was delivered with related adverse events being rare, and mostly characterized as mild/non-severe. A comparable occurrence of adverse events (~30%) was found between asynchronous and synchronous telerehabilitation studies. When categorized by disease type, cardiac telerehabilitation studies had the most frequent adverse events. Detailed reporting of telerehabilitation interventions and adverse event characteristics is recommended for future studies (i.e., use of TIDieR reporting guidelines). Telerehabilitation has the potential to make rehabilitation services more accessible to patients; however, more evidence on the safety of telerehabilitation is needed.

## Introduction

Telerehabilitation is a subset of telemedicine connecting rehabilitation providers and patients at a distance [1]. Telerehabilitation can also provide services to those who would not normally be able to access traditional rehabilitation, such as those living in remote communities or patients with disabilities which hinder participation in in-person sessions [2], assuming that appropriate internet connections are available. The convenience of telerehabilitation may lead to decreased travel expenses for participants and higher attendance rates for individuals with other life commitments [3]. Multiple systematic reviews have shown the effectiveness of telerehabilitation; for instance, a study by Dias et al. found high-quality evidence that telerehabilitation was not different from other interventions for adults with physical disabilities in terms of long-term improvements in pain, physical function, and quality of life [4–8]. However, there remain questions about potential limitations of telerehabilitation, in particular its safety compared to in-person rehabilitation [9]. Due to the remote nature of telerehabilitation, patients cannot receive immediate physical assistance from rehabilitation providers if they experience an adverse event, which are defined as “negative consequences of care that result in unintended injury or illness which may or may not have been preventable” [10, 11]. For instance, they may include falls during physiotherapy or aspiration pneumonia due to speech language pathology swallowing assessments [12, 13]. There is a paucity of research surrounding the patient safety of telerehabilitation, potentially contributing to its limited uptake among clinicians and patients [14]. The rationale for the current review is as follows: while many individual studies include safety data, there exists a research gap as there has yet to be any synthesis of the existing literature that summarizes the currently available research on adverse events related to telerehabilitation. There has been a prior scoping review on measures to ensure safety during telerehabilitation for patients with stroke, specifically, but the current review differs as it focuses on adverse events and encompasses all health/chronic conditions that could be served by telerehabilitation [15]. This scoping review aimed to conduct a systematic search of published literature on adverse events during the delivery of telerehabilitation, across rehabilitation populations, and map out the extent of existing research. This included identifying characteristics of adverse events (frequency, physical versus non-physical, relatedness to telerehabilitation, severity) and examining adverse events for different modes of telerehabilitation delivery and disease states. The World Health Organization (WHO) recognizes patient safety as a global health priority, and notes that investing in patient safety is important for health outcomes, cost reduction related to patient harm, and health system efficiency [16]. It is important to understand adverse events associated with telerehabilitation delivery, so that safety precautions and risk-mitigation measures can be thoughtfully planned and implemented, to optimize telerehabilitation’s uptake and delivery. Knowledge of the safety of telerehabilitation can help patients make more informed decisions, aid in clinical and funder decision-making and inform safety quality indicators for telerehabilitation.

## Methods

This review adhered to the Joanna Briggs Institute (JBI) methodological framework for scoping reviews, which provides guidance on the outline of the review, inclusion criteria (i.e. Population (or participants)/Concept/Context), search strategy, extraction, presenting and summarizing the results, and any potential implications of the findings for research and practice [17]. The reporting of the scoping review adhered to the Preferred Reporting Items for Systematic Reviews and Meta-Analyses Extension for Scoping Reviews (PRISMA-ScR) guidelines, to ensure all the components of a high-quality scoping review were completed, and a filled checklist is viewable in the S1 Appendix [18]. Our team included members with extensive experience in scoping reviews and telerehabilitation.

### Protocol and registration

The protocol was registered and published on Open Science Framework on June 26, 2023 (Registration DOI: https://doi.org/10.17605/OSF.IO/C3ZHQ).

### Eligibility criteria

Various study designs were considered in this scoping review (e.g., experimental, quasi-experimental (quasi), observational, qualitative, mixed, and multiple methods). However, systematic reviews, meta-analyses, commentaries, protocols, opinion pieces (editorials), abstracts from conferences, and case series of <5 participants were not included. Studies were limited to those published between 2013–2023, because a study by Zheng et al. found that 2013 was the start of a more significant development period of telerehabilitation, with only a few papers on telerehabilitation published prior [19]. Additionally, the year 2013 marked the emergence of video communication technologies such as Zoom or Google Hangout that are commonly used in telerehabilitation today, which ensures that the review’s results are relevant to the current practice of telerehabilitation [20]. Studies had to be written in the English language but could be from any geographic area. All modes of delivery for telerehabilitation (asynchronous, synchronous, or hybrid) were eligible.

### Search strategy

Search strategies were developed by a librarian with experience searching the health sciences literature and conducting systematic and scoping reviews. The following databases were searched on the Ovid platform: MEDLINE ALL, EMBASE, APA PsycINFO, and Cochrane Central Register of Controlled Trials (CENTRAL). The Cumulative Index to Nursing and Allied Health Literature (CINAHL) database was searched on the EBSCOhost platform. An initial strategy was created in MEDLINE ALL and sent to the team for review. Once the test strategy for MEDLINE ALL was agreed upon, the librarian sought out a volunteer librarian to provide a Peer Review of Electronic Search Strategies (PRESS) review.

The MEDLINE ALL search strategies were translated using the command language, controlled vocabulary, and appropriate search fields for each database and search platform. Search terms included Medical Subject Headings (MeSH), EMTREE terms, American Psychological Association thesaurus terms, and CINAHL headings and text words to capture concepts and synonyms of telerehabilitation and adverse events. Results were limited to the English language and the publication period from 2013 to present. The full MEDLINE ALL search strategy can be viewed in S2 Appendix.

### Study/Source of evidence selection

All identified citations were imported into EndNote, a reference management tool, to remove duplicates. They were then transferred into Covidence (https://www.covidence.org/), a web-based reference manager software. All rounds of screening were completed in duplicate and independently. After completing a pilot test, titles and abstracts were screened. Sources that met the inclusion criteria were retrieved in full. A list of excluded studies can be seen in S3 Appendix. This was then followed by a round of screening based on full texts.

### Data extraction

Reviewers TY, JC, DB, AA, CL, and MG independently piloted the extraction form with a random sample of studies and made necessary revisions. Each study was abstracted in duplicate by two different reviewers. TY completed the extraction of all studies, and JC, DB, AA, CL, and MG completed the extraction in duplicate. Extractor details can be viewed in S4 Appendix. The results from the two reviewers were compared, and conflicts were resolved by discussions between TY and MG. The data extracted followed the Template for Intervention Description and Replication (TIDieR) framework, including name of intervention, objective/rationale, materials used, procedures, provider, specific mode/s of telerehabilitation delivery (full telerehab vs in-person hybrid, synchronous: videocall, phone call, instant messaging, web-based such as using either virtual reality or augmented reality; asynchronous: text/ audio/ video messaging, e-mails, on-demand resources; or hybrid: combination of any synchronous and asynchronous methods), location of therapist and patient, period of time, number of sessions, schedule, duration, intensity/dose, tailoring, modification over the course of study, and adherence [21]. It also included specific details including authors, year of publication (before or after the COVID-19 pandemic with pre-COVID defined as data collection starting before March 11, 2020 according to the World Health Organization) [22], population and sample size (including number of patients in each arm and total sample size), age and sex of participants, population type, study design, materials used, and outcome measures (adverse events including type, expected versus unexpected, related versus unrelated, and description). The extraction form can be viewed in S5 Appendix. Quality/risk of bias assessment was not completed as this was not the purpose of a scoping review. All possible data that could be extracted from the included studies were extracted; there were no missing data.

### Data analysis and presentation

Data from this scoping review were summarized quantitatively using numerical counts and qualitatively via content analysis, based on best practices for reporting of scoping reviews [23]. The data were grouped by intervention type and by adverse event type (physical, social, psychological) and analyzed/coded manually. Numerical counts and content analysis were used to reveal trends in the data such as the most common method of telerehabilitation, the health condition with the most adverse events, and the frequency of different types of adverse events. Synthesis occurred in duplicate and independently.

## Results

### Study/Source of evidence selection

A total of 11,863 references were identified from the initial search. After removing 3,506 duplicates via EndNote, a total of 8,357 references remained. Next, 833 other duplicates were identified and removed after importing all records into Covidence, leaving a total of 7,524 studies for title and abstract screening. Reviewers TY, JC, and DB independently piloted a random subsample of titles and abstracts to identify any revisions of the inclusion/exclusion criteria. After revision, title and abstract screening commenced and 7,346 studies were found to be irrelevant, leaving 178 studies for full-text screening. Full-text screening identified 97 studies for exclusion, leaving 81 studies remaining for extraction. The results of the search and study inclusion process are illustrated in a PRISMA-ScR flow diagram (see S6 Appendix).

### Study characteristics

**Fig 1:**
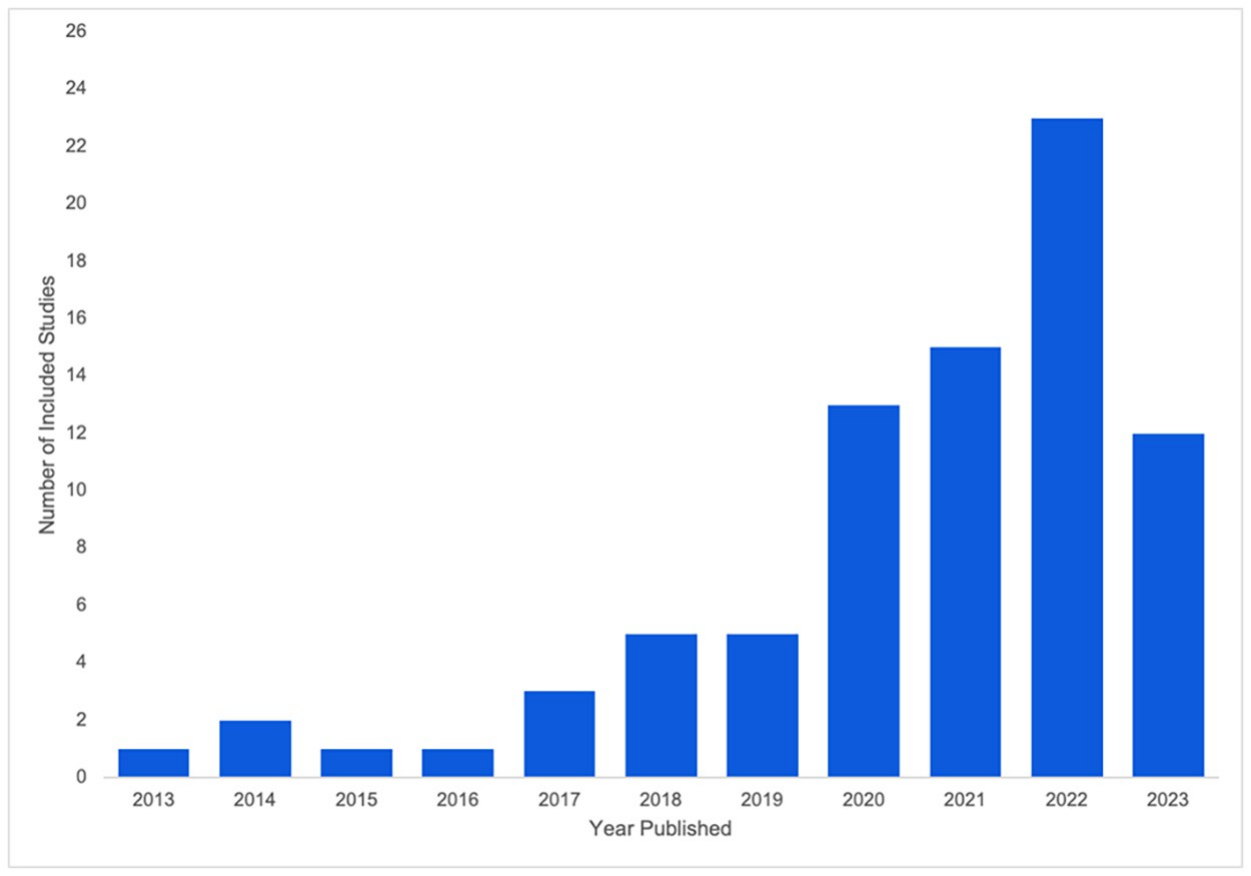
Included studies by publication year.

The total number of included studies was 81, of which 43 (53.1%) were published post-COVID. The distribution of studies by publication year is shown in Fig 1, demonstrating the increase in articles on telerehabilitation-related adverse events from 2020 onwards.

### Population and sample size

From the 81 studies, there were a total of 3,057 participants in the intervention groups, with the mean, median, minimum, and maximum number of participants being 37.7, 17, 4, and 425, respectively. There were a total of 38 studies that had control groups, with a total of 1,486 participants. The mean, median, minimum, and maximum number of controls were 39.1, 24, 5, and 425, respectively. Thus, for the studies that did have controls, the number of participants in the intervention and control groups seemed relatively even.

**Fig 2:**
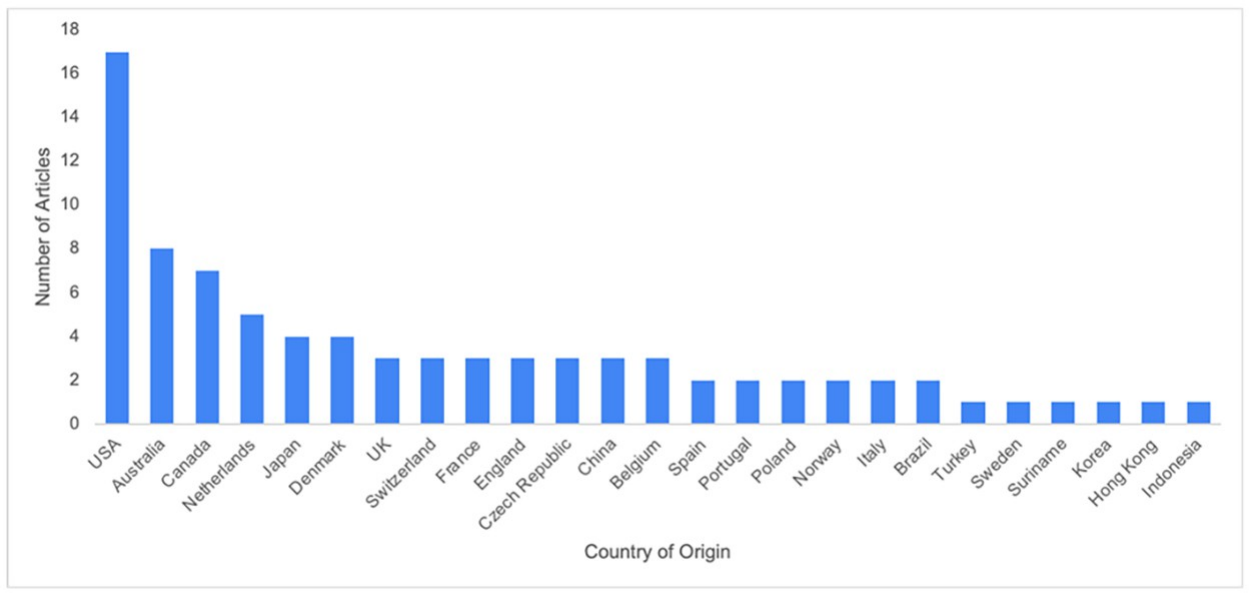
Included studies by country of origin.

The mean age was 59.3 years, not including a study involving primary and secondary school children as mean age was not reported. Almost half (44.5%) of the participants in the included studies were female. The number of articles from each country of origin can be seen in Fig 2.

**Fig 3:**
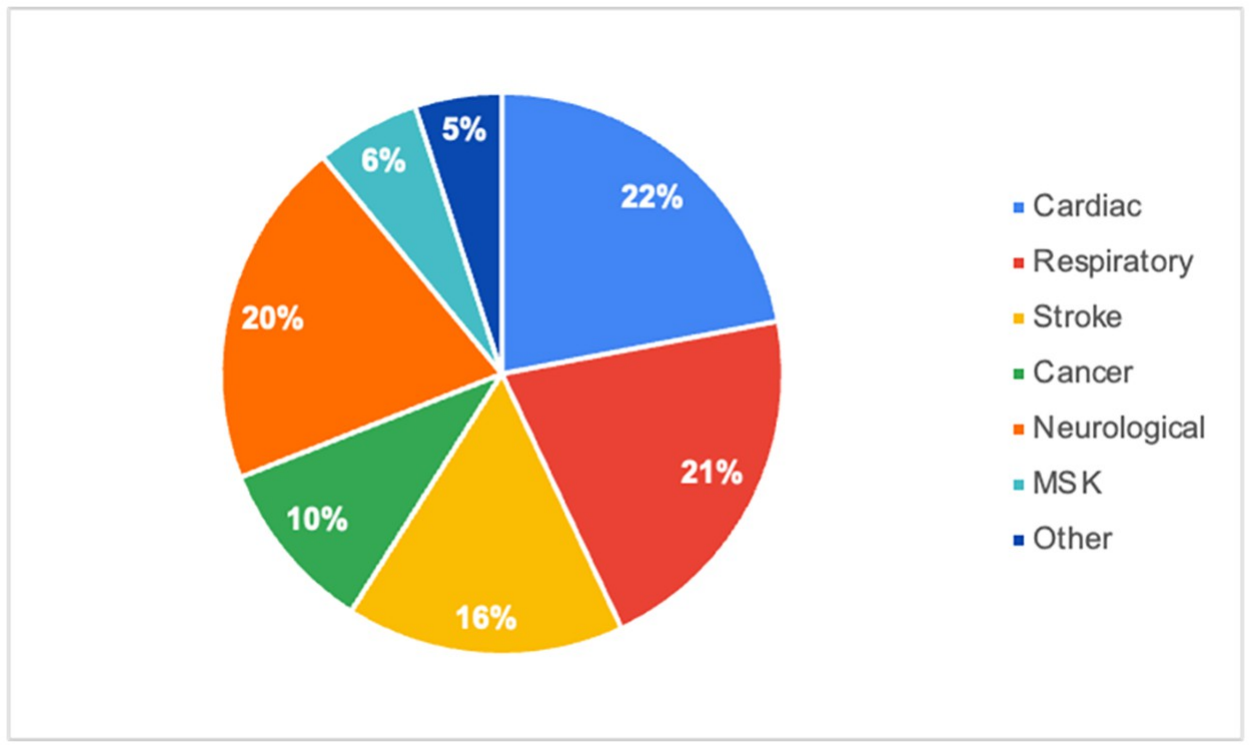
Included studies by disease type. MSK = musculoskeletal.

The included studies comprised of participants with various disease conditions. A full breakdown can be seen below and visualized in Fig 3.

Cardiac (18 studies, 22.2%)—cardiac/cardiovascular disease, 6 [24–29]; heart failure, 5 [30–34]; coronary artery/heart disease, 3 [35–37]; transcatheter aortic valve implantation (TAVI), 2 [38, 39]; advanced combined cardiopulmonary disease, 1 [40]; pediatric heart disease, 1 [41].

Respiratory (17 studies, 20.9%)—COVID-19, 8 [42–49]; COPD, 3 [50–52]; lung transplant, 3 [53–55]; chronic respiratory disease, 1 [56]; lung cancer, 1 [57]; severe cystic fibrosis, 1 [58].

Stroke (13 studies, 16.0%)—stroke, 10 [59–68]; upper extremity dysfunction/paresis from stroke, 2 [69, 70]; post-stroke aphasia, 1 [71].

Cancer (8 studies, 9.9%)—esophageal/esophagogastric cancer, 3 [72–74], unspecified cancer, 2 [75, 76]; glioma, 1 [77]; hematological cancer, 1 [78]; pediatric cancer, 1 [79].

Neurological (16 studies, 19.8%)—Parkinson’s disease, 8 [80–87]; cerebral palsy, 2 [88, 89]; multiple sclerosis, 2 [90, 91]; dementia, 1 [92]; mild cognitive impairment, 1 [93]; mild traumatic brain injury, 1 [94]; unspecified neurologic diseases, 1 [95].

Musculoskeletal (5 studies, 6.2%)—anterior cervical discectomy and fusion, 1 [96]; burn injury, 1 [97], chronic musculoskeletal pain, 1 [98]; inflammatory myopathies, 1 [99], total knee replacement, 1 [100].

Others (4 studies, 4.9%)—geriatric, 1 [101]; overweight and obese, 1 [102]; sarcoidosis, 1 [103]; HIV, 1 [104].

### Study design

From the 81 included studies, a total of 84,534 telerehabilitation sessions were conducted. For study design, 36 studies were quasi-experimental [29, 31, 33, 35, 37, 39–44, 48, 50–52, 57, 58, 61, 63–67, 69, 72, 78–80, 82, 83, 85, 87, 92, 99–101], 12 were mixed methods [38, 54, 55, 62, 73, 76, 81, 84, 89, 93, 95, 96], 2 were qualitative [59, 68], and 3 were observational [47, 49, 75]. There were 28 randomized controlled trials included [24–28, 30, 32, 34, 36, 45, 46, 53, 56, 60, 70, 71, 74, 77, 86, 88, 90, 91, 94, 97, 98, 102–104].

### Intervention characteristics

There were 63 studies that were fully telerehabilitation-based (i.e., patients and therapists in separate physical locations) [25–27, 29–32, 34–37, 40–43, 45–50, 52–55, 57–59, 61, 63, 64, 66, 68–71, 73, 76–85, 87–98, 100–102, 104], and 18 were hybrid (i.e., with an in-person component) [24, 28, 33, 38, 39, 44, 51, 56, 60, 62, 65, 67, 72, 74, 75, 86, 99, 103]. There were 46 studies that used synchronous (real-time) methods of delivering telerehabilitation [24, 29–32, 34, 35, 38–44, 46–52, 55–57, 62–65, 69, 71, 74–76, 81–85, 87, 89, 92, 94, 95, 97, 101, 102], 30 studies that used asynchronous (store-and-forward) methods [25–28, 33, 36, 37, 53, 54, 58–61, 66, 70, 72, 73, 77, 78, 86, 88, 90, 91, 93, 96, 98–100, 103, 104], and 5 studies that were hybrid [45, 67, 68, 79, 80]. On average, sessions were provided 2.68 times per week, for 10.26 weeks, and for 46.52 minutes per session. Various communication methods were used: videoconferencing (52 studies) [24, 27, 29–32, 34, 35, 38–41, 43–52, 55–57, 62–65, 67–69, 71, 73, 75, 76, 79–85, 87, 89, 92, 94, 95, 97, 101–103], phone calls (25) [25, 26, 28, 33, 36, 37, 53, 59, 60, 70, 72, 73, 75–79, 81, 86, 88, 93, 96, 98, 100, 104], mobile application (10) [34, 42, 53, 54, 59, 66, 74, 76, 99, 103], email (6) [54, 58, 72, 73, 77, 91], Internet-based virtual reality (VR) systems (3) [61, 67, 93], and text messaging (4) [36, 58, 79, 98].

Studies employed different healthcare providers, with the majority being physiotherapists (61 studies), followed by nurses (12), physicians (11), exercise physiologists/sport exercise and rehabilitation researchers/certified sports medicine exercise trainers (6), occupational therapists (4), speech-language pathologists (2), kinesiologists (1) and an adapted physical activity coach (1). One study had an unspecified multidisciplinary team with therapy staff and allied health professionals, while another had an unspecified type of licensed therapist. In some studies, other allied health professionals also provided health counseling/interventions. These included clinical pharmacists in 1 study, respiratory therapists in 1 study, nutritionists in 1 study, and psychologists in 2 studies.

The interventions included different types of therapy and exercises: strength/resistance in 45 studies, aerobic/endurance/cardiovascular in 43 studies, balance in 18 studies, stretching in 9 studies, walking in 7 studies, respiratory function training/breathing exercises in 8 studies, cognitive rehabilitation in 4 studies, speech language therapy/swallowing training in 3 studies, yoga in 2 studies, neurological rehabilitation for upper limb motor recovery in 1 study, and dance in 1 study. There were also a few studies that did not define the telerehabilitation intervention as any of these categories. These included studies involving Virtual Reality (VR) exergames, transcranial direct current stimulation with tracking training therapy, and pediatric rehabilitation (enhancing motor development and environmental enrichment).

While most studies used common hardware like a phone/tablet/laptop/video camera, a few used more technologically advanced tools for telerehabilitation. There were 6 studies that used virtual reality systems. Studies involving VR exergames monitored patients’ movements via gesture tracking using a force plate, inertial motion trackers, infrared sensors, or a Kinect sensor. Certain devices used consoles and a range of controllers for gross and fine movement, as well as tangible user interfaces for goal-based activities. One study employed the use of transcranial direct current stimulation equipment. Another set up external kiosks with computers and webcams in the nearby community due to the low availability of affordable home broadband connections. Commercially available game systems, such as the Xbox Dance Mat or NintendoVR Wii Fit, were part of a few interventions.

### Adverse events

A total of 295 adverse events occurred from the 84,534 telerehabilitation sessions, representing 0.3%. Of the 81 included studies, 59 reported no adverse events.

There were 22 studies (10 quasi, 5 mixed, and 7 RCT) that identified adverse events, involving 1,241 participants over 41,186 sessions.

Of the 81 included studies, 40.0% of total adverse events (n = 118) were obtained from those that employed synchronous telerehabilitation. Among those studies using synchronous telerehabilitation, 28.2% indicated some adverse events. In contrast, 60.0% of total adverse events (n = 177) were obtained from studies that employed asynchronous telerehabilitation. Of those studies using asynchronous telerehabilitation, 30.0% indicated some adverse events. No adverse events were reported from those that employed hybrid telerehabilitation.

The severity of the adverse events was mostly classified as non-serious, mild, or minor by the study authors (Table 1).

**Table 1:** Adverse events by severity.

Severity	Number of Adverse Events
**Non-injury**	1
**Non-serious, non-severe, mild**	129 ^b^
**Minor**	50 ^a^,^b^
**Moderate**	6
**Serious or severe**	20 ^b^
**Severity not defined**	62 ^a^

^a^ = one study with adverse events in this category did not report the total number of adverse events.

^b^ = one study with adverse events in this category only reported the number of participants per severity.

There was a broad range of descriptors used to identify the degree to which the adverse event was related to the telerehabilitation intervention, as opposed to other factors; many of these were not defined by any specific criteria. Only 15 of the 295 total adverse events were characterized by study authors as related, 2 probably related, 8 possibly related, 1 potentially related, 5 unlikely, 147 unrelated, and 94 undefined. There were 2 studies that only reported the number of participants per level of relatedness, rather than the specific number of adverse events. Another study reported a description of the adverse events but without a numerical count or report of relatedness.

Almost all the adverse events were physical. The majority were pain-related (musculoskeletal pain/strain or headache), but also included fatigue, falls, dizziness, and cardiac-related adverse events (e.g., chest discomfort, palpitations, angina, tachypnea, etc.). Only 3 adverse events were non-physical. For instance, one participant felt concerned about the possibility of falling, another participant had an unrelated adverse event of anxiety, and a patient with depression felt demotivated when asked to complete a measure of mood [48, 64, 93].

When categorized by disease type, cardiac rehabilitation studies had the most frequent adverse events, with 92 adverse events across 6 studies, including fatigue, palpitations, angina, diaphoresis, dyspnea, and syncope [24, 26, 28, 29, 31, 32]. Studies on people with Parkinson’s disease had the second most, with 75 adverse events across 3 studies, such as pain, neuropathy, loss of balance, dizziness, falls, and increased awareness of hand tremors [82, 83, 86]. Studies on multiple sclerosis were third, with 60 adverse events from 1 study, including falls and skin reactions [91].

## Discussion

There has yet to be any synthesis of the existing literature that summarizes the currently available research on adverse events related to telerehabilitation. Thus, this scoping review aimed to conduct a systematic search of published literature on adverse events during the delivery of telerehabilitation, across rehabilitation populations, and map out the extent of existing research. This included identifying characteristics of adverse events (frequency, physical versus non-physical, relatedness to telerehabilitation, severity) and examining adverse events for different modes of telerehabilitation delivery and disease states.

From the 81 included studies, 295 adverse events occurred during 84,534 sessions, with the majority being physical (e.g., musculoskeletal pain, falls, dizziness), non-serious/non-severe/mild, and unrelated/not caused by the telerehabilitation provided.

Using March 11, 2020, as a marker of pre- versus post-COVID, 53.0% of studies were classified as post-COVID. This may appear to oppose the expected higher number of post-COVID studies following the increase in telerehabilitation usage post-COVID. However, our range of years (2013–2023) included more pre-COVID years, and the accumulated number of pre-COVID studies was substantial. Examining the articles published by each year, there was an increase in articles which reported on adverse events during telerehabilitation delivery from the year 2020 to the present day, indicating the rise in popularity of telerehabilitation following the COVID-19 pandemic. This is in alignment with other studies reporting a general increase in telehealth usage during/following the COVID-19 pandemic [105, 106].

The mean age was 59.3, and almost half (44.5%) of the participants were female. This was a notable finding as there was no age limit in the exclusion criteria of this scoping review. There were only four included studies that involved pediatric populations [41, 79, 88, 89]. It is possible that the limited number of pediatric studies is due to younger children being less likely to participate in telerehabilitation due to the need for a caregiver to be available to accompany them for sessions, or due to their limited ability to remain stationary for the video-to-video format of telerehabilitation. However, it is noteworthy that none of the four pediatric studies identified any adverse events during the delivery of telerehabilitation. Furthermore, of the 81 included studies, only 12 studies (14.8%) reported race. Analyzing adverse events through an equity lens can help to prevent bias and inequities, and lead to corrections of root causes [107].

From the 81 included studies, there were 28 randomized controlled trials. There were a small number of studies characterized as mixed methods (12 studies) and qualitative (2 studies). A greater number of qualitative studies would increase the understanding of patient and clinician lived experiences and perspectives on telerehabilitation and adverse events.

There were a range of different interventions, using different telerehabilitation modalities and providing different types of rehabilitation (mostly aerobic and resistance training). While most studies were entirely telerehabilitation-based, 18 studies included an in-person component. Oftentimes, a few initial sessions were completed in-person so that the therapists could complete an initial physical assessment, or participants could familiarize themselves with the rehabilitation intervention or tools. Videoconferencing and phone calls were the most commonly used methods of telerehabilitation. However, a few studies included more technologically advanced tools, such as virtual reality exergames with gesture tracking or telerehabilitation devices with consoles and controllers. The COVID-19 pandemic accelerated innovations in telerehabilitation, with clinicians and researchers aiming to better understand ways to deliver telerehabilitation in a safe and effective way [108]. The development of new technologies may pave the way to more accessible and safer telerehabilitation, but it is important to test and monitor for safety when implementing new technologies [109]. Lastly, a range of therapists provided telerehabilitation, but physiotherapists were the most common. Multiple physiotherapy organizations across Canada have released guidelines for telerehabilitation delivery, indicating an increased interest in telerehabilitation within the field, which is in alignment with our findings [110, 111].

For the large total number of sessions across the included studies (81 studies), there was a low number of adverse events (295 adverse events/84,534 sessions, 0.35%). While the studies with adverse events represented only 27.0% of the total number of included studies, the number of sessions covered by those studies was substantial. Specifically, nearly half of the total number of sessions from all the included studies were from those studies with adverse events (41,186 sessions/84,534 total sessions). This may indicate that studies that held more sessions (frequency or length of trial) had a higher likelihood of adverse events. However, upon looking at the characteristics of the adverse events, it was found that only 15 adverse events were defined as related to telerehabilitation from all the studies that reported relatedness. In addition, for the adverse events with severity information available, 87.4% were defined as non-serious, mild, or minor.

A slightly larger proportion of asynchronous telerehabilitation studies had adverse events (9/30 studies, 30.0%), in comparison to synchronous telerehabilitation studies (13/46 studies, 28.3%). In addition, the total number of adverse events from asynchronous telerehabilitation studies represented a larger proportion of total adverse events from all included studies. This could be due to other characteristics of the asynchronous telerehabilitation studies, like disease type or the criteria used to define adverse events. However, it is also possible that due to the lack of real-time monitoring, asynchronous telerehabilitation studies allowed for more opportunity for adverse events to occur. More research is needed to compare the safety of the two methods of telerehabilitation, as asynchronous telerehabilitation provides certain benefits over synchronous telerehabilitation. For instance, it does not rely on real-time consultations which require the patient and provider to schedule a time both parties are available, and thus may improve the accessibility of rehabilitation services [112].

Categorized by disease, most adverse events were from cardiac studies, with 92 events across 6 studies, Parkinson’s disease studies with 75 adverse events across 3 studies, and a multiple sclerosis study, with 60 adverse events from 1 study [23, 26, 28, 29, 31, 32, 82, 83, 86, 91]. It is unclear if the adverse events can be attributed to telerehabilitation specifically, or if the nature of these participants’ diseases simply predisposes them to more adverse events whether at rest, during telerehabilitation, or during in-person rehabilitation. For example, of the two cardiac studies that defined what was classified as an adverse event, they included cardiac symptoms such as palpitations and angina, which can occur at rest in cardiac populations [29, 32, 113, 114]. To support the idea that certain diseases cause a predisposition to adverse events, one of these cardiac studies had a non-telerehabilitation control group and reported no significant difference in the number of adverse events between the two groups [32]. In addition, the criteria for what constitutes an adverse event varied from study to study, which could explain the variation in the number of adverse events reported. For instance, one of the Parkinson’s disease studies included baseline pain, ‘not feeling well’, and medication side effects as adverse events, which are common day-to-day events regardless of participation in rehabilitation [82, 115]. Many studies did not specifically define what constituted an adverse event.

Adverse events were mostly physical, with only 3 adverse events being non-physical. However, once again, it was unclear what qualified as an adverse event. One study reported a fear of the possibility of injury or falls. However, the study did not provide any detail as to what specifically about the participant’s fear met the criteria of an adverse event. Nevertheless, this finding points to the need to add safety measures and technical support to ease patients’ fears with technology when implementing telerehabilitation. These findings are consistent with other studies identifying a common lack of familiarity, fear, and frustration with digital technology during telerehabilitation [116].

Many studies were missing descriptive information on the intervention or details about the adverse events. Out of the 81 included studies, the total number of telerehabilitation sessions was not reported in 3 studies, session frequency per week was not reported by 11 studies, length of intervention was not reported by 6 studies, and session length was not reported by 28 studies. There were 7 studies with adverse events that did not report the severity of the adverse events. There was 1 study that just described the types of adverse events but did not report the total number. In addition, of those reporting the severity of adverse events, 2 studies only reported the number of participants that experienced adverse events of each severity category, rather than specific number of adverse events. Detailed reporting is important for the replicability of studies, as well as analysis of the safety of the telerehabilitation interventions [21, 117].

This review has a number of strengths. For instance, all study phases (screening, extraction, synthesis) were completed in duplicate and independently. This review was guided by the JBI methodological framework for scoping reviews, followed recommendations for conducting a high-quality scoping review, and adhered to PRISMA-ScR guidelines [17, 18]. Our team included members with extensive experience in telerehabilitation and conducting scoping reviews.

Limitations of the review include the exclusion of gray literature and studies not published in the English language. No risk of bias assessment, or estimation and comparison of measurement errors were conducted, consistent with the PRISMA-ScR guidelines, as the aim of a scoping review is to map out the extent of literature rather than assess the quality of studies [17, 18]. Indeed, we have previously identified the need for patient training on platforms such as Zoom or Skype, in order to conduct virtual care [108].

This scoping review provides useful insights at an important juncture in time. There has been a surge in the popularity of telerehabilitation due to the COVID-19 pandemic, and now patients, providers, funders, and governments are contemplating how rehabilitation should be delivered in the future. Understanding adverse events related to telerehabilitation will help to identify key limitations for optimizing telerehabilitation delivery, by allowing for the development of necessary risk-mitigation measures and quality indicators. In particular, the finding that most adverse events were from specific patient populations such as Parkinson’s disease, cardiovascular conditions, or multiple sclerosis, may suggest that these groups would benefit from more risk mitigation strategies when participating in telerehabilitation. This could include having a caretaker supervise the telerehabilitation session, providing a telerehabilitation kit [118] explaining important safety measures, or having emergency contacts in case of unexpected severe adverse events. A greater understanding of the safety and optimization of telerehabilitation could help influence government funding and guide policymakers, which is important as a lack of leadership and organizational support has been found to hinder the implementation of telerehabilitation [108]. With the COVID-19 pandemic, many organizations were forced to transition to virtual care delivery, with changes in the rules, regulations, and reimbursement models [119]. Understanding that the current literature shows adverse events are rare during telerehabilitation delivery could reassure policymakers and leaders about using telerehabilitation on an ongoing basis and encourage them to provide funding to grow these programs beyond the COVID-19 pandemic. Similarly, from a patient perspective, these findings of rare adverse events may aid in decision-making regarding participation in telerehabilitation.

Future directions of this scoping review may include a systematic review comparing the effect of telerehabilitation versus in-person care on adverse events, as this scoping review identified 28 randomized controlled trials that met inclusion criteria. Only 7 of the 28 randomized controlled trials identified any adverse events, but a formal systematic review would have to be completed. More studies should examine the role of non-physical adverse events during the delivery of telerehabilitation. Further studies should be conducted in pediatric populations, as there were limited studies identified.

In conclusion, this scoping review found that across the included studies, telerehabilitation was delivered with related adverse events being rare (0.3%), and mostly mild/non-severe. A comparable occurrence of adverse events (~30%) was found between asynchronous and synchronous telerehabilitation studies. When categorized by disease type, cardiac rehabilitation studies had the most frequent number of adverse events. Detailed reporting of interventions and adverse event characteristics is recommended for future studies. Telerehabilitation has grown in popularity and has the potential to make rehabilitation services more accessible to patients; however, more evidence on the safety of telerehabilitation is needed.

## Supporting information

S1 Appendix Preferred reporting items for systematic reviews and meta-analyses extension for scoping reviews (PRISMA-ScR) checklist. (PDF)

S2 Appendix MEDLINE(R) ALL 1946 to June 22, 2023, search strategy. (PDF)

S3 Appendix Excluded studies. (DOCX)

S4 Appendix Data extractors and date of extraction. (DOCX)

S5 Appendix Extraction table of eligible studies. (PDF)

S6 Appendix Prisma-ScR flow diagram. (DOCX)

## References

[1] Leochico CF, Tyagi N. Teleoccupational therapy. Telerehabilitation. 2022;297–308. doi: 10.1016/b978-0-323-82486-6.00020-4

[2] Harkey LC, Jung SM, Newton ER, Patterson A. Patient satisfaction with telehealth in rural settings: A systematic review. International Journal of Telerehabilitation. 2020;12(2):53–64. doi: 10.5195/ijt.2020.6303 33520095 PMC7757651

[3] Chivate S, Sharma M, Shaikh A, Satarkar C. Benefits and challenges of telerehabilitation use by pediatric physiotherapists during the COVID-19 pandemic in Western and Southern India: A Cross Sectional Survey. International Journal of Telerehabilitation. 2022;14(1). doi: 10.5195/ijt.2022.6466 35734383 PMC9186904

[4] Dias JF, Oliveira VC, Borges PR, Dutra FC, Mancini MC, Kirkwood RN, et al. Effectiveness of exercises by telerehabilitation on pain, physical function and quality of life in people with physical disabilities: A systematic review of Randomised Controlled Trials with grade recommendations. British Journal of Sports Medicine. 2020;55(3):155–62. doi: 10.1136/bjsports-2019-101375 33060156

[5] Cavalheiro AH, Silva Cardoso J, Rocha A, Moreira E, Azevedo LF. Effectiveness of tele-rehabilitation programs in HEART FAILURE: A systematic review and meta-analysis. Health Services Insights. 2021;14:117863292110216. doi: 10.1177/11786329211021668 34188484 PMC8212368

[6] Ora J, Prendi E, Attinà ML, Cazzola M, Calzetta L, Rogliani P. Efficacy of respiratory tele-rehabilitation in COPD patients: Systematic Review and meta-analysis. Monaldi Archives for Chest Disease. 2022; doi: 10.4081/monaldi.2022.2105 35086329

[7] Tarihoran DE, Daryanti Saragih I, Saragih IS, Tzeng H. Effects of videoconferencing intervention on stroke survivors: A systematic review and meta‐analysis of Randomised Controlled Studies. Journal of Clinical Nursing. 2023; doi: 10.1111/jocn.16716 37035861

[8] Wu Y-Q, Long Y, Peng W-J, Gong C, Liu Y-Q, Peng X-M, et al. The efficacy and safety of telerehabilitation for fibromyalgia: Systematic Review and meta-analysis of randomized controlled trials. Journal of Medical Internet Research. 2023;25. doi: 10.2196/42090 37097721 PMC10170363

[9] Cottrell MA, Hill AJ, O’Leary SP, Raymer ME, Russell TG. Service provider perceptions of telerehabilitation as an additional service delivery option within an Australian neurosurgical and Orthopaedic Physiotherapy Screening Clinic: A qualitative study. Musculoskeletal Science and Practice. 2017 Dec;32:7–16. doi: 10.1016/j.msksp.2017.07.008 28787636

[10] Lee AC, Deutsch JE, Holdsworth L, Kaplan SL, Kosakowski H, Latz R, et al. Telerehabilitation in physical therapist practice: A clinical practice guideline from the American Physical Therapy Association. Physical Therapy. 2024 May 21;104(5). doi: 10.1093/ptj/pzae045 38513257 PMC11140266

[11] Kizer KW, Stegun MB. Serious Reportable Adverse Events in Health Care. In: Advances in Patient Safety: From Research to Implementation. Rockville, MD: Agency for Healthcare Research and Quality (US); 2005. 21250024

[12] Werden Abrams S, Gandhi P, Namasivayam-MacDonald A. The adverse effects and events of thickened liquid use in adults: A systematic review. American Journal of Speech-Language Pathology. 2023;32(5):2331–50. doi: 10.1044/2023_AJSLP-22-00380 37437527

[13] Caniça V, Bouça‐Machado R, Rosa MM, Ferreira JJ, CNS Physiotherapy Study Group. Adverse events of physiotherapy interventions in Parkinsonian patients. Movement Disorders Clinical Practice. 2022;9(6):744–50. doi: 10.1002/mdc3.13466 35937480 PMC9346232

[14] Leochico CF, Rey‐Matias BM, Rey‐Matias RR. Telerehabilitation perceptions and experiences of physiatrists in a lower‐middle‐income country during the COVID‐19 pandemic. Physical Medicine & Rehabilitation. 2021;14(2):210–6. doi: 10.1002/pmrj.12715 34585855 PMC8661588

[15] Gutierrez-Arias R, González-Mondaca C, Marinkovic-Riffo V, Ortiz-Puebla M, Paillán-Reyes F, Seron P. Measures to ensure safety during telerehabilitation of people with stroke: A scoping review. Journal of Telemedicine and Telecare. 2023; doi: 10.1177/1357633x231181426 37321644

[16] Patient safety [Internet]. World Health Organization; [cited 2023 Nov 8]. https://www.who.int/news-room/fact-sheets/detail/patient-safety#:~:text=Investing%20in%20patient%20safety%20positively,systems%20(4%2C)

[17] Peters MDJ, Marnie C, Tricco AC, Pollock D, Munn Z, Alexander L, et al. Updated methodological guidance for the conduct of scoping reviews. JBI Evidence Synthesis. 2020;18(10):2119–26. doi: 10.11124/JBIES-20-00167 33038124

[18] Tricco AC, Lillie E, Zarin W, O’Brien KK, Colquhoun H, Levac D, et al. Prisma extension for scoping reviews (PRISMA-SCR): Checklist and explanation. Annals of Internal Medicine. 2018;169(7):467–73. doi: 10.7326/M18-0850 30178033

[19] Zheng J, Hou M, Liu L, Wang X. Knowledge structure and emerging trends of Telerehabilitation in recent 20 years: A Bibliometric analysis via CiteSpace. Frontiers in Public Health. 2022;10. doi: 10.3389/fpubh.2022.904855 35795695 PMC9251196

[20] Jalali MS, Landman A, Gordon WJ. Telemedicine, privacy, and information security in the age of covid-19. Journal of the American Medical Informatics Association. 2020;28(3):671–2. doi: 10.1093/jamia/ocaa310 33325533 PMC7798938

[21] Hoffmann TC, Glasziou PP, Boutron I, Milne R, Perera R, Moher D, et al. Better Reporting of interventions: Template for intervention description and replication (Tidier) checklist and guide. BMJ. 2014;348(mar07 3). doi: 10.1136/bmj.g1687 24609605

[22] Infection and Prevention Control Canada. Coronavirus (COVID-19) SARS-CoV-2 [Internet]. IPAC Canada; 2023 [cited 2023 Dec 29]. https://ipac-canada.org/coronavirus-resources

[23] Stemler S. An overview of content analysis [Internet]. 2001 [cited 2023 Nov 24]. https://scholarworks.umass.edu/pare/vol7/iss1/17/

[24] Keteyian SJ, Grimshaw C, Brawner CA, Kerrigan DJ, Reasons L, Berry R, et al. A comparison of exercise intensity in hybrid versus standard phase two cardiac rehabilitation. Journal of Cardiopulmonary Rehabilitation and Prevention. 2021;41(1):19–22. doi: 10.1097/HCR.0000000000000569 33351540 PMC7768817

[25] Batalik L, Dosbaba F, Hartman M, Batalikova K, Spinar J. Benefits and effectiveness of using a wrist heart rate monitor as a telerehabilitation device in cardiac patients: A randomized controlled trial. Medicine. 2020 Mar;99(11). doi: 10.1097/MD.0000000000019556 32176113 PMC7440288

[26] Snoek JA, Prescott EI, van der Velde AE, Eijsvogels TM, Mikkelsen N, Prins LF, et al. Effectiveness of home-based mobile guided cardiac rehabilitation as alternative strategy for nonparticipation in clinic-based cardiac rehabilitation among elderly patients in Europe. JAMA Cardiology. 2021;6(4):463. doi: 10.1001/jamacardio.2020.5218 33112363 PMC7593879

[27] Saitoh M, Takahashi T, Morisawa T, Honzawa A, Yokoyama M, Abulimiti A, et al. Remote cardiac rehabilitation in older cardiac disease: A randomized case series feasibility study. Cardiology Research. 2022;13(1):57–64. doi: 10.14740/cr1346 35211224 PMC8827239

[28] Piotrowicz E, Pencina MJ, Opolski G, Zareba W, Banach M, Kowalik I, et al. Effects of a 9-Week Hybrid Comprehensive Telerehabilitation Program on Long-term Outcomes in Patients With Heart Failure: The Telerehabilitation in Heart Failure Patients (TELEREH-HF) Randomized Clinical Trial. JAMA Cardiology. 2020;5(3):300. doi: 10.1001/jamacardio.2019.5006 31734701 PMC6865325

[29] Pinto R, Lemos Pires M, Borges M, Linan Pinto M, Sousa Guerreiro C, Miguel S, et al. Digital home-based multidisciplinary cardiac rehabilitation: How to counteract physical inactivity during the COVID-19 pandemic. European Journal of Preventive Cardiology. 2021;28(Supplement_1). doi: 10.1016/j.repc.2021.05.013 34840415 PMC8604709

[30] Lundgren KM, Langlo KA, Salvesen Ø, Zanaboni P, Cittanti E, Mo R, et al. Feasibility of telerehabilitation for heart failure patients inaccessible for outpatient rehabilitation. ESC Heart Failure. 2023;10(4):2406–17. doi: 10.1002/ehf2.14405 37221704 PMC10375147

[31] Kikuchi A, Taniguchi T, Nakamoto K, Sera F, Ohtani T, Yamada T, et al. Feasibility of home-based cardiac rehabilitation using an integrated telerehabilitation platform in elderly patients with heart failure: A pilot study. Journal of Cardiology. 2021;78(1):66–71. doi: 10.1016/j.jjcc.2021.01.010 33579602

[32] Hwang R, Bruning J, Morris NR, Mandrusiak A, Russell T. Home-based telerehabilitation is not inferior to a centre-based program in patients with chronic heart failure: A randomised trial. Journal of Physiotherapy. 2017;63(2):101–7. doi: 10.1016/j.jphys.2017.02.017 28336297

[33] Piotrowicz E, Korzeniowska-Kubacka I, Chrapowicka A, Wolszakiewicz J, Dobraszkiewicz-Wasilewska B, Batogowski M, et al. Feasibility of home-based cardiac telerehabilitation: Results of teleintermed study. Cardiology Journal. 2014;21(5):539–46. doi: 10.5603/CJ.a2014.0005 24526507

[34] Peng X, Su Y, Hu Z, Sun X, Li X, Dolansky MA, et al. Home-based telehealth exercise training program in Chinese patients with heart failure. Medicine. 2018;97(35). doi: 10.1097/md.0000000000012069 30170422 PMC6392598

[35] Fanget M, Bayle M, Labeix P, Roche F, Hupin D. Effects of cardiac telerehabilitation during COVID-19 on cardiorespiratory capacities in patients with coronary artery disease. Frontiers in Physiology. 2022;13. doi: 10.3389/fphys.2022.837482 35370786 PMC8969221

[36] Song Y, Ren C, Liu P, Tao L, Zhao W, Gao W. Effect of smartphone-based telemonitored exercise rehabilitation among patients with coronary heart disease. Journal of Cardiovascular Translational Research. 2019;13(4):659–67. doi: 10.1007/s12265-019-09938-6 31820334 PMC7423855

[37] Batalik L, Konecny V, Dosbaba F, Vlazna D, Brat K. Cardiac rehabilitation based on the walking test and telerehabilitation improved cardiorespiratory fitness in people diagnosed with coronary heart disease during the COVID-19 pandemic. International Journal of Environmental Research and Public Health. 2021;18(5):2241. doi: 10.3390/ijerph18052241 33668304 PMC7956401

[38] Brocki BC, Andreasen JJ, Aaroe J, Andreasen J, Thorup CB. Exercise-based real-time telerehabilitation for older adult patients recently discharged after transcatheter aortic valve implantation: Mixed Methods Feasibility Study. JMIR Rehabilitation and Assistive Technologies. 2022;9(2). doi: 10.2196/34819 35471263 PMC9092235

[39] Ashikaga K, Doi S, Yoneyama K, Suzuki N, Kuwata S, Koga M, et al. Efficacy and safety of home-based cardiac telemonitoring rehabilitation in patients after transcatheter aortic valve implantation: Single-center usability and Feasibility Study. JMIR Rehabilitation and Assistive Technologies. 2023 May 17;10. doi: 10.2196/45247 37195764 PMC10233439

[40] Herkert C, Graat-Verboom L, Gilsing-Fernhout J, Schols M, Kemps HM. Home-based exercise program for patients with combined advanced chronic cardiac and Pulmonary Diseases: Exploratory study. JMIR Formative Research. 2021;5(11). doi: 10.2196/28634 34751655 PMC8663616

[41] Khoury M, Phillips DB, Wood PW, Mott WR, Stickland MK, Boulanger P, et al. Cardiac rehabilitation in the paediatric fontan population: Development of a home-based high-intensity interval training programme. Cardiology in the Young. 2020;30(10):1409–16. doi: 10.1017/S1047951120002097 32716280

[42] Kortianou E, Tsimouris D, Mavronasou A, Lekkas S, Kazatzis N, Apostolara Z, et al. Application of a home-based exercise program combined with Tele-rehabilitation in previously hospitalized patients with COVID-19: A feasibility, single-cohort Interventional Study. Pneumon. 2022;35(2):1–10. doi: 10.18332/pne/146521

[43] Howroyd F, Earle N, Weblin J, McWilliams D, Williams J, Storrie C, et al. Virtual Post-Intensive-Care rehabilitation for survivors of covid-19: A service evaluation. Cureus. 2023; doi: 10.7759/cureus.38473 37273405 PMC10236380

[44] Colas C, Bayle M, Labeix P, Botelho-Nevers E, Gagneux-Brunon A, Cazorla C, et al. Management of long covid—the CoviMouv’ pilot study: Importance of adapted physical activity for prolonged symptoms following SARS-cov2 infection. Frontiers in Sports and Active Living. 2022;4. doi: 10.3389/fspor.2022.877188 35847457 PMC9283867

[45] Capin JJ, Jolley SE, Morrow M, Connors M, Hare K, MaWhinney S, et al. Safety, feasibility and initial efficacy of an app-facilitated telerehabilitation (after) programme for covid-19 survivors: A pilot randomised study. BMJ Open. 2022;12(7). doi: 10.1136/bmjopen-2022-061285 35882451 PMC9329728

[46] Simpson AJ, Green A, Nettleton M, Hyde L, Shepherdson J, Killingback C, et al. Group-based pulmonary telerehabilitation is feasible, safe, beneficial and well-received in patients who have been hospitalised with covid-19. ERJ Open Research. 2022;9(2):00373–2022. doi: 10.1183/23120541.00373-2022 PMC9703872 36915803

[47] Rosen K, Patel M, Lawrence C, Mooney B. Delivering telerehabilitation to covid-19 inpatients:A Retrospective Chart Review suggests it is a viable option. HSS Journal ^®^. 2020;16(S1):64–70. doi: 10.1007/s11420-020-09774-4 32837409 PMC7364751

[48] Mayer KP, Parry SM, Kalema AG, Joshi RR, Soper MK, Steele AK, et al. Safety and feasibility of an interdisciplinary treatment approach to optimize recovery from critical coronavirus disease 2019. Critical Care Explorations. 2021;3(8). doi: 10.1097/CCE.0000000000000516 34476403 PMC8378791

[49] Martin I, Braem F, Baudet L, Poncin W, Fizaine S, Aboubakar F, et al. Follow-up of functional exercise capacity in patients with covid-19: It is improved by Telerehabilitation. Respiratory Medicine. 2021;183:106438. doi: 10.1016/j.rmed.2021.106438 33964817 PMC8084600

[50] Holland AE, Hill CJ, Rochford P, Fiore J, Berlowitz DJ, Mcdonald CF. Telerehabilitation for people with chronic obstructive pulmonary disease: Feasibility of a simple, real time model of supervised exercise training. Journal of Telemedicine and Telecare. 2013;19(4):222–6. doi: 10.1177/1357633x13487100 23666438

[51] Alwakeel AJ, Sicondolfo A, Robitaille C, Bourbeau J, Saad N. The accessibility, feasibility, and safety of a standardized community-based tele-pulmonary rehab program for chronic obstructive pulmonary disease: A 3-year real-world prospective study. Annals of the American Thoracic Society. 2022 Jan;19(1):39–47. doi: 10.1513/AnnalsATS.202006-638OC 34170802

[52] Rosenbek Minet L, Hansen LW, Pedersen CD, Titlestad IL, Christensen JK, Kidholm K, et al. Early telemedicine training and counselling after hospitalization in patients with severe chronic obstructive pulmonary disease: A feasibility study. BMC Medical Informatics and Decision Making. 2015;15(1). doi: 10.1186/s12911-014-0124-4 25886014 PMC4336686

[53] Hume E, Muse H, Wallace K, Wilkinson M, Heslop Marshall K, Nair A, et al. Feasibility and acceptability of a physical activity behavioural modification tele-coaching intervention in lung transplant recipients. Chronic Respiratory Disease. 2022;19:147997312211165. doi: 10.1177/14799731221116588 36306548 PMC9619269

[54] Diamond JM, Courtwright AM, Balar P, Oyster M, Zaleski D, Adler J, et al. Mobile health technology to improve emergent frailty after lung transplantation. Clinical Transplantation. 2021;35(4). doi: 10.1111/ctr.14236 33527520

[55] Choi J, Hergenroeder AL, Burke L, Dabbs AD, Morrell M, Saptono A, et al. Delivering an in-home exercise program via telerehabilitation: A pilot study of lung transplant go (LTGO). International Journal of Telerehabilitation. 2016;8(2):15–26. doi: 10.5195/ijt.2016.6201 28775798 PMC5536726

[56] Cox NS, McDonald CF, Mahal A, Alison JA, Wootton R, Hill CJ, et al. Telerehabilitation for chronic respiratory disease: A randomised controlled equivalence trial. Thorax. 2021;77(7):643–51. doi: 10.1136/thoraxjnl-2021-216934 34650004

[57] Coats V, Moffet H, Vincent C, Simard S, Tremblay L, Maltais F, et al. Feasibility of an eight-week telerehabilitation intervention for patients with unresectable thoracic Neoplasia receiving chemotherapy: A pilot study. Canadian Journal of Respiratory, Critical Care, and Sleep Medicine. 2019;4(1):14–24. doi: 10.1080/24745332.2019.1575703

[58] Layton AM, Irwin AM, Mihalik EC, Fleisch E, Keating CL, DiMango EA, et al. Telerehabilitation using fitness application in patients with severe cystic fibrosis awaiting Lung Transplant: A pilot study. International Journal of Telemedicine and Applications. 2021;2021:1–7. doi: 10.1155/2021/6641853 33727918 PMC7935590

[59] Kringle EA, Setiawan IM, Golias K, Parmanto B, Skidmore ER. Feasibility of an iterative rehabilitation intervention for stroke delivered remotely using Mobile Health Technology. Disability and Rehabilitation: Assistive Technology. 2019;15(8):908–16. doi: 10.1080/17483107.2019.1629113 31216917 PMC6920604

[60] Jarbandhan A, Toelsie J, Veeger D, Bipat R, Vanhees L, Buys R. Feasibility of a home-based physiotherapy intervention to promote post-stroke mobility: A randomized controlled pilot study. PLOS ONE. 2022;17(3). doi: 10.1371/journal.pone.0256455 35255091 PMC8901054

[61] Held JP, Ferrer B, Mainetti R, Steblin A, Hertler B, Moreno-Conde A, et al. Autonomous Rehabilitation at stroke patients home for balance and gait: Safety, usability and compliance of a virtual reality system. European Journal of Physical and Rehabilitation Medicine. 2018;54(4). doi: 10.23736/S1973-9087.17.04802-X 28949120

[62] Galloway M, Marsden DL, Callister R, Nilsson M, Erickson KI, English C. The feasibility of a telehealth exercise program aimed at increasing cardiorespiratory fitness for people after stroke. International Journal of Telerehabilitation. 2019;11(2):9–28. doi: 10.5195/ijt.2019.6290 35949926 PMC9325643

[63] Gagnon M-A, Batcho CS, Bird M-L, Labbé B, Best KL. Feasibility of a remotely supervised home-based group eHealth Fitness and Mobility Exercise Program for stroke: French-canadian version preliminary study. Topics in Stroke Rehabilitation. 2022;30(2):169–79. doi: 10.1080/10749357.2021.2012008 34994303

[64] Edwards D, Kumar S, Brinkman L, Ferreira IC, Esquenazi A, Nguyen T, et al. Telerehabilitation initiated early in Post-Stroke Recovery: A Feasibility Study. Neurorehabilitation and Neural Repair. 2023;37(2–3):131–41. doi: 10.1177/15459683231159660 36876946 PMC10080366

[65] Van de Winckel A, Carey JR, Bisson TA, Hauschildt EC, Streib CD, Durfee WK. Home-based transcranial direct current stimulation plus tracking training therapy in people with stroke: An open-label Feasibility Study. Journal of NeuroEngineering and Rehabilitation. 2018;15(1). doi: 10.1186/s12984-018-0427-2 30227864 PMC6145321

[66] Simpson DB, Bird M-L, English C, Gall SL, Breslin M, Smith S, et al. “connecting patients and therapists remotely using technology is feasible and facilitates exercise adherence after stroke.” Topics in Stroke Rehabilitation. 2019;27(2):93–102. doi: 10.1080/10749357.2019.1690779 31762412

[67] Qiu Q, Cronce A, Patel J, Fluet GG, Mont AJ, Merians AS, et al. Development of the home based Virtual Rehabilitation System (HoVRS) to remotely deliver an intense and customized upper extremity training. Journal of NeuroEngineering and Rehabilitation. 2020;17(1). doi: 10.1186/s12984-020-00789-w 33228709 PMC7685660

[68] Palmcrantz S, Borg J, Sommerfeld D, Plantin J, Wall A, Ehn M, et al. An interactive distance solution for stroke rehabilitation in the home setting–a feasibility study. Informatics for Health and Social Care. 2016;42(3):303–20. doi: 10.1080/17538157.2016.1253015 27918220

[69] Benvenuti F, Stuart M, Cappena V, Gabella S, Corsi S, Taviani A, et al. Community-based exercise for upper limb paresis: a controlled trial with telerehabilitation. Neurorehabilitation and Neural Repair. 2014;28(7):611–20. doi: 10.1177/1545968314521003 24515928

[70] Wilson PH, Rogers JM, Vogel K, Steenbergen B, McGuckian TB, Duckworth J. Home-based (virtual) rehabilitation improves motor and cognitive function for stroke patients: A randomized controlled trial of the elements (Edna-22) system. Journal of NeuroEngineering and Rehabilitation. 2021;18(1). doi: 10.1186/s12984-021-00956-7 34823545 PMC8613521

[71] Øra HP, Kirmess M, Brady MC, Partee I, Hognestad RB, Johannessen BB, et al. The effect of augmented speech-language therapy delivered by telerehabilitation on poststroke aphasia—a pilot randomized controlled trial. Clinical Rehabilitation. 2020;34(3):369–81. doi: 10.1177/0269215519896616 31903800

[72] Piraux E, Caty G, Reychler G, Forget P, Deswysen Y. Feasibility and preliminary effectiveness of a tele-prehabilitation program in Esophagogastric Cancer patients. Journal of Clinical Medicine. 2020;9(7):2176. doi: 10.3390/jcm9072176 32660126 PMC7408844

[73] van Egmond MA, Engelbert RH, Klinkenbijl JH, van Berge Henegouwen MI, van der Schaaf M. Physiotherapy with telerehabilitation in patients with complicated postoperative recovery after esophageal cancer surgery: Feasibility study. Journal of Medical Internet Research. 2020;22(6). doi: 10.2196/16056 32515742 PMC7312239

[74] Chen K, Yao F, Chen X, Lin Y, Kang M. Effectiveness of telerehabilitation on short-term quality of life of patients after esophageal cancer surgery during COVID-19: A single-center, randomized, controlled study. Journal of Gastrointestinal Oncology. 2021;12(4):1255–64. doi: 10.21037/jgo-21-385 34532085 PMC8421896

[75] Parraguez LA, Ribeiro IL, Hinojosa MP, Troncoso JP. Implementation of a teleprehabilitation program for oncosurgical patients during the COVID-19 pandemic: Perspectives and User Satisfaction. Supportive Care in Cancer. 2023;31(6). doi: 10.1007/s00520-023-07799-z 37212973 PMC10201043

[76] Dennett A, Harding KE, Reimert J, Morris R, Parente P, Taylor NF. Telerehabilitation’s safety, feasibility, and exercise uptake in cancer survivors: Process evaluation. JMIR Cancer. 2021;7(4). doi: 10.2196/33130 34854817 PMC8768007

[77] Gehring K, Kloek CJ, Aaronson NK, Janssen KW, Jones LW, Sitskoorn MM, et al. Feasibility of a home-based exercise intervention with remote guidance for patients with Stable Grade II and III gliomas: A pilot randomized controlled trial. Clinical Rehabilitation. 2017;32(3):352–66. doi: 10.1177/0269215517728326 28882061 PMC6625754

[78] Filakova K, Janikova A, Felsoci M, Dosbaba F, Su JJ, Pepera G, et al. Home-based cardio-oncology rehabilitation using a telerehabilitation platform in hematological cancer survivors: A feasibility study. BMC Sports Science, Medicine and Rehabilitation. 2023;15(1). doi: 10.1186/s13102-023-00650-2 36959613 PMC10034898

[79] Kim Y, Chae H, Park SJ. Feasibility and benefits of a videoconferencing‐based Home Exercise Programme for paediatric cancer survivors during the coronavirus disease 2019 pandemic. European Journal of Cancer Care. 2022;31(5). doi: 10.1111/ecc.13624 35606331 PMC9347713

[80] Lavoie V, Bouchard M, Turcotte S, Tousignant M. Telerehabilitation for individuals with parkinson’s disease and a history of Falls: A pilot study. Physiotherapy Canada. 2021;73(4):343–50. doi: 10.3138/ptc-2019-0108 34880539 PMC8614589

[81] Kwok JY, Lee JJ, Choi EP, Chau PH, Auyeung M. Stay mindfully active during the coronavirus pandemic: A feasibility study of mhealth-delivered mindfulness yoga program for people with parkinson’s disease. BMC Complementary Medicine and Therapies. 2022;22(1). doi: 10.1186/s12906-022-03519-y 35130894 PMC8818838

[82] James-Palmer AM, Daneault J-F. Tele-Yoga for the management of parkinson disease: A safety and feasibility trial. DIGITAL HEALTH. 2022;8:205520762211193. doi: 10.1177/20552076221119327 35990111 PMC9386843

[83] Cooley Hidecker MJ, Landers MR, Piccorelli A, Bush E, Singh R. Coordinated speech therapy, physiotherapy, and Pharmaceutical Care Telehealth for people with parkinson disease in rural communities: An exploratory, 8-week cohort study for feasibility, safety, and signal of efficacy. Rural and Remote Health. 2022; doi: 10.22605/rrh6679 35026120

[84] Colón-Semenza C, Zajac JA, Schwartz A, Darbandsari P, Ellis TD. Experiences from the implementation of physical therapy via telehealth for individuals with parkinson disease during the COVID-19 pandemic. Disability and Rehabilitation. 2023;1–9. doi: 10.1080/09638288.2023.2202418 37088939

[85] Bianchini E, Onelli C, Morabito C, Alborghetti M, Rinaldi D, Anibaldi P, et al. Feasibility, safety, and effectiveness of telerehabilitation in mild-to-moderate parkinson’s disease. Frontiers in Neurology. 2022;13. doi: 10.3389/fneur.2022.909197 35785358 PMC9245570

[86] van der Kolk NM, de Vries NM, Kessels RP, Joosten H, Zwinderman AH, Post B, et al. Effectiveness of home-based and remotely supervised aerobic exercise in parkinson’s disease: A double-blind, randomised controlled trial. The Lancet Neurology. 2019;18(11):998–1008. doi: 10.1016/S1474-4422(19)30285-6 31521532

[87] Seidler KJ, Duncan RP, McNeely ME, Hackney ME, Earhart GM. Feasibility and preliminary efficacy of a telerehabilitation approach to group adapted tango instruction for people with parkinson disease. Journal of Telemedicine and Telecare. 2016;23(8):740–6. doi: 10.1177/1357633X16668092 27624469

[88] Farr WJ, Green D, Bremner S, Male I, Gage H, Bailey S, et al. Feasibility of a randomised controlled trial to evaluate home-based virtual reality therapy in children with cerebral palsy. Disability and Rehabilitation. 2019;43(1):85–97. doi: 10.1080/09638288.2019.1618400 31131641

[89] Schlichting T, Martins da Silva K, Silva Moreira R, Marques de Moraes MV, Cicuto Ferreira Rocha NA, Boyd RN, et al. Telehealth program for infants at risk of cerebral palsy during the COVID-19 pandemic: A pre-post Feasibility Experimental Study. Physical & Occupational Therapy In Pediatrics. 2022;42(5):490–509. doi: 10.1080/01942638.2022.2057209 35341469

[90] Donkers SJ, Nickel D, Paul L, Wiegers SR, Knox KB. Adherence to physiotherapy-guided web-based exercise for persons with moderate-to-severe multiple sclerosis. International Journal of MS Care. 2020;22(5):208–14. doi: 10.7224/1537-2073.2019-048 33177956 PMC7643843

[91] Paul L, Renfrew L, Freeman J, Murray H, Weller B, Mattison P, et al. Web-based physiotherapy for people affected by multiple sclerosis: A single blind, randomized controlled feasibility study. Clinical Rehabilitation. 2018;33(3):473–84. doi: 10.1177/0269215518817080 30514108

[92] Sari YM, Burton E, Lee D-CA, Hill KD. A telehealth home-based exercise program for community-dwelling older people with dementia in Indonesia: A feasibility study. International Journal of Environmental Research and Public Health. 2023;20(4):3397. doi: 10.3390/ijerph20043397 36834093 PMC9966659

[93] Sheehy L, Sveistrup H, Knoefel F, Taillon-Hobson A, Martin T, Egan M, et al. The use of home-based Nonimmersive virtual reality to encourage physical and cognitive exercise in people with mild cognitive impairment: A feasibility study. Journal of Aging and Physical Activity. 2022;30(2):297–307. doi: 10.1123/japa.2021-0043 34453024

[94] Campbell KR, Wilhelm JL, Pettigrew NC, Scanlan KT, Chesnutt JC, King LA. Implementation and adoption of telerehabilitation for treating mild traumatic brain injury. Journal of Neurologic Physical Therapy. 2022;46(4). doi: 10.1097/NPT.0000000000000409 35666882 PMC12927607

[95] Ackerley S, Wilson N, Boland P, Read J, Connell L. Implementation of neurological group-based telerehabilitation within existing healthcare during the COVID-19 pandemic: A mixed methods evaluation. BMC Health Services Research. 2023 Jun 21;23(1). doi: 10.1186/s12913-023-09635-w 37344774 PMC10283243

[96] Coronado RA, Devin CJ, Pennings JS, Aaronson OS, Haug CM, Van Hoy EE, et al. Safety and feasibility of an early telephone-supported home exercise program after anterior cervical discectomy and fusion: A case series. Physiotherapy Theory and Practice. 2019;37(10):1096–108. doi: 10.1080/09593985.2019.1683921 31663795 PMC7737349

[97] Plaza A, Paratz J, Cottrell M. A six-week physical therapy exercise program delivered via home-based telerehabilitation is comparable to in-person programs for patients with burn injuries: A randomized, controlled, non-inferiority clinical pilot trial. Burns. 2023;49(1):55–67. doi: 10.1016/j.burns.2022.08.014 36115795

[98] Fioratti I, Miyamoto GC, Fandim JV, Ribeiro CP, Batista GD, Freitas GE, et al. Feasibility, usability, and implementation context of an internet-based pain education and exercise program for chronic musculoskeletal pain: Pilot trial of the REABILITADOR program. JMIR Formative Research. 2022;6(8). doi: 10.2196/35743 35776863 PMC9472033

[99] Pfister PB, Knols RH, de Bie RA, de Bruin ED. Feasibility of a blended therapy approach in the treatment of patients with inflammatory myopathies. Archives of Physiotherapy. 2021;11(1). doi: 10.1186/s40945-021-00108-z 34039438 PMC8157458

[100] Correia FD, Nogueira A, Magalhães I, Guimarães J, Moreira M, Barradas I, et al. Home-based rehabilitation with a novel digital biofeedback system versus conventional in-person rehabilitation after total knee replacement: A feasibility study. Scientific Reports. 2018;8(1). doi: 10.1038/s41598-018-29668-0 30050087 PMC6062628

[101] Moriichi K, Fujiya M, Ro T, Ota T, Nishimiya H, Kodama M, et al. A novel telerehabilitation with an educational program for caregivers using telelecture is feasible for fall prevention in elderly people. Medicine. 2022;101(6). doi: 10.1097/md.0000000000027451 35147084 PMC8830826

[102] Ozturk B, Duruturk N. Effect of telerehabilitation applied during COVID-19 isolation period on physical fitness and quality of life in overweight and obese individuals. International Journal of Obesity. 2021;46(1):95–9. doi: 10.1038/s41366-021-00965-5 34504288 PMC8426585

[103] Cerdan de las Heras J, Balbino F, Catalán-Matamoros D, Hilberg O, Løkke A, Bendstrup E. Effect of a telerehabilitation program in sarcoidosis. Sarcoidosis Vasc Diffuse Lung Dis [Internet]. 2022 Mar. 31 [cited 2023 Dec. 23];39(1):e2022003. Available from: https://www.mattioli1885journals.com/index.php/sarcoidosis/article/view/12526 doi: 10.36141/svdld.v39i1.12526 35494172 PMC9007024

[104] Piraux E, Reychler G, Forget P, Yombi J-C, Caty G. Feasibility and preliminary effects of a telerehabilitation program for people living with HIV. Journal of the Association of Nurses in AIDS Care. 2019;30(2):176–85. doi: 10.1097/jnc.0000000000000005 30822290

[105] Wong MY, Gunasekeran DV, Nusinovici S, Sabanayagam C, Yeo KK, Cheng C-Y, et al. Telehealth demand trends during the COVID-19 pandemic in the top 50 most affected countries: Infodemiological evaluation. JMIR Public Health and Surveillance. 2021;7(2). doi: 10.2196/24445 33605883 PMC7899203

[106] Shaver J. The state of telehealth before and after the COVID-19 pandemic. Primary Care: Clinics in Office Practice. 2022;49(4):517–30. doi: 10.1016/j.pop.2022.04.002 36357058 PMC9035352

[107] Embedding equity into every step of adverse event analysis [Internet]. Institute for Healthcare Improvement; 2023 [cited 2023 Sept 10]. https://www.ihi.org/communities/blogs/embedding-equity-into-every-step-of-adverse-event-analysis

[108] Munce S, Andreoli A, Bayley M, Guo M, Inness EL, Kua A, et al. Clinicians’ experiences of implementing a telerehabilitation toolkit during the COVID-19 pandemic: Qualitative Descriptive Study. JMIR Rehabilitation and Assistive Technologies. 2023;10. doi: 10.2196/44591 36897634 PMC10039414

[109] Blandford A, Wesson J, Amalberti R, AlHazme R, Allwihan R. Opportunities and challenges for telehealth within, and beyond, a pandemic. The Lancet Global Health. 2020;8(11). doi: 10.1016/s2214-109x(20)30362-4 32791119 PMC7417162

[110] College Update: Expectations for Tele-rehabilitation [Internet]. College of Physiotherapists of Ontario; 2020 [cited 2023 Sept 10]. https://www.collegept.org/registrants/virtual-practice-in-physiotherapy

[111] New Cross-Border & Tele-Rehabilitation Guidelines [Internet]. Canadian Alliance of Physiotherapy Regulators; 2018 [cited 2023 Sept 10]. https://alliancept.org/announcement/new-cross-border-tele-rehabilitation-guidelines/

[112] Deshpande A, Khoja S, Lorca J, McKibbon A, Rizo C, Husereau D, et al. Asynchronous telehealth: A scoping review of analytic studies [Internet]. U.S. National Library of Medicine; 2009 [cited 2023 Sept 3]. https://www.ncbi.nlm.nih.gov/pmc/articles/PMC2765770/PMC2765770 19946396

[113] Govender I, Nashed KK, Rangiah S, Okeke S, Maphasha OM. Palpitations: Evaluation and management by Primary Care Practitioners. South African Family Practice. 2022;64(1). doi: 10.4102/safp.v64i1.5449 35261258 PMC8905373

[114] Ford TJ, Berry C. Angina: Contemporary diagnosis and management. Heart. 2020;106(5):387–98. doi: 10.1136/heartjnl-2018-314661 32054665 PMC7035719

[115] Choi J, Lee J, Cho JW, Koh S, Yang YS, Yoo D, et al. Double‐Blind, Randomized, Placebo‐Controlled Trial of DA‐9701 in Parkinson’s Disease: PASS‐GI Study. Movement Disorders. 2020;35(11):1966–76. doi: 10.1002/mds.28219 32761955 PMC7754502

[116] Velez M, Lugo-Agudelo LH, Patiño Lugo DF, Glenton C, Posada AM, Mesa Franco LF, et al. Factors that influence the provision of home-based rehabilitation services for people needing rehabilitation: A qualitative evidence synthesis. Cochrane Database of Systematic Reviews. 2023;2023(2). doi: 10.1002/14651858.CD014823 36780267 PMC9918343

[117] Levy BB, Luong D, Perrier L, Bayley MT, Munce SE. Peer support interventions for individuals with acquired brain injury, Cerebral Palsy, and SPINA BIFIDA: A systematic review. BMC Health Services Research. 2019;19(1). doi: 10.1186/s12913-019-4110-5 31068184 PMC6505073

[118] KITE Research Institute at UHN. Implementing telerehabilitation within outpatient rehabilitation programs (TR-Telerehab Toolkit) [Internet]. KITE Research Institute at UHN; 2024 [cited 2024 Feb 5]. https://kite-uhn.com/tools/tr-telerehab-toolkit

[119] Prvu Bettger J, Resnik LJ. Telerehabilitation in the age of covid-19: An opportunity for Learning Health System Research. Physical Therapy. 2020;100(11):1913–6. doi: 10.1093/ptj/pzaa151 32814976 PMC7454917

