# Supplementary material for: Adverse events associated with the delivery of telerehabilitation across rehabilitation populations: A scoping review
Source: PLoS One. 2024 Nov 19;19(11):e0313440. doi: 10.1371/journal.pone.0313440 (PMC11575805; doi:10.1371/journal.pone.0313440)
Supplement: S2 Appendix — (PDF) [file pone.0313440.s002.pdf]

S2. Appendix. MEDLINE(R) ALL 1946 to June 22, 2023 Search strategy.

#	Searches	Results
1	Telemedicine/	37260
2	Videoconferencing/	2320
3	Remote Consultation/	5725
4	or/1-3	42884
5	exp Rehabilitation/	352248
6	4 and 5	3150
7	rehab*.tw,kf.	221905
8	4 and 7	1207
9	rh.fs.	208266
10	4 and 9	952
11	(remote* or tele* or virtual* or "vr" or videoconferenc* or "video conferenc*").tw,kf.	469375
12	(5 or 7) and 11	18196
13	or/6,8,10,12	20021
14	Telerehabilitation/	981
15	telerehab*.tw,kf.	1934
16	"e-rehab*".tw,kf.	20
17	or/13-16	20426
18	Safety/	41951
19	Patient Safety/	25400
20	(safe or safety or safeties or safely or unsafe).tw,kf.	1102853
21	Patient Harm/	219
22	(harm or harmed or harmful or harming or harms).tw,kf.	165679
23	(risk or risks).tw,kf.	2880460
24	Medical Errors/	17727
25	(adverse* adj5 (effect* or event* or incident or incidents or outcome*)).tw,kf.	553468
26	(critical adj5 (event* or incident or incidents or outcome*)).tw,kf.	22448
27	(negativ* adj5 (effect* or event* or impact* or incident or incidents or mistake or mistakes or outcome*)).tw,kf.	210509
28	(therap* adj5 (accident* or error or errors or excessive or incident or incidents or mistake or mistakes or unnecessary or wrong)).tw,kf.	7307
29	(outcome* adj5 harm*).tw,kf.	2906
30	complication*.tw,kf.	1187914
31	ae.fs.	1989850
32	or/18-31	6197051
33	17 and 32	5212
34	limit 33 to english language	5033
35	limit 34 to yr="2013 -Current"	3857
