# Supplementary material for: Adverse events associated with the delivery of telerehabilitation across rehabilitation populations: A scoping review
Source: PLoS One. 2024 Nov 19;19(11):e0313440. doi: 10.1371/journal.pone.0313440 (PMC11575805; doi:10.1371/journal.pone.0313440)
Supplement: S3 Appendix — (DOCX) [file pone.0313440.s003.docx]

| **Title** | **Authors** | **Published Year** | **Reason for Exclusion** |
| --- | --- | --- | --- |
| Feasibility of an individually tailored virtual reality program for improving upper motor functions and activities of daily living in chronic stroke survivors: A case series | Kim et al. | 2016 | ineligible intervention |
| Development and evaluation of a new telerehabilitation system based on VR technology using multisensory feedback for patients with stroke | Kato et al. | 2015 | outcomes not relevant |
| Adverse events in mobility-limited and chronically ill elderly adults participating in an exercise intervention study supported by general practitioner practices | Hinrichs et al. | 2015 | ineligible intervention |
| Safety, Feasibility, and Acceptability of a New Virtual Rehabilitation Platform: A Supervised Pilot Study | Escalante-Gonzalbo et al. | 2021 | ineligible intervention |
| Feasibility of incorporating functionally relevant virtual rehabilitation in sub-acute stroke care: Perception of patients and clinicians | Demers et al. | 2019 | ineligible intervention |
| Baduanjin qigong intervention by telerehabilitation (Teleparkinson): A proof-of-concept study in parkinson's disease | Carvalho et al. | 2021 | ineligible study design |
| Feasibility and safety of the 30-second sit-to-stand test delivered via telehealth: An observational study | Bowman et al. | 2023 | ineligible intervention |
| Postoperative compliance and return to work after rotator cuff repair: value of an interactive online rehabilitation program among patients treated under workers' compensation | Anderson et al. | 2021 | outcomes not relevant |
| Using a virtual game system to innovate pulmonary rehabilitation: Safety, adherence and enjoyment in severe chronic obstructive pulmonary disease | Wardini et al. | 2013 | ineligible intervention |
| Home-based preoperative rehabilitation (prehab) to improve physical function and reduce hospital length of stay for frail patients undergoing coronary artery bypass graft and valve surgery | Waite et al. | 2017 | ineligible intervention |
| Feasibility and safety of virtual-reality-based early neurocognitive stimulation in critically ill patients | Turon et al. | 2017 | ineligible intervention |
| A randomized controlled trial of postoperative rehabilitation using digital healthcare system after rotator cuff repair | Shim et al. | 2023 | ineligible intervention |
| Efficacy of Neck-Specific Exercise With Internet Support Versus Neck-Specific Exercise at a Physiotherapy Clinic in Chronic Whiplash-Associated Disorders: Multicenter Randomized Controlled Noninferiority Trial | Peterson et al. | 2023 | ineligible intervention |
| Development and feasibility of a modified Fugl-Meyer lower extremity assessment for telerehabilitation: a pilot study | Peters et al. | 2021 | ineligible intervention |
| E-Health Exercise Intervention for Pediatric Patients with Congenital Heart Disease: a Randomized Controlled Trial | Meyer et al. | 2021 | ineligible intervention |
| Internet and Telerehabilitation-Delivered Management of Rotator Cuff-Related Shoulder Pain (INTEL Trial): randomized Controlled Pilot and Feasibility Trial | Malliaras et al. | 2020 | ineligible intervention |
| BioTrak virtual reality system: effectiveness and satisfaction analysis for balance rehabilitation in patients with brain injury | Llorens et al. | 2013 | ineligible intervention |
| Safety and Potential Usability of Immersive Virtual Reality for Brain Rehabilitation: A Pilot Study | Lim et al. | 2023 | ineligible intervention |
| Safety and feasibility of a telemonitoring-guided exercise program in patients receiving cardiac resynchronization therapy | Koike et al. | 2022 | outcomes not relevant |
| Safety, feasibility, and effectiveness of virtual pulmonary rehabilitation in the real world | Knox et al. | 2019 | outcomes not relevant |
| The Design, Development, and Testing of a Virtual Reality Device for Upper Limb Training in People With Multiple Sclerosis: Single-Center Feasibility Study | Kalron et al. | 2022 | ineligible intervention |
| Effect of Telerehabilitation Versus In-Clinic Rehabilitation Delivery on Self-Efficacy in Breast Cancer-Related Lymphedema | Helm et al. | 2023 | outcomes not relevant |
| Vestibular Rehabilitation Telehealth During the SAEA-CoV-2 (COVID-19) Pandemic | Harrell et al. | 2022 | outcomes not relevant |
| Feasibility of Virtual Reality Exercises at Home for Post-COVID-19 Condition: Cohort Study | Groenveld et al. | 2022 | ineligible intervention |
| Short-Term Effects of a Respiratory Telerehabilitation Program in Confined COVID-19 Patients in the Acute Phase: a Pilot Study | Gonzalez-Gerez et al. | 2021 | outcomes not relevant |
| Feasibility of a virtual exercise coach to promote walking in community-dwelling persons with Parkinson disease | Ellis et al. | 2013 | ineligible intervention |
| Effects of Pilates-based telerehabilitation on physical performance and quality of life in patients with multiple sclerosis | Eldemir et al. | 2023 | outcomes not relevant |
| Effect of tele-rehabilitation on glucose control, exercise capacity, physical fitness, muscle strength and psychosocial status in patients with type 2 diabetes: A double blind randomized controlled trial | Duruturk et al. | 2019 | outcomes not relevant |
| Feasibility and preliminary effects of a tele-prehabilitation program and an in-person prehablitation program compared to usual care for total hip or knee arthroplasty candidates: a pilot randomized controlled trial | Doiron-Cadrin et al. | 2020 | ineligible intervention |
| Feasibility of virtual reality augmented cycling for health promotion of people poststroke | Deutsh et al. | 2013 | ineligible intervention |
| Effect of Virtual Reality Gait Training on Participation in Survivors of Subacute Stroke: A Randomized Controlled Trial | de Rooij et al. | 2021 | ineligible intervention |
| Virtual reality-based therapy for the treatment of balance deficits in patients receiving inpatient rehabilitation for traumatic brain injury | Cuthbert et al. | 2014 | ineligible intervention |
| Rapid Development and Implementation of Telerehabilitation at Shirley Ryan Abilitylab | Curran et al. | 2022 | ineligible study design |
| Mobile game-based virtual reality rehabilitation program for upper limb dysfunction after ischemic stroke | Choi et al. | 2016 | ineligible intervention |
| Home-Based Arm Cardiac Rehabilitation in Disabled Veterans: A Pilot Study | Chockalingam et al. | 2021 | ineligible study design |
| Feasibility, Acceptability, and Efficacy of Virtual Reality Training for Older Adults and People With Disabilities: Single-Arm Pre-Post Study | Chau et al. | 2021 | ineligible intervention |
| Immersive Virtual Reality for the Cognitive Rehabilitation of Stroke Survivors | Chatterjee et al. | 2022 | ineligible intervention |
| Improving motor performance in Parkinson's disease: a preliminary study on the promising use of the computer assisted virtual reality environment (CAREN) | Calabrò et al. | 2020 | ineligible intervention |
| Telerehabilitation for physical disabilities and movement impairment: A service evaluation in South West England | Buckingham et al. | 2022 | outcomes not relevant |
| Telerehabilitation: Future of Phase II Cardiac Rehabilitation: Review of Preliminary Outcomes | Bryant et al. | 2022 | ineligible study design |
| Cardiac telerehabilitation as an alternative to centre-based cardiac rehabilitation | Brouwers et al. | 2020 | ineligible study design |
| Feasibility of Group-Based Multiple Virtual Reality Sessions to Reduce Behavioral and Psychological Symptoms in Persons Living in Residential Aged Care | Brimelow et al. | 2022 | ineligible intervention |
| Effectiveness and safety of a home-based cardiac rehabilitation programme of mixed surveillance in patients with ischemic heart disease at moderate cardiovascular risk: A randomised, controlled clinical trial | Bravo-Escobar et al. | 2017 | outcomes not relevant |
| Patient-reported outcomes measures (PROMs) and patient-reported experience measures (PREMs) of COVID-19 telerehabilitation: Prospective pilot program | Bordas-Martinez et al. | 2022 | outcomes not relevant |
| Immersive Virtual Reality for the Management of Pain in Community-Dwelling Older Adults | Benham et al. | 2019 | ineligible intervention |
| Long-term exercise effects after cardiac telerehabilitation in patients with coronary artery disease: 1-year follow-up results of the randomized study | Batalik et al. | 2021 | outcomes not relevant |
| "It's second best": A mixed-methods evaluation of the experiences and attitudes of people with musculoskeletal pain towards physiotherapist delivered telehealth during the COVID-19 pandemic | Barton et al. | 2022 | outcomes not relevant |
| The state of the art in telerehabilitation for musculoskeletal conditions | Baroni et al. | 2023 | ineligible study design |
| Immersive Virtual Tasks with Motor and Cognitive Components: A Feasibility Study of Adults and Older Adult Fallers and Nonfallers | Bacha et al. | 2023 | ineligible intervention |
| Remote cardiac rehabilitation services and the digital divide: implications for elderly populations during the COVID19 pandemic | Astley et al. | 2021 | ineligible study design |
| Acceptance and Usability of Immersive Virtual Reality in Older Adults with Objective and Subjective Cognitive Decline | Arlati et al. | 2021 | ineligible intervention |
| Challenges and Facilitators to Telehealth Occupational Therapy for Autistic Children During COVID-19 | Angell et al. | 2023 | outcomes not relevant |
| Safety and effectiveness of telerehabilitation program in people with severe haemophilia in Chile. A qualitative study | Aliaga-Castillo et al. | 2022 | outcomes not relevant |
| The Surrey Virtual Reality System for the (gait) rehabilitation of children with cerebral palsy: Pilot perspectives from young able-bodied adult users | Al-Amri et al. | 2014 | ineligible study design |
| The Effect of Adding Virtual Reality Training on Traditional Exercise Program on Balance and Gait in Unilateral, Traumatic Lower Limb Amputee | Abbas et al. | 2021 | ineligible intervention |
| Tele-rehabilitation Program: an Innovative and Sustainable Early Intervention Service for Children With Autism Spectrum Disorders | no author listed | 2023 | ineligible study design |
| Telerehabilitation in Hemato-oncological Survivors | no author listed | 2023 | ineligible study design |
| A trial of integrated arm and language rehabilitation in people living in the community with arm and communication impairments 3 to 24 months post stroke | no author listed | 2022 | ineligible study design |
| An Integrative Cardiac Rehabilitation Employing Smartphone Technology (iCREST) | no author listed | 2022 | ineligible study design |
| Effectiveness of a Telerehabilitation Program in Ankle Sprain | no author listed | 2022 | ineligible study design |
| The effects of Home Rehabilitation compared to Face-to-face Rehabilitation in people with Peripheral Arterial Disease | no author listed | 2022 | ineligible study design |
| Investigating the impact of new models of rehabilitation on work and health outcomes after stroke | no author listed | 2022 | ineligible study design |
| TeleCaRe: expanding delivery of Cancer Rehabilitation via telehealth | no author listed | 2021 | ineligible study design |
| Telerehabilitation With Aims to Improve Lower Extremity Recovery Post-Stroke (TRAIL-PILOT) | no author listed | 2021 | ineligible study design |
| Tele-rehabilitation Program After Hospitalization for COVID-19 | no author listed | 2021 | ineligible study design |
| Telehealth for Falls and Fracture Prevention Implementation Trial (TeleFFIT) | no author listed | 2021 | ineligible study design |
| Effects of Wearable Sensor Based Virtual Reality Game on Balance for Stroke | no author listed | 2020 | ineligible study design |
| Telerehabilitation and internet-based management of rotator cuff related pain: a pilot and feasibility randomised controlled trial | no author listed | 2020 | ineligible study design |
| Home rehabilitation for people with COVID-19: implementing telehealth approaches to care | no author listed | 2020 | ineligible study design |
| Evaluation of the mobile pulmonary rehabilitation (m-PR) platform on exercise capacity and health status in people with chronic lung disease | no author listed | 2019 | ineligible study design |
| A Phase 2 Study to Investigate the Feasibility and Acceptability of the HOLOBalance System Compared to Standard Care in an Ageing Population With Balance Disorders | no author listed | 2019 | ineligible study design |
| Smartphone Cardiac Rehabilitation, Assisted self-Management (SCRAM): a 21st Century Approach for Improving the Self-Management of Heart Disease | no author listed | 2018 | ineligible study design |
| Feasibility of Telerehabilitation in HIV-patients | no author listed | 2017 | ineligible study design |
| Technological Platforms and Telerehabilitation in Heart Surgery | no author listed | 2016 | ineligible study design |
| A Randomised Controlled Study on the Maxm Skate: a Lower Limb Rehabilitation Device for use following Total Knee Arthroplasty | no author listed | 2016 | ineligible study design |
| Comparing Online Pulmonary Rehabilitation 'myPR' Versus Conventional Pulmonary Rehabilitation for Patients With COPD | no author listed | 2015 | ineligible study design |
| Collaborative Care Model Based Telerehabilitation Exercise Training Program for Acute Stroke Patients in China: a Randomized Controlled Trial | Wu et al. | 2020 | outcomes not relevant |
| Immersive Virtual Reality Mirror Therapy for Upper Limb Recovery After Stroke: A Pilot Study | Weber et al. | 2019 | ineligible intervention |
| Telerehabilitation of Subjects with Neurodevelopmental Disorders During Confinement due to COVID-19 | Vaucheret et al. | 2022 | not written in English language |
| Feasibility of virtual reality and treadmill training in traumatic brain injury: a randomized controlled pilot trial | Tefertiller et al. | 2022 | ineligible intervention |
| Effects of outpatient followed by home-based telemonitored cardiac rehabilitation in patients with coronary artery disease | Szalewska et al. | 2015 | outcomes not relevant |
| Effects and feasibility of virtual reality system vs traditional physical therapy training in multiple sclerosis patients | Streicher et al. | 2018 | ineligible intervention |
| A task-specific interactive game-based virtual reality rehabilitation system for patients with stroke: a usability test and two clinical experiments | Shin et al. | 2014 | ineligible intervention |
| Tele-pulmonary rehabilitation with face to face in COVID-19 pandemic: A hybrid modeling | Satar et al. | 2023 | outcomes not relevant |
| Combined Aerobic Exercise and Virtual Reality-Based Upper Extremity Rehabilitation Intervention for Chronic Stroke: Feasibility and Preliminary Effects on Physical Function and Quality of Life | Ross et al. | 2023 | ineligible intervention |
| Short-Term Effects of a Conditioning Telerehabilitation Program in Confined Patients Affected by COVID-19 in the Acute Phase. A Pilot Randomized Controlled Trial | Rodriguez-Blanco et al. | 2021 | outcomes not relevant |
| Safety and Feasibility of a First-Person View, Full-Body Interaction Game for Telerehabilitation Post-Stroke | Proffitt et al. | 2018 | ineligible intervention |
| Increasing upper limb training intensity in chronic stroke using embodied virtual reality: a pilot study | Perez-Marco et al. | 2017 | ineligible intervention |
| The Feasibility and Acceptability of Virtual Therapy Environments for Early ICU Mobilization | Parke et al. | 2020 | ineligible intervention |
| Effects of home-based virtual reality telerehabilitation system in people with multiple sclerosis: a randomized controlled trial | Pagliari et al. | 2021 | outcomes not relevant |
| Assessment of ECG during hybrid comprehensive telerehabilitation in heart failure patients-Subanalysis of the Telerehabilitation in Heart Failure Patients (TELEREH-HF) randomized clinical trial | Orzechowski et al. | 2021 | outcomes not relevant |
| Feasibility of hybrid telerehabilitation as a component of Managed Care after Acute Myocardial Infarction (MC-AMI) in a 12-month follow-up: experience from a single center | Orzechowski et al. | 2023 | outcomes not relevant |
| Feasibility, safety and efficacy of a virtual reality exergame system to supplement upper extremity rehabilitation post-stroke: A pilot randomized clinical trial and proof of principle | Norouzi-Gheidari et al. | 2020 | ineligible intervention |
| Design and Validation of Virtual Reality Task for Neuro-Rehabilitation of Distal Upper Extremities | Nath et al. | 2022 | ineligible intervention |
| Home-based cardiac rehabilitation using information and communication technology for heart failure patients with frailty | Nagatomi et al. | 2022 | outcomes not relevant |
| Effectiveness of home-based telerehabilitation in mild to moderate Alzheimer's disease: a randomised controlled study | Menengic et al. | 2021 | ineligible study design |
| A Home- and Community-Based Physical Activity Program Can Improve the Cardiorespiratory Fitness and Walking Capacity of Stroke Survivors | Marsden et al. | 2016 | ineligible intervention |
