# Supplementary material for: Adverse events associated with the delivery of telerehabilitation across rehabilitation populations: A scoping review
Source: PLoS One. 2024 Nov 19;19(11):e0313440. doi: 10.1371/journal.pone.0313440 (PMC11575805; doi:10.1371/journal.pone.0313440)
Supplement: S4 Appendix — (DOCX) [file pone.0313440.s004.docx]

| **Authors** | **Extractor #1** | **When extracted** | **Extractor #2** | **When extracted** |
| --- | --- | --- | --- | --- |
| Lundgren et al. [30] | TY | Aug 1-10, 2023 | JC | Aug 1-10, 2023 |
| Layton et al. [58] | TY | Aug 1-10, 2023 | JC | Aug 1-10, 2023 |
| Lavoie et al. [80] | TY | Aug 1-10, 2023 | JC | Aug 1-10, 2023 |
| Kwok et al. [81] | TY | Aug 1-10, 2023 | JC | Aug 1-10, 2023 |
| Kringle et al. [59] | TY | Aug 1-10, 2023 | JC | Aug 1-10, 2023 |
| Kortianou et al. [42] | TY | Aug 1-10, 2023 | JC | Aug 1-10, 2023 |
| Kim et al. [79] | TY | Aug 1-10, 2023 | JC | Aug 1-10, 2023 |
| Kikuchi et al. [31] | TY | Aug 1-10, 2023 | JC | Aug 1-10, 2023 |
| Khoury et al. [41] | TY | Aug 1-10, 2023 | JC | Aug 1-10, 2023 |
| Keteyian et al. [24] | TY | Aug 1-10, 2023 | JC | Aug 1-10, 2023 |
| Jarbandhan et al. [60] | TY | Aug 1-10, 2023 | JC | Aug 1-10, 2023 |
| James-Palmer et al. [82] | TY | Aug 1-10, 2023 | JC | Aug 1-10, 2023 |
| Hwang et al. [32] | TY | Aug 1-10, 2023 | JC | Aug 1-10, 2023 |
| Hume et al. [53] | TY | Aug 1-10, 2023 | JC | Aug 1-10, 2023 |
| Howroyd et al. [43] | TY | Aug 1-10, 2023 | JC | Aug 1-10, 2023 |
| Holland et al. [50] | TY | Aug 1-10, 2023 | JC | Aug 1-10, 2023 |
| Herkert et al. [40] | TY | Aug 1-10, 2023 | JC | Aug 1-10, 2023 |
| Held et al. [61] | TY | Aug 1-10, 2023 | JC | Aug 1-10, 2023 |
| Gehring et al. [77] | TY | Aug 1-10, 2023 | JC | Aug 1-10, 2023 |
| Galloway et al. [62] | TY | Aug 1-10, 2023 | JC | Aug 1-10, 2023 |
| Gagnon et al. [63] | TY | Aug 1-10, 2023 | JC | Aug 1-10, 2023 |
| Fioratti et al. [98] | TY | Aug 1-10, 2023 | JC | Aug 1-10, 2023 |
| Filakova et al. [78] | TY | Aug 1-10, 2023 | JC | Aug 1-10, 2023 |
| Farr et al. [88] | TY | Aug 1-10, 2023 | JC | Aug 1-10, 2023 |
| Fanget et al. [35] | TY | Aug 1-10, 2023 | JC | Aug 1-10, 2023 |
| van Egmond et al. [73] | TY | Aug 1-10, 2023 | JC | Aug 1-10, 2023 |
| Edwards et al. [64] | TY | Aug 1-10, 2023 | JC | Aug 1-10, 2023 |
| Donkers et al. [90] | TY | Aug 1-10, 2023 | JC | Aug 1-10, 2023 |
| Diamond et al. [54] | TY | Aug 1-10, 2023 | JC | Aug 1-10, 2023 |
| Dennett et al. [76] | TY | Aug 1-10, 2023 | JC | Aug 1-10, 2023 |
| Cox et al. [56] | TY | Aug 1-10, 2023 | JC | Aug 1-10, 2023 |
| Correia et al. [100] | TY | Aug 1-10, 2023 | JC | Aug 1-10, 2023 |
| Coronado et al. [96] | TY | Aug 1-10, 2023 | JC | Aug 1-10, 2023 |
| Cooley Hidecker et al. [83] | TY | Aug 1-10, 2023 | JC | Aug 1-10, 2023 |
| Colon-Semenza et al. [84] | TY | Aug 1-10, 2023 | DB | Aug 8-12, 2023 |
| Colas et al. [44] | TY | Aug 1-10, 2023 | DB | Aug 8-12, 2023 |
| Coats et al. [57] | TY | Aug 1-10, 2023 | DB | Aug 8-12, 2023 |
| Choi et al. [55] | TY | Aug 1-10, 2023 | DB | Aug 8-12, 2023 |
| Chen et al. [74] | TY | Aug 1-10, 2023 | DB | Aug 8-12, 2023 |
| Cerdan de Las Heras et al. [103] | TY | Aug 1-10, 2023 | DB | Aug 8-12, 2023 |
| Capin et al. [45] | TY | Aug 1-10, 2023 | DB | Aug 8-12, 2023 |
| Campbell et al. [94] | TY | Aug 1-10, 2023 | DB | Aug 8-12, 2023 |
| Brocki et al. [38] | TY | Aug 1-10, 2023 | DB | Aug 8-12, 2023 |
| Bianchini et al. [85] | TY | Aug 1-10, 2023 | DB | Aug 8-12, 2023 |
| Benvenuti et al. [69] | TY | Aug 1-10, 2023 | DB | Aug 8-12, 2023 |
| Batalik et al. [37] | TY | Aug 1-10, 2023 | DB | Aug 8-12, 2023 |
| Batalik et al. [25] | TY | Aug 1-10, 2023 | DB | Aug 8-12, 2023 |
| Ashikaga et al. [39] | TY | Aug 1-10, 2023 | DB | Aug 8-12, 2023 |
| Alwakeel et al. [51] | TY | Aug 1-10, 2023 | CL | Aug 5-10, 2023 |
| Ackerley et al. [95] | TY | Aug 1-10, 2023 | CL | Aug 5-10, 2023 |
| Wilson et al. [70] | TY | Aug 1-10, 2023 | CL | Aug 5-10, 2023 |
| van der Kolk et al. [86] | TY | Aug 1-10, 2023 | CL | Aug 5-10, 2023 |
| Van De Winckel et al. [65] | TY | Aug 1-10, 2023 | CL | Aug 5-10, 2023 |
| Song et al. [36] | TY | Aug 1-10, 2023 | CL | Aug 5-10, 2023 |
| Snoek et al. [26] | TY | Aug 1-10, 2023 | CL | Aug 5-10, 2023 |
| Simpson et al. [66] | TY | Aug 1-10, 2023 | AA | Aug 5-10, 2023 |
| Simpson et al. [46] | TY | Aug 1-10, 2023 | AA | Aug 5-10, 2023 |
| Sheehy et al. [93] | TY | Aug 1-10, 2023 | AA | Aug 5-10, 2023 |
| Seidler et al. [87] | TY | Aug 1-10, 2023 | AA | Aug 5-10, 2023 |
| Schlichting et al. [89] | TY | Aug 1-10, 2023 | AA | Aug 5-10, 2023 |
| Sari et al. [92] | TY | Aug 1-10, 2023 | AA | Aug 5-10, 2023 |
| Saitoh et al. [27] | TY | Aug 1-10, 2023 | AA | Aug 5-10, 2023 |
| Rosenbek Minet et al. [52] | TY | Aug 1-10, 2023 | AA | Aug 5-10, 2023 |
| Rosen et al. [47] | TY | Aug 1-10, 2023 | AA | Aug 5-10, 2023 |
| Qiu et al. [67] | TY | Aug 1-10, 2023 | AA | Aug 5-10, 2023 |
| Plaza et al. [97] | TY | Aug 1-10, 2023 | MG | Aug 5-10, 2023 |
| Piraux et al. [104] | TY | Aug 1-10, 2023 | MG | Aug 5-10, 2023 |
| Piraux et al. [72] | TY | Aug 1-10, 2023 | MG | Aug 5-10, 2023 |
| Piotrowicz et al. [28] | TY | Aug 1-10, 2023 | MG | Aug 5-10, 2023 |
| Piotrowicz et al. [33] | TY | Aug 1-10, 2023 | MG | Aug 5-10, 2023 |
| Pinto et al. [29] | TY | Aug 1-10, 2023 | MG | Aug 5-10, 2023 |
| Pfister et al. [99] | TY | Aug 1-10, 2023 | MG | Aug 5-10, 2023 |
| Peng et al. [34] | TY | Aug 1-10, 2023 | MG | Aug 5-10, 2023 |
| Paul et al. [91] | TY | Aug 1-10, 2023 | MG | Aug 5-10, 2023 |
| Parraguez et al. [75] | TY | Aug 1-10, 2023 | MG | Aug 5-10, 2023 |
| Palmcrantz et al. [68] | TY | Aug 1-10, 2023 | MG | Aug 5-10, 2023 |
| Ozturk et al. [102] | TY | Aug 1-10, 2023 | MG | Aug 5-10, 2023 |
| Øra et al. [71] | TY | Aug 1-10, 2023 | MG | Aug 5-10, 2023 |
| Moriichi et al. [101] | TY | Aug 1-10, 2023 | MG | Aug 5-10, 2023 |
| Mayer et al. [48] | TY | Aug 1-10, 2023 | MG | Aug 5-10, 2023 |
| Martin et al. [49] | TY | Aug 1-10, 2023 | MG | Aug 5-10, 2023 |
