# Supplementary material for: Adverse events associated with the delivery of telerehabilitation across rehabilitation populations: A scoping review
Source: PLoS One. 2024 Nov 19;19(11):e0313440. doi: 10.1371/journal.pone.0313440 (PMC11575805; doi:10.1371/journal.pone.0313440)
Supplement: S5 Appendix — (PDF) [file pone.0313440.s005.pdf]

Authors	Published Year, Pre/Post Covid. Sample Size, Mean age, Sex, Country of Origin, Population:	Study Design	Why: Rationale	What: Materials	Procedures: type of exercise/therapy e.g. aerobic, respiratory rehab, etc.	Who provided:<br><br>Where: where is the therapist located and where is the patient located	How: Full Telerehab or Hybrid (with in person component)<br><br>Synchronicity:Synchronous vs Asynchronous vs Hybrid	When and How much: How many times was the intervention delivered and over what period of time, including number of sessions, schedule, duration, intensity or dose	Tailoring: If the intervention was planned to be personalised, titrated or adapted<br><br>Modifications: If the intervention was modified during the course of the study<br><br>How well: Adherence	Outcome Measure Adverse events/participant, adverse event/sessions, severity, physical vs non-physical, related/unrelated, description
Lundgren et al. [30]	**Published Year:** 2023<br>**Pre-Covid**<br>**Sample Size:** intervention group = 31, control group = 30, total = 61<br>**Mean age:** 67.6<br>**Sex:** 25.8% women<br>**Country of origin:** Norway<br>**Population:** CHF patients from outpatient CR programmes	RCT	Evaluate the feasibility of a 3-month real-time, home-based telerehabilitation, high-intensity exercise programme for CHF patients who are either unable or unwilling to participate in standard outpatient cardiac rehabilitation and to explore outcomes of self-efficacy and physical fitness at 3 months post-intervention.	Tablet computer (Apple iPad Air2), online videoconferencing software used for live group-based exercises, 4G mobile network SIM card, pre-recorded videos with instructions for home-based self-administered exercise.	Cardiac rehab, real-time, home-based, high-intensity exercise intervention, via online videoconferencing software. High-intensity intervals, with exercises involving large muscle groups. Pre-recorded videos with instructions for self-administered exercise.	**Who provided:** Experienced physical therapist specialized in cardiac rehabilitation.<br><br>**Where:** Physical therapist from unknown location connecting virtually, patient in their own homes.	Full telerehab Synchronous Video conferencing	Twice a week for a period of 3 months or equivalent (24 exercise sessions). 60 minute sessions, starting with a 20 minute warm-up period, followed by 4 bouts of 4 min high-intensity intervals, with exercises involving large muscle groups, with intervals interspersed with 3 min recovery periods of lower intensity focusing on upper and lower body strength and balance, ending with 15 minute calm-down period.<br><br>Participants were also encouraged to perform additional exercise sessions to meet current guideline recommendations, and had access to pre-recorded exercise session videos for this.	**Tailoring**: Individual prescription varied to be equivalent to Borg scale above 15.<br>**Modifications**: Participants were given extra time to achieve the prescribed number of exercise sessions if their participation was prevented by illness or injury.<br>**Adherence**: 12 participants were adherent (38.7%), 13 participants were partially adherent (41.9%), and 6 participants were non-adherent (19.4%)	0 adverse events/31 participants<br>0 adverse events/744 sessions<br>Severity: N/A<br>Type: N/A<br>Relatedness: N/A<br>Description: N/A
Layton et al. [58]	**Published Year**: 2021<br>**Post-Covid**<br>**Sample Size:** intervention group = 11, control group = 8, total = 19<br>**Mean age**: 30<br>**Sex:** 46% women<br>**Country of origin:** USA<br>**Population:** Severe Cystic Fibrosis	Quasi	Pilot, a home-based pulmonary rehabilitation (PR) program administered via a telemedicine approach, using a combination of fitness application and self-selected activity, in lung transplant candidates with cystic fibrosis (CF).	Baseline spirometry, general self-efficacy scale, international physical activity questionnaire, six-minute walk test, full 12-lead electrocardiogram, pulse oximetry. Educational material and a personalized exercise plan based on Cardiopulmonary Exercise Test, live classes or library of workout videos via Peleton app, Bluetooth-enabled heart rate monitor, exercise log, pulse oximetry, Borg scale.	Pulmonary rehabilitation, live classes, or library of workout videos via Peleton app, consisting of cycling, treadmill walking, strength training, stretching, outdoor walking or running, dance videos, body weight exercises, and plyometric exercises.	**Who provided:** "Study team", authors are physicians.<br><br>**Where:** Physical therapist unknown location connecting virtually, patient in their own homes.	Full telerehab Asynchronous Text message, email	5/11 of the participants completed at least 24 sessions of home exercise in 12 weeks. On average, participants completed 19 sessions of home exercise in 12 weeks. On average participants completed 31 total workouts over an undefined number of weeks.	**Tailoring**: Personalized exercise plan based on Cardiopulmonary Exercise Test.<br>**Modifications**: One patient with pre-existing frequent hemoptysis was informed by doctor to take 2 weeks off from exercise, and received clearance before resuming without performing prone exercises.<br>**Adherence**: 5/11 of the participants completed at least 24 sessions of home exercise in 12 weeks. On average, participants completed 19 sessions of home exercise in 12 weeks. On average participants completed 31 total workouts over an undefined number of weeks.	13 adverse events/11 participants<br>13 adverse events/209 sessions<br>severity: non-serious<br>Type: physical<br>Relatedness: N/A<br>Description: muscle fatigue, cough/wheeze, SOB, headache after exercise, lightheadedness, chest discomfort due to acute infection, minor hemoptysis with pre-existing hemoptysis

Lavoie et al. [80]	**Published Year:** 2021<br>**Pre-Covid**<br>**Sample size:** total participants = 11<br>**Mean age:** 69.2<br>**Sex:** 45.5% women<br>**Country of origin:** Canada<br>**Population:** Parkinson's disease	Quasi	Document the feasibility of a physiotherapy telerehabilitation intervention for patients with PD and estimate the change over time in functional capacity, HRQOL, and the rate of falls.	TeraPlus (software platform for videoconferencing), pan-tilt-zoom cameras, hardware + system software, daily diary	PD rehab - functional tasks with the use of cognitive strategies; walking and balance exercises in different situations, on unstable surfaces, and in varied environments; with eyes closed and multiple direction changes; and flexibility exercises of the calf muscles and strengthening of the lower limb extensors and hip muscles. Initial information session on symptoms of PD and teaching cognitive strategies for locomotion.	**Who provided:** Physiotherapist<br><br>**Where:** Therapist located at Hôpital de jour gériatrique of the Centre intégré de santé et de services sociaux de Chaudière-Appalaches (CISSS-CA) in Lévis, Quebec. Participants in their own homes.	Full telerehab Hybrid (synchronous and asynchronous)<br><br>Video conferencing	8-week period, with two weekly sessions lasting 1 hour each. Videoconferencing sessions occurred twice a week, over an 8 week period. Participants also completed three weekly sessions of unsupervised exercises, each lasting 60 minutes. Filled out a daily diary, allowing the physiotherapist to follow up on progress and adjust exercises.	**Tailoring:** The exercises selected were tailored to each participant according to the impairments and limitations, exercises progressed according to the participant's tolerance and how well the participant had executed the task.<br>**Modifications:** N/A.<br>**Adherence**: Every participant completed each of the programme sessions (participation rate of 100%)	0 adverse events/11 participants<br>0 adverse events/440 sessions (176 supervised, 264 unsupervised)<br>Severity: N/A<br>Type: N/A<br>Relatedness: N/A<br>Description: N/A
Kwok et al. [81]	**Published Year:** 2022<br>**Post-Covid**<br>**Sample size:** total participants = 8<br>**Mean age:** 63.1<br>**Sex:** 50% women<br>**Country of origin:** Hong Kong<br>**Population:** Parkinson's disease	mixed methods	Evaluate the feasibility, safety, and preliminary effects of the mHealth-delivered home-based mindfulness yoga program on functional balance, motor symptoms, mental health and HRQOL in patients with PD.	Zoom for videoconferencing, webpage with guided videos, yoga mat and yoga block, sturdy chair, towel	Mindfulness yoga intervention, consisting of 10 min breathing techniques, 60 min yoga sequence, 15 min meditation., 5 min sharing and conclusion. Daily 15 min self-practice of mindful walking, guided by pre-recorded video.	**Who provided:** "study team", authors are nurses + physicians<br><br>**Where:** therapist unknown location connecting virtually, patient in their own homes.	full telerehab synchronous video conferencing, telephone	Biweekly 90 minute training sessions via Zoom, for 4 weeks. Daily 15 min self-practice of mindful walking, guided by pre-recorded video.	**Tailoring**: N/A<br>**Modifications**: N/A<br>**Adherence:** 98.4% adherence	0 adverse events/ 8 participants<br>0 adverse events/64 sessions<br>severity: N/A<br>type: N/A<br>relatedness: N/A<br>description: N/A
Kringle et al. [59]	**Publication Year:** 2020<br>Pre-Covid<br>**Sample size:** total participants = 5<br>**Mean age:** 68.2<br>**Sex:** 80% women<br>**Country of origin:** USA<br>**Population:** Chronic stroke	qualitative	adapt the delivery of strategy training for remote delivery using mobile health technology.	iADAPTS application, iADAPTS web-based clinician portal, user guide,	metacognitive rehabilitation approach strategy training (four step Goal-Plan-Do-Check) for any activity-based goal that the patient desires.	**Who provided:** occupational therapist<br><br>**Where:** therapist unknown location connecting virtually, patient any location of their choosing.	full telerehab asynchronous mobile application and telephone	5 week period, no intensity/duration/dose available.	**Tailoring:** personalized intervention that addresses patients' real life activity-based goals.<br>**Modifications:** N/A<br>**Adherence:** N/A	0 adverse events/5 participants<br>0 adverse events/34 sessions<br>severity: N/A<br>type: N/A<br>relatedness: N/A<br>description: N/A
Kortianou et al. [42]	**Publication Year:** 2022<br>**Post-Covid**<br>**Sample size**: total participants = 35<br>**Mean age:** 52.5<br>**Sex:** 27.1% women<br>**Country of origin:** Greece<br>**Population:** COVID-19 patients	quasi	assess the clinical effects of a home-based tele-rehabilitation exercise program following COVID-19 hospital discharge	International Physical Activity Questionnaire (IPAQ-Gr), the Hospital Anxiety and Depression Scale (HADS), the modified Medical Research Scale for dyspnea (mMRC) and the Short Form-36 generic questionnaire (SF-36),. Written information and a demonstration video for each test procedure (Short Physical Performance Battery (SPPB), the 60 sec sit to stand test (60secSTS)\ and the 3-min step test (3MST)). Smartphone Viber App for monitoring patients' measurements.<br>Google Form to send data to therapist	home-based program consisting of daily unsupervised self-practice exercise and one-hour individualized and supervised telerehabilitation exercise sessions every 10 days (3 sessions per month).<br>Strength training, balance training, stretching	**Who provided:** physiotherapist<br><br>**Where:** therapist unknown location connecting virtually, patient in their own homes.	full telerehab synchronous mobile application	2 month home-based rehab program.<br>Daily unsupervised self-practice exercise 30 minutes per exercise session, 5 times per week, low to moderate level (Borg 9–13). 1 hour individualized and supervised telerehabilitation exercise sessions every 10 days (3 sessions per month).<br><br>Same workout for both unsupervised and supervised sessions<br>5-10 min warm up of breathing exercises + dynamic movement exercises + balance training<br>15-20 min lower limb strengthening exercises and weight bearing training<br>5-10 min cooldown exercises, slow movements + stretching	**Tailoring:** individualized depending on their individual needs, interest, and abilities<br>**Modifications:** N/A<br>**Adherence:** Twenty-two (29.7%) (18 males) completed the 2-month exercise program. Patients completed a mean of 18 exercise sessions. 13 participants dropped out of the study after 3 weeks of participation due to personal reasons/lack of time for regular exercise.	0 adverse events/35 participants<br>0 adverse events/210 sessions<br>severity: N/A<br>type: N/A<br>relatedness: N/A<br>description: N/A

Kim et al. [79]	**Publication Year:** 2022<br>**Post-Covid**<br>**Sample size:** total participants = 8<br>**Mean age:** 10.125<br>**Sex:** 50% women<br>**Country of origin:** Korea<br>**Population:** Pediatric Cancer	quasi	determine the feasibility and benefits of a videoconferencing-based home exercise programme to improve health-related quality of life (HRQOL), posttraumatic growth and physical strength levels of paediatric cancer survivors during the coronavirus disease 2019	mat, soft ball, resistance band. step-by-step video of joint exercises was provided for all participants to follow at home	home-based programme of play combined with exercise. Single exercises in 12/16 sessions, and partner exercises in 4/16 sessions (with sibling or parent). step-by-step video of joint exercises was provided for all participants to follow at home (30 min per day for 5 day) during the remaining days of the week. Phone call/text every week to encourage use of step-by-step videos and check/resolve issues.	**Who provided**: authors (two nurses and one attending college of sport science) and a sports medicine expert (certified clinical exercise physiologist) via Zoom.<br><br>**Where:** therapist unknown location connecting virtually, patient in their own homes.	full telerehab hybrid (synchronous and asynchronous) video conferencing and telephone/text	8 weeks total. 60 minute Zoom sessions, twice a week, consisting of warm up, main part (40-50min) consisting of exercises aimed at improving basal fitness, power, muscle strength, cardiorespiratory endurance, flexibility, control, stabilisation and compound exercises, and cooldown. All participants performed the step-by-step exercises for 30 minutes, 5 days per week for the 8 weeks.	**Tailoring:** The amount of exercise was adjusted depending on each participant's state and needs, in consideration of the child's mood, emotions and physical status<br>**Modifications:** N/A<br>**Adherence:** 1 patient dropped out of the study. Average attendance for those who completed the intervention was 98.4%, with a minimum of 93.8% (15/16 sessions attended) and a maximum of 100% (16/16 sessions attended).	0 adverse events/8 participants<br>0 adverse events/128 synchronous sessions<br>0 adverse events/320 asynchronous sessions<br>severity: N/A<br>type: N/A<br>relatedness: N/A<br>description: N/A
Kikuchi et al. [31]	**Publication Year:** 2021<br>**Post-Covid**<br>**Sample size:** total participants = 10<br>**Mean age**: 76<br>**Sex:** 40% women<br>**Country of origin:** Japan<br>**Population:** Heart Failure	quasi	evaluated the feasibility and safety of a new remote real-time monitoring system for supervising home-based CR among elderly patients with heart failure (HF) .	an internet of things (IoT)-equipped ergometer (Charimo®, Remohab. Inc.), an android-compatible tablet (TAB3-X70L®, LENOVO, Hong-Kong), and a wireless electrocardiographic monitoring device (hitoe®, TORAY, Tokyo, Japan). The platform was loaned to the patients along with an equipment manual containing written and pictorial instructions.	real-time telerehabilitation platform, aerobic exercise using IoT-equipped stationary ergometer. Patients took their own blood pressure before and after exercise, and entered it into the platform. Patient's physiological data [heart rate, electrocardiogram (ECG)] were streamed to a web server via Wi-Fi and displayed on the screen at the medical site.	**Who provided:** Nurse, attending physician<br><br>**Where:** therapist unknown location connecting virtually, patient in their own homes.	full telerehab synchronous video conferencing	12 weeks total, 3 sessions per week, Session duration was targeted at 30 minutes but some patients were permitted a shorter duration in line with their lower exercise tolerance.	**Tailoring:** Exercise intensity based on individual baseline cardio-pulmonary exercise test (CPET).<br>**Modifications:** Some patients were permitted a shorter duration in line with their lower exercise tolerance.<br>**Adherence:** All 10 patients completed the program without withdrawal during the study period. Median participation rate in the exercise sessions was 94.4%	9 adverse events/10 participants<br>9 adverse events/360 sessions<br>severity: non-serious<br>type: physical<br>relatedness: related, unrelated<br>description: fatigue, palpitations, bradycardia, common cold
Khoury et al. [41]	**Publication Year**: 2020<br>**Pre-Covid**<br>**Sample size**: total participants = 10<br>**Mean age:** 11.5<br>**Sex:** 70% women<br>**Country of origin:** Canada<br>**Population:** pediatric Fontan	quasi	determine the feasibility and safety of a new home-based high-intensity interval training programme in youth with Fontan physiology	custom paediatric remote cycle ergometer (MedBIKE™) linked to a video game platform, live two-way audiovisual feed, providing face-to-face communication, as well as electrocardiogram and pulse oximetry monitoring, and build-in tablet. User manual.	baseline pulmonary function test and cardiopulmonary exercise test assessment using a 10-W per minute ramp protocol. A baseline regimen was designed based on the results of the baseline exercise test, consisting of a 5-minute warm-up, seven 1-minute high-intensity intervals at 70–90% of peak power output with 1-minute breaks at 40–50% peak power output in-between, followed by a 5-minute cool-down.	**Who provided:** physician<br><br>**Where:** the therapist in a separate adjacent room, all communication via telemedicine system	full telerehab synchronous video conferencing via tablet on ergometer	One session. 5-minute warm-up, seven 1-minute high-intensity intervals at 70–90% of peak power output with 1-minute breaks at 40–50% peak power output in-between, followed by a 5-minute cool-down	**Tailoring**: Individual baseline regimen was designed based on the results of the baseline exercise test.<br>**Modifications:** N/A<br>**Adherence**: All 10 completed the session.	0 adverse events/10 participants<br>0 adverse events/10 sessions.<br>severity: N/A<br>type: N/A<br>relatedness: N/A<br>description: N/A
Keteyian et al. [24]	**Publication Year:** 2021<br>**Post-Covid**<br>**Sample size:** intervention group = 26, control group = 21, total participants = 47<br>**Mean age:** 63<br>**Sex:** 35% women<br>**Country of origin:** USA<br>**Population:** Cardiac	RCT	compare exercise training intensity during standard cardiac rehabilitation (S-CR) versus Hybrid-CR (combined clinic- and remote home-/community-based).	electrocardiogram (ECG) telemetry and before and after exercise blood pressure and blood glucose monitoring for at least the first three sessions.	≥ 30 min/session 3 days/week, using aerobic-type exercise equipment. Hybrid-CR TH visits are approximately 20 min in duration; CR staff connect with the patient during their warm-up period and then remain in contact for 16-18 min of the 30-min aerobic portion of their exercise session.	**Who provided:** cardiac rehabilitation staff<br><br>**Where**: for in-clinic CR visits: both patient and therapist in clinic telerehab sessions: patient at home or community, therapist unknown location	hybrid telerehab synchronous video conferencing	patients completed ≥ 3 in-clinic CR visits and up to 33 additional visits via a video-application (app) that is loaded to the patient's smart device. ≥ 30 min/session 3 d/wk. The study was conducted over a year.	**Tailoring:** exercise target range set at 60 - 80% using individual HR reserve, self-titrate exercise intensity using ratings of perceived exertion<br>**Modifications:** N/A<br>**Adherence:** the overall percentage of patients that trained within their prescribed THRR during each session was 91%. The overall percentage of patients that completed ≥ 30 min during a session was 93 ± 6%.	1adverse event/26 participants<br>1 adverse event/244 sessions<br>severity: non-serious<br>type: physical<br>relatedness: related<br>description: fall

Jarbandhan et al. [60]	**Publication Year:** 2022<br>**Post-Covid**<br>**Sample size:** intervention group = 20, control group = 10, total participants = 30<br>**Mean age:** 61.8<br>**Sex:** 56.66% women<br>**Country of origin:** Suriname<br>**Population:** Chronic stroke	RCT	assess feasibility and preliminary effectiveness of a home-based semi-supervised physiotherapy intervention to promote post-stroke mobility in a low resource setting.	pedometer, Garmin watch measuring resting BP, hand dynamometer with an adjusted handle, questionnaire	home-based, semi-supervised physiotherapy program. 10-15 min starting up - mobility exercises, 40-45 mins lower limb strengthening endurance exercise, 10-15 mins ending program: upper limb rehab, patient and family education, measuring resting blood pressure. Week 1-4 supervised in person, week 4-8 asynchronously supervised via phone calls	**Who provided:** physiotherapist<br><br>**Where:** patient at home, therapist with patient during week 1-4, therapist unknown location week 4-8 (not patient's home)	hybrid asynchronous phone call	3 times/week, 70 minutes/session, for 4 weeks.	**Tailoring:** individually tailored program by physiotherapist<br>**Modifications:** N/A<br>**Adherence:** Fourteen of the twenty (70%) participants completed the full intervention.	0 adverse events/20 participants<br>0 adverse events/240 sessions<br>severity: N/A<br>type: N/A<br>relatedness: N/A<br>description: N/A
James-Palmer et al. [82]	**Publication Year:** 2022<br>**Post-Covid**<br>**Sample Size:** total participants = 16<br>**Mean age:** 63.125<br>**Sex:** 62.5% women<br>**Country of origin:** USA<br>**Population:** Parkinson's disease	quasi	assess the safety and feasibility of a synchronous tele-yoga intervention for persons with PD.	Zoom videoconferencing platform,	yoga intervention was delivered one-on-one, remotely, and synchronously through the videoconferencing platform Zoom. (1) breathing exercises (first five to seven minutes), (2) yoga postures (middle 14 to 20 minutes), and (3) relaxation exercises (last five to seven minutes).	**Who provided:** physiotherapists<br><br>**Where**: therapist unknown location connecting virtually, patient in their own homes.	full telerehab synchronous video conferencing	The intervention dose of 30 minutes twice weekly for six-weeks	**Tailoring:** Sessions were individualized to fit the participants' needs<br>**Modifications:** N/A<br>**Adherence:** Adherence to assessment sessions was 100%. Yoga intervention adherence was 97%. Mean tele-yoga session attendance at 11.13 ± 2.10 sessions out of the 12 intended sessions.	37 adverse events/16 participants<br>37 adverse events/192 sessions<br>severity: 29 mild, 6 moderate, 2 severe<br>type: physical<br>relatedness: 16 not related, 5 unlikely related, 6 possible related, 2 probable related, 2 definite related, 6 not related<br>description: baseline pain, new pain, neuropathy, loss of balance, not feeling well, medication side effect, temporary pain/discomfort, increased awareness of hand tremors, temporary bout of dizziness, fall, ER visit, insomnia
Hwang et al. [32]	**Publication Year:** 2017<br>**Pre-Covid**<br>**Sample Size:** intervention group = 24, control group = 29, total participants = 53<br>**Mean age:** 68<br>**Sex:** 21% women<br>**Country of origin:** Australia<br>**Population:** Chronic heart failure	RCT	determine the efficacy and safety of a short-term, real-time, group-based heart failure rehabilitation program delivered into each participant's home via an online telerehabilitation system.	laptop computer, a mobile broadband device connected to 3G wireless broadband internet, an automatic sphygmomanometer, a finger pulse oximeter, free weights and resistance bands. equipment manual with written and pictorial instructions. Educational topics were delivered as electronic slide presentations.	warm-up, aerobic and strength exercises, cool-down	**Who provided:** physiotherapists<br><br>**Where:** therapist unknown location connecting virtually, patient in their own homes.	full telerehab synchronous video conferencing	group-based program, 60 minutes of exercise per session, two sessions per week. 24 week study. Each session consisted of a 10-minute warm-up, 40-minutes of aerobic and strength exercises, and a 10-minute cool-down. Exercise intensity commenced at 9 (very light) and gradually progressed towards 13 (somewhat hard) on the rate of perceived exertion scale. On average, patients in the experimental group completed 20 sessions.	**Tailoring:** Prescription was tailored to the participant's goal and the treating physiotherapist continuously reviewed it to ensure appropriate progression.<br>**Modifications:** N/A<br>**Adherence:** 17 participants (71%) were adherent (>80% sessions attended), 7 participants (29%) were partially adherent (20-80% sessions attended)	6 adverse events/24 participants<br>6 adverse events/480 sessions<br>severity: minor<br>type: physical<br>relatedness: N/A<br>description: angina, diaphoresis, palpitations

Hume et al. [53]	**Publication Year**: 2022<br>**Post-Covid**<br>**Sample Size**: intervention group = 7, control group = 5, total participants = 12<br>**Mean age:** 57<br>**Sex:** 42.85% women<br>**Country of origin:** UK<br>**Population:** lung transplant	RCT	This study assessed the feasibility and acceptability of a novel, 12-weeks physical activity tele-coaching (TC) intervention in LTx recipients.	pedometer, smartphone app, Linkcare web-based platform, home exercise booklet, telephone support	walking	**Who provided:** researchers from "Department of Sport, Exercise and Rehabilitation, Faculty of Health & Life Sciences, Northumbria University"<br><br>**Where**: therapist unknown location connecting virtually, patient in their own homes and community	full telerehab asynchronous mobile application and telephone	12 week intervention. Daily steps.<br><br>Each week an activity goal was set by the app, based on the patient's physical activity levels (steps/day) in the previous week. The goals were calculated using the mean and median of the 4 most active days. If the mean value exceeded the weekly goal, the application displayed the option to increase their median goal by 500 steps/day or to keep it the same as the previous week. If the mean value was lower than the weekly goal and the median was more than 500 steps/day below the goal, the goal was reduced to the median of the 4 most active days +500 steps/day. Otherwise, the goal remained the same.	**Tailoring:** Activity goal was set by the app, based on the patient's physical activity levels (steps/day) in the previous week.<br>**Modifications:** N/A<br>**Adherence:** 100% wore the pedometer for more than 90% of days over the 12-weeks intervention. The number of weekly step goal targets met throughout the 12-weeks intervention was good, with a mean (SD) of 82% of step goals achieved.	0 adverse events/7 participants<br>0 adverse events/588 "sessions"<br>severity: N/A<br>type: N/A<br>relatedness: N/A<br>description: N/A
Howroyd et al. [43]	**Publication Year:** 2023<br>**Post-Covid**<br>**Sample size:** total participants = 28<br>**Mean age:** 54<br>**Sex:** 21% women<br>**Country of origin:** England<br>**Population:** COVID-19	quasi	This service evaluation aimed to assess the safety, utilisation (recruitment, retention, and adherence), and potential impact on the recovery from physical and neuropsychiatric sequelae of an innovative virtual post-intensive-care rehabilitation programme for survivors of COVID-19	Patient's own smartphone, tablet device, laptop, or computer, using Microsoft Teams. Online patient information booklet was provided to each participant, including written instructions and photographs of each exercise, in addition to the Borg breathlessness scale	once-weekly exercise class followed by a support group, completed virtually via video call using Microsoft Teams.	**Who provided:** critical care physiotherapists and nurses<br><br>**Where:** therapist unknown location connecting virtually, patient in their own homes.	full telerehab synchronous video conferencing	8 week intervention, once-weekly exercise class followed by a support group. Structured, interval exercise approach, including warm-up, 20 minutes of circuit-based exercises, and a cool-down. Patients were advised to titrate exercise intensity based on their perceived breathlessness, aiming for a score of 3-4 on the modified Borg breathlessness scale	**Tailoring:** Patients were advised to titrate exercise intensity based on their perceived breathlessness, aiming for a score of 3-4 on the modified Borg breathlessness scale<br>**Modifications:** N/A<br>**Adherence:** 28 (74%) completed the rehabilitation programme. Of those that completed, 82% adhered (n = 23); quantified as attending 75% of planned rehabilitation sessions	0 adverse events/28 participants<br>0 adverse events/224 sessions<br>severity: N/A<br>type: N/A<br>relatedness: N/A<br>description: N/A
Holland et al. [50]	**Publication Year**: 2013<br>**Pre-Covid**<br>**Sample Size:** total participants = 8<br>**Mean age**: 66<br>**Sex:** 62.5% women<br>**Country of origin:** Australia<br>**Population**: COPD	quasi	establish the feasibility and acceptability of real time, home-based telerehabilitation for people with COPD using existing technology	exercise bicycle, a tablet computer with webcam for low bandwidth videoconferencing, and a pulse oximeter positioned so that the display was visible while videoconferencing	aerobic training pulmonary rehab	**Who provided:** physiotherapist<br><br>**Where:** therapist in hospital/university, connecting virtually, patient in their own homes	full telerehab synchronous video conferencing	supervised aerobic training twice a week for eight weeks.<br>The exercise programme involved cycling at an intensity of 60% of peak work estimated from the initial 6MWT. Duration was increased up to 30 min, and then intensity was increased according to standardised criteria. Participants were encouraged to exercise at a dyspnoea score of 3 (moderate dyspnoea). The intensity of exercise was reduced or stopped if SpO2 dropped below 88%, heart rate rose above 150 beats per minute, dyspnoea scores were greater than 4 (somewhat severe) or participants exhibited signs of discomfort or distress.	**Tailoring:** intensity estimated from individual initial 6MWT. The intensity of exercise was reduced or stopped if SpO2 dropped below 88%, heart rate rose above 150 beats per minute, dyspnoea scores were greater than 4 (somewhat severe) or participants exhibited signs of discomfort or distress.<br>**Modifications:** N/A<br>**Adherence:** Participants attended 76% of possible sessions.	7 adverse events/128 sessions<br>7 adverse events/8 participants<br>severity: minor<br>type: physical<br>relatedness: N/A<br>description: desaturation to less than 88%, heart rate >150bpm

Herkert et al. [40]	**Publication Year**: 2021<br>**Post-Covid**<br>**Sample Size**: total participants = 10<br>**Mean age:** 71<br>**Sex**: 50% women<br>**Country of origin:** Netherlands<br>**Population**: advanced combined cardiopulmonary disease	quasi	assess the feasibility of a personalized, home-based, goal-oriented exercise program in patients with advanced, combined chronic cardiac and pulmonary diseases.	secure digital platform (Mibida BV). wrist-worn activity tracker. An activity diary. Exercises for (respiratory) muscle strengthening, breathing techniques, and techniques for mobilization of sputum were provided through instruction videos on the digital platform. activity diary to be filled in by the participant, which could be rated afterward by the occupational therapist.	8-week home-based exercise program consisted of a combination of endurance and strength exercise training tailored to the participants' preferences in exercise modality (ie, walking, cycling, or swimming) and availability of training equipment at home.	**Who provided:** physiotherapist and occupational therapist<br><br>**Where:** therapist unknown location connecting virtually, patient in their own homes.	full telerehab synchronous video conferencing	8 weeks, the amount, duration, and content of the exercise sessions was determined individually by the physiotherapist	**Tailoring:** The amount, duration, and content of the exercise sessions was determined individually by the physiotherapist.<br>**Modifications:** N/A<br>**Adherence:** 90% completed the 8-week exercise program, whereas 1 (10%) participant ended the program prematurely. The median adherence over 8 weeks was 75%	0 adverse events/10 participants<br>severity: N/A<br>type: N/A<br>relatedness: N/A<br>description: N/A
Held et al. [61]	**Publication Year:** 2018<br>**Pre-Covid**<br>**Sample Size:** total participants = 15<br>**Mean age:** 56<br>**Sex:** 55.56% women<br>**Country of origin:** Switzerland and Spain<br>**Population:** stroke	quasi	study the safety, usability, and patient acceptance of an autonomous telerehab system for balance and gait in patients' home	REWIRE autonomous telerehabilitation platform, computer, TV screen, force plate, 3D camera, Kinect camera,	VR exergames, composed of a mix of exercises that often involve equilibrium, coordination, and increase of lower limbs muscle strength	**Who provided:** therapist<br><br>**Where:** therapist in hospital, patient in their own homes	full telerehab asynchronous VR, computer REWIRE platform patient station logs gaming data and gets sent to hospital station for assessment by clinician	12 weeks, 10-40 mins per day, depending on patients' capacity and the therapist knowledge of performing each exercise.	**Tailoring:** the therapist tailored exercises to the patient status choosing the adequate level of difficulty.<br>**Modifications:** N/A<br>**Adherence:** On average, patients completed 71% of the scheduled sessions.	0 adverse events/15 participants<br>0 adverse events/1260 sessions<br>severity: N/A<br>type: N/A<br>relatedness: N/A<br>description: N/A
Gehring et al. [77]	**Publication Year**: 2018<br>**Pre-Covid**<br>**Sample Size:** intervention group = 21, control group = 11, total participants = 32<br>**Mean age:** 48<br>**Sex:** 56% women<br>**Country of origin:** Netherlands<br>**Population:** glioma	RCT	investigated the feasibility of a home-based, remotely guided exercise intervention for patients with gliomas.	Heart rate monitor. log of their training experiences. Online platform	aerobic training.	**Who provided:** physiotherapist<br><br>**Where:** therapist unknown location, patient in their own homes.	full telerehab asynchronous email/phone	three home-based aerobic training sessions per week, for a duration of six months. At the start of the intervention, a physiotherapist visited participants at home. Patients received an individualized exercise prescription, based on their level of aerobic fitness, to exercise at 60–85% of their maximum heart rate. The physiotherapist monitored the training data on the platform on a weekly basis and provided additional personal feedback by e-mail	**Tailoring:** individualized exercise prescription, they could choose one or more central activities, as long as these could meet the prescribed exercise intensity<br>**Modifications:** N/A<br>**Adherence:** participants in the exercise group adhered to 79% of the prescribed sessions (i.e., a mean of 2.4 sessions and an average training time of 126 minutes per week)	0 adverse events/32 participants<br>0 adverse events/2496 sessions<br>severity: N/A<br>type: N/A<br>relatedness: N/A<br>description: N/A
Galloway et al. [62]	**Publication Year:** 2019<br>**Pre-Covid**<br>**Sample Size:** total participants = 21<br>**Mean age:** 62.4<br>**Sex:** 43% women<br>**Country of origin:** Australia<br>**Population:** Stroke	mixed methods	To assess the feasibility of, and level of satisfaction with home based telehealth supervised aerobic exercise training post stroke.	Telehealth video-conference sessions were delivered using proprietary software, via a website (www.neorehab.com) or app (eHab®). Laptop or iPad If video-conferencing was not available due to technical issues, sessions were to be conducted by phone. pulse oximeter, heart rate monitor incorporating a chest strap and wrist watch.	aerobic exercise	**Who provided:** physiotherapist or exercise scientist<br><br>**Where:** therapist unknown location, patient in their own homes.	hybrid telerehab synchronous video conferencing	8-week, 3d/week home-based, individually prescribed, aerobic exercise program at moderate to vigorous intensity (55-85% of maximum heart rate as determined at the baseline fitness assessment, or at a Borg rating of perceived exertion [RPE] between 13 and 16)	**Tailoring:** Exercise selection was adapted to patient ability<br>**Modifications:** If participants were unable to perform the proposed exercises, these exercises were modified or alternative exercises identified. If mutually convenient times were not found, participants were encouraged to complete sessions unsupervised.<br>Adherence: There were 504 scheduled exercise sessions; 476 (94%) exercise sessions were completed and 408 (85% of scheduled) sessions were completed via telehealth (by n=20 participants).	1 adverse event/20 participants<br>1 adverse event/408 sessions<br>severity: N/A<br>type: physical<br>relatedness: related<br>description: fall

Gagnon et al. [63]	**Publication Year:** 2023<br>**Post-Covid**<br>**Sample size:** total participants = 8<br>**Mean age:** 60<br>**Sex:** 67% women<br>**Country of origin:** Canada<br>**Population:** stroke	quasi	The overall aim of this study was to evaluate the feasibility of FAME@home for eHealth delivery.	home based group eHealth program called Fitness and Mobility Exercise FAME@home, Zoom, tablets, exercise elastic band	1) warm-up, 2) balance, functional strength, and fitness activities, 3) cooldown stretch.	**Who provided:** kinesiologist<br><br>**Where:** therapist unknown location, patient in their own homes.	full telerehab synchronous video conferencing	two 1-hour sessions per week for 12 weeks	**Tailoring:** N/A<br>**Modifications:** N/A<br>**Adherence:** 75% were adherent (defined as % of participants who attend 22/24 training sessions)	0 adverse events/8 participants<br>0 adverse events/192 sessions<br>severity: N/A<br>type: N/A<br>relatedness: N/A<br>description: N/A
Fioratti et al. [98]	**Publication Year:** 2022<br>**Post-Covid**<br>**Sample Size:** intervention group = 31, control group = 33, total participants = 64<br>**Mean age**: 40.2<br>**Sex:** 61% women<br>**Country of origin:** Brazil<br>**Population**: Chronic MSK pain	RCT	study aims to evaluate the feasibility, usability, and implementation context of a self-management internet-based program based on exercises and pain education (ReabilitaDOR) in people with chronic musculoskeletal pain and to compare this program with a program using only a web-based self-management booklet.	web-based platforms and telephone calls. website developed for the study, videos and animations based on pain education, promotion of physical activity, and general exercises.	general exercises with the aim of improving strength, flexibility, control, and coordination.	**Who provided:** physiotherapist<br><br>**Where:** therapist unknown location, patient in their own homes.	full telerehab asynchronous text message, phone call	8 weeks. There was new content every week of the intervention, and the patients were instructed to perform the video exercises at least 3 times a week and watch the videos as necessary.	**Tailoring:** N/A<br>**Modifications:** N/A<br>**Adherence:** Adherence to the 8-week program (telerehabilitation group) was high, with a mean of 7.2 (SD 1.4) intervention weeks accessed by the patients on the program website.	0 adverse events/31 participants<br>0 adverse events/744 sessions<br>severity: N/A<br>type: N/A<br>relatedness: N/A<br>description: N/A
Filakova et al. [78]	**Publication Year:** 2023<br>**Post-Covid**<br>**Sample size:** total participants = 11<br>**Mean age:** 60.3<br>**Sex:** 73% women<br>**Country of origin:** Czech Republic<br>**Population:** hematological cancer	quasi	to evaluate the feasibility, safety, and effect of a 12-week home-based CORE intervention in telerehabilitation approach among hematological cancer survivors.	a HR sensor, a web platform compatible with the sensor, and telesupervising via telephone call, web-based training diary, personal questionnaire (sex, age, diagnosis, and pharmacological treatment), a trial manual, and an educational booklet (nutrition advice, obesity management, diabetes mellitus management, smoking cessation, strength, and flexibility exercise examples).	cardio-oncology rehabilitation. Modality of walking, Nordic walking, or cycling was recommended.	**Who provided:** physiotherapist<br><br>**Where:** therapist unknown location, patient in their own homes.	full telerehab asynchronous web platform, telephone call	was 12 weeks of home-based CORE (3 sessions per week). The HR training zone was determined based on baseline CPET (60–85% HRmax) and Rating of Perceived Exertion (11–13 degrees on a 1–20 scale) The training modality was determined according to the participant´s preference. Modality of walking, Nordic walking, or cycling was recommended.	**Tailoring:** The HR training zone was determined based on baseline CPET (60–85% HRmax) and Rating of Perceived Exertion (11–13 degrees on a 1–20 scale)<br>**Modifications:** N/A<br>**Adherence:** The rate of participation in the planned training sessions was 78.2%. On average, the participant completed 30.5±6.8 sessions (range: 13–36).	0 adverse events/11 participants<br>0 adverse events/396 sessions<br>severity: N/A<br>type: N/A<br>relatedness: N/A<br>description: N/A
Farr et al. [88]	**Publication Year**: 2021<br>**Pre- Covid**<br>**Sample Size:** intervention group = 10, control group = 11, total participants = 21<br>**Mean age:** no mean provided; 27% secondary school - 11-16 years old, 73% primary school - 5-11 years old<br>**Sex:** 20% women<br>**Country of origin:** England<br>**Population:** cerebral palsy	RCT	We assessed the feasibility of a virtual reality therapy mode of intervention, appropriateness of measures, and potential cost-effectiveness.	diary, Nintendo Wii Fit	Nintendo Wii Fit	**Who provided:** physiotherapist<br><br>**Where:** therapist unknown location, patient in their own homes.	full telerehab asynchronous phone call as communication (VR study)	30 min, 3 times per week for 12 weeks, and asked to keep a diary of their activity.	**Tailoring:** individualised activity programme<br>**Modifications:** N/A<br>**Adherence:** Completed a mean number of 19/36 sessions (56% adherence)	0 adverse events/10 participants<br>0 adverse events/360 sessions<br>severity: N/A<br>type: N/A<br>relatedness: N/A<br>description: N/A

Fanget et al. [35]	**Publication Year**: 2022<br>**Post-Covid**<br>**Sample Size**: intervention group = 27, control group = 27, total participants = 54<br>**Mean age**: 63.3<br>**Sex**: 14.8% women<br>**Country of origin**: France<br>**Population:** coronary artery disease	quasi	investigate and measure the effects of home-based CR compared to conventional center-based CR on cardiorespiratory functions in coronary artery disease patients.	HR and steps watch, computer or tablet, cycle ergometer, therapeutic education meeting on their disease, nutrition, and physical activity	cardiac rehabilitation	**Who provided:** adapted physical activity coach<br><br>**Where:** therapist unknown location, patient in their own homes.	full telerehab synchronous tablet/computer, video conferencing	four consecutive sessions of physical activity per week, for 3 weeks. The training session consisted of 30 min of cycling and 20 min of strength training. warm-up period, a CV training period, and a cool down phase. In addition, an aerobic interval training was gradually offered as an alternative to continuous endurance exercise. The dynamic resistance training consisted of overall muscle strength training or focused more on the lower or upper limbs.	**Tailoring:** exercises adapted accordingly to individual CPET<br>**Modifications:** N/A<br>**Adherence:** all participants completed the exercise intervention.	0 adverse events/27 participants<br>0 adverse events/324 sessions<br>severity: N/A<br>type: N/A<br>relatedness: N/A<br>description: N/A
van Egmond et al. [73]	**Publication Year:** 2020<br>**Pre-Covid**<br>**Sample Size:** intervention group = 15, control group = 30, total participants = 45<br>**Mean age:** 64.6<br>**Sex:** 23% women<br>**Country of origin:** Netherlands<br>**Population**: esophageal cancer	mixed methods	investigate the feasibility of a 12-week supervised postoperative physiotherapy intervention with telerehabilitation for patients with esophageal cancer who underwent esophagectomy and had postoperative complications or who had an increased LoS.	Physitrack - an eHealth platform, in weekly telephone, email, or video sessions,	aimed at improving functional status, increasing muscle strength, coordination, range of joint motion, stamina. Intensity and frequency based on ACSM guidelines. Cardiorespiratory exercises to improve stamina and exercises to improve muscle strength	**Who provided:** physiotherapist<br><br>**Where:** therapist unknown location, patient in their own homes.	full telerehab asynchronous eHealth platform, telephone, email, or video sessions	12 weeks with at least two sessions per week, depending on whether the treatment goals were achieved. Cardiorespiratory exercises to improve stamina were performed on a moderate-to-vigorous intensity level, measured using the Borg rating of 13-16. Exercises to improve muscle strength were performed 2 to 3 days per week on 60% to 70% of the 1 repetition maximum (moderate-to-hard intensity).	**Tailoring**: The exercises were tailored to the patients' specific condition and needs, which were determined a day before T0.<br>**Modifications:** N/A<br>**Adherence:** 15 patients completed the intervention. Patient adherence was 99.8% in the first 6 weeks and dropped to 75.6% in the following 6 weeks,	0 adverse events/15 participants<br>0 adverse events/360 sessions<br>severity: N/A<br>type: N/A<br>relatedness: N/A<br>description: N/A
Edwards et al. [64]	**Publication Year:** 2023<br>**Post-Covid**<br>**Sample Size:** total participants = 16<br>**Mean age:** 61.26<br>**Sex:** 37.5% women<br>**Country of origin:** USA<br>**Population:** stroke	quasi	To examine the feasibility, safety, and potential efficacy of an established telerehabilitation (TR) program after stroke initiated during admission to an inpatient rehabilitation facility (IRF) and completed in the patient's home.	web-based therapist portal, videoconferencing, TR device with console, controllers, fish-eye lens. The TR device includes a range of controllers: to focus on gross shoulder and elbow movements, the use of large stationary buttons in the table-top console, or wireless hand-held controllers may be suitable. For fine movement control, console-based options include a trackpad, a dial, a pinch gage, and small buttons. Weighted dowel, resistance bands, hand gripper, PVC pipe, and/or squeeze balls for adding resistance. ball foam, hand trainer, dice or blocks to simulate components for functional tasks.	stroke rehab. Organ functions: adaptive exercise training, stretching, skills and habits: repetitive unilateral arm motor functional practice, representations: stroke education, functional goals, progressive load of cognitive demand	**Who provided:** licensed therapist<br><br>**Where:** therapist at UCLA and MossRehab, patient in their own home	full telerehab synchronous web-based therapist portal, video conferencing	Treatment consisted of 36, 70-minute sessions<br>(half supervised by a licensed therapist via videoconference), over a 6-week period, that included functional games, exercise videos, education, and daily assessments. Sessions began with a daily assessment of 4 behaviors: proximal UE movement, via a targeting reaching game by the paretic UE; distal UE movement, assessing maximum tapping speed of the paretic index finger at the metacarpophalangeal joint over 10 taps; general fatigue, using a visual analog scale (VAS); and shoulder pain on the paretic side using a VAS. Each session then involved at least 15minutes of exercises, at least 15minutes of functional games, and 5minutes of stroke education	**Tailoring:** the treatment therapist examined the patient before the first TR therapy session and then created a personalized 70-minute session. Adjusted the duration and difficulty of each, as appropriate for each patient's therapeutic goals.<br>**Modifications:** N/A<br>**Adherence:** Compliance was 100%, Sixteen participants of 19 allocated completed the intervention	6 adverse events/16 participants<br>6 adverse events/288 supervised sessions<br>severity: 2 not serious, 4 serious<br>type: physical, non-physical<br>relatedness: 2 possibly related, 4 unrelated<br>description: nausea, demotivation when filling out anxiety and depression assessment, sepsis, pneumonia, recurrent stroke, shoulder injury outside of study
Donkers et al. [90]	**Publication Year:** 2020<br>**Pre-Covid**<br>**Sample Size:** intervention group = 32, control group = 16, total participants = 48<br>**Mean age:** 54.6<br>**Sex:** 63% women<br>**Country of origin:** Canada<br>**Population:** moderate-to-severe multiple sclerosis	RCT	to evaluate adherence to a Web-based, individualized exercise pro-<br>gram in moderate-to-severe MS.	web-based intervention webbasedphysio.com (now www.giraffehealth.com), online exercise diary.	MS rehab. developed based on input from people with MS in the UK with mild-to-moderate disability, adapted by a physiatrist and four experienced physiotherapists, including seated versions of exercises and novel exercises focused on core and upper extremity strength	**Who provided:** physiotherapist<br><br>**Where:** therapist unknown location, patient in their own homes.	full telerehab asynchronous web-based intervention, online exercise diary	A minimum of twice-per-week exercise sessions for 6 months was prescribed for all the participants (2 × 26 weeks = 52 exercise diary entries).	**Tailoring:** individually prescribed by a physiotherapist at an initial assessment<br>**Modifications:** N/A<br>**Adherence:** Average adherence in the Web-based group was 38.9 sessions/52 sessions (77.8%)	0 adverse events/32 participants<br>0 adverse events/1664 sessions<br>severity: N/A<br>type: N/A<br>relatedness: N/A<br>description: N/A

Diamond et al. [54]	**Publication Year:** 2021<br>**Post-Covid**<br>**Sample Size**: total participants = 18<br>**Mean age**: 54.3<br>**Sex:** 72.2% women<br>**Country of origin:** USA<br>**Population:** lung transplant	mixed methods	Evaluate the feasibility, safety, and efficacy of a mHealth-supported physical rehabilitation intervention to treat frailty in a pilot study of 18 lung transplant recipients.	Aidcube, a customizable rehabilitation mHealth platform.	pulmonary rehab	**Who provided**: physiotherapist<br><br>**Where:** therapist unknown location, patient in their own homes.	full telerehab asynchronous Aidcube, a customizable rehabilitation mobile health platform; email	Over the course of 20 months, a total of 18 subjects were enrolled in this pilot study<br><br>8 weeks<br><br>daily exercise prescription<br><br>The exercise prescription was advanced in a non-structured format by adding Time for aerobic activities, repetitions and/or sets for strength exercises, or adding new exercises based on subject level feedback on level of difficulty and evaluation by the physical therapist. There was no protocoled formula for advancing the patient's prescription; the decision and changes were made at the discretion of the physical therapist.	**Tailoring:** The exercise prescription was advanced based on subject level feedback on level of difficulty and evaluation by the physical therapist.<br>**Modifications:** N/A<br>**Adherence:** Among the 18 subjects who completed enrollment, 17 (94%) also completed closeout SPPB assessments; one patient was lost to follow-up as part of the study.	0 adverse events/18 participants<br>0 adverse events/1064 sessions<br>severity: N/A<br>type: N/A<br>relatedness: N/A<br>description: N/A
Dennett et al. [76]	**Publication Year**: 2021<br>**Post-Covid**<br>**Sample Size:** total participants = 123<br>**Mean age:** 65<br>**Sex:** 57% women<br>**Country of origin:** Australia<br>**Population:** cancer	mixed methods	Following the rapid implementation of an exercise-based telerehabilitation program in response to COVID-19, a process evaluation was conducted to understand the impact on patients, staff, and the health service with the aim of informing future program development.	Health coaching (videoconference or telephone)<br>Optional online group exercise (live videoconference via WebEx)<br>Optional online group multidisciplinary education (live videoconference via WebEx)<br>Written or app-based (Physitrack), individualized home exercise program on completion exercise program and exercise band<br>Online information portal (iLearn) with recordings of multidisciplinary education, information handouts, and weblinks or written information handouts<br>Participants were offered a referral to a community exercise program on completion	cancer rehab<br>Aerobic: walking, aerobics, step-ups<br>Resistance: exercise bands, body weight exercise, free weights<br>Flexibility: included as required based on individual needs	**Who provided:** nurse coordinator, 3 physiotherapists, and an allied health assistant<br><br>**Where:** therapist hospital based, patient home based	full telerehab synchronous video conferencing or telephone, app exercise program, online information portal iLearn with recordings of multidisciplinary education, info handouts, and weblinks or written info handouts	8 weeks<br>Aerobic: walking, aerobics, step-ups<br>Resistance: exercise bands, body weight exercise, free weights<br>Flexibility: included as required based on individual needs<br><br>Intensity • Aerobic: moderate (BORG 3-4)<br>• Resistance: 2-3 sets 10-12 repetitions<br>Frequency • 1x weekly health coaching •<br>• 1x weekly online group supervised training<br>• 1x weekly group education<br><br>Session time • 30-minute 1:1 health coaching reviews<br>• 45-minute online exercise group (live)<br>• 45-minute online education group (live)	**Tailoring:** Individualized exercise program based on initial consultation and goals<br>**Modifications:** N/A<br>**Adherence:** Adherence to health coaching was high (674/843, 80% of scheduled sessions),<br>but participation in (optional) online group exercise classes was low (n=36, 29%).	27 adverse events/123 particiapnts<br>27 adverse events/ 984 total sessions<br>severity: minor<br>type: physical<br>relatedness: N/A<br>description: musculoskeletal pain or strain

Cox et al. [56]	**Publication Year:** 2022<br>**Post-Covid**<br>**Sample Size:** intervention group = 68, control group = 67, total participants = 135<br>**Mean age:** 68<br>**Sex:** 60.2% women<br>**Country of origin:** Australia<br>**Population**: chronic respiratory disease	RCT	investigated whether home-based telerehabilitation was equivalent to centre-based pulmonary rehabilitation in people with chronic respiratory disease.	The telerehabilitation equipment 'kit' comprised: a step-through exercise bike to maximise safety, a 4G enabled tablet computer with mobile data, fixed to a stand for video conferencing; and a pulse oximeter to monitor peripheral oxygen saturation and pulse rate during training and at rest	pulmonary rehabilitation - aerobic and resistance training,	**Who provided:** physiotherapist<br><br>**Where:** therapist unknown location, patient in their own homes.	hybrid telerehab synchronous video conferencing	Their initial exercise training session was undertaken during a home-visit with the physiotherapist. After the initial home-visit, the remaining 15 telerehabilitation sessions were conducted in a virtual group of up to six participants, two times per week over 8 weeks.<br>30 min of cycle training, in two or more bouts, in each telerehabilitation session. Work rate equivalent to 60% of the peak oxygen uptake (VO2) on a cardiopulmonary exercise test (CPET). Intensity of cycle training will be progressed each week by 5–10% of the initial workload as tolerated, based on patient symptoms.<br>Resistance training for the arms and legs will utilise equipment readily available in the home environment. Initially prescribed as tolerated, to achieve 8–12 repetitions for 3 sets of each exercise. Encouraged to perform an additional 3 unsupervised sessions each week, documented in a home diary that is reviewed weekly by the supervising physiotherapist.	**Tailoring:** Individualized based on symptoms, CPET, etc.<br>**Modifications:** N/A<br>**Adherence:** The mean number of exercise training sessions attended by participants was 13 sessions/16 sessions	0 adverse events/ 68 participants<br>0 adverse events/1020 sessions<br>severity: N/A<br>type: N/A<br>relatedness: N/A<br>description: N/A
Correia et al. [100]	**Publication Year**: 2018<br>**Pre- Covid**<br>**Sample Size:** intervention group = 38, control group = 31, total participants = 69<br>**Mean age:** 67.3<br>**Sex**: 84.2% women<br>**Country of origin:** Portugal<br>**Population:** Total Knee Replacement	quasi	compare the clinical outcomes of a home-based program using this system against conventional in-person home-based rehabilitation after TKA, as well as assess patient uptake and safety of this novel feedback system.	novel digital biofeedback system for home-based physical rehabilitation (SWORD). Using inertial motion trackers, this system digitizes patient motion and provides real-time feedback on performance through a mobile app. It also includes a web-based platform that allows the clinical team to prescribe, monitor and adapt the rehabilitation process remotely.	TKA total knee arthroplasty rehab<br>Weeks 0-2: open kinetic chain exercises without added resistance: lying, sitting, standing, strengthening of hip flexors and extensors, ice pack application<br><br>weeks 3-6: exercises with steps, open kinetic chain exercises with added resistance, progressing to closed kinetic chain exercises, with strengthening of knee flexors/extensors and knee stabilization, progression to standing exercises without support, ice pack application<br><br>weeks 7-8: eccentric strengthening exercises, exercises involving steps, multi-directional exercises, ice pack application	**Who provided:** physiotherapist<br><br>**Where:** therapist unknown location, patient in their own homes.	full telerehab asynchronous web-based portal, telephone	8 weeks starting between day 7 and day 10 after surgery. Instructed to perform exercise sessions between five and seven days a week but were not excluded from the study in case of lower adherence.	**Tailoring:** N/A<br>**Modifications:** N/A<br>**Adherence:** 7 patients withdrew consent on the first week of the study, and one additional patient was excluded due to a protocol breach (additional physical therapy program started) corresponding to a 21% dropout rate in this group.	1 adverse event/38 participants<br>1 adverse event/1520 sessions<br>severity: N/A<br>type: physical<br>relatedness: N/A<br>description: thrombophlebitis
Coronado et al. [96]	**Publication Year:** 2021<br>**Post-Covid**<br>**Sample Size:** total participants = 8<br>**Mean age:** 53.4<br>**Sex:** 62.5% women<br>**Country of origin:** USA<br>**Population:** anterior cervical discectomy and fusion	mixed methods	describe the safety, feasibility, and preliminary outcomes of an early telephone-supported home exercise program (HEP) performed within the first 6 weeks after anterior cervical discectomy and fusion (ACDF) surgery.	telephone	HEP including daily walking, deep breathing, distraction techniques, and cervical and upper body exercises.	**Who provided:** physiotherapist<br><br>**Where**: therapist unknown location, patient in their own homes.	full telerehab asynchronous telephone	a 6-week HEP including daily walking, deep breathing, distraction techniques, and cervical and upper body exercises. Patients were instructed to perform each exercise daily for 6 weeks.	**Tailoring:** progressed as indicated<br>**Modifications:** N/A<br>**Adherence:** The HEP was performed an average of 32 days (76%) over the 6-week period.	unknown adverse events/8 patients<br>unknown adverse events/256 sessions<br>severity: N/A<br>type: physical<br>relatedness: N/A<br>description: shoulder and arm soreness, transient numbness in both arms

Cooley Hidecker et al. [83]	**Publication Year:** 2022<br>**Post Covid**<br>**Sample Size:** total participants = 15<br>**Mean age:** 73.3<br>**Sex:** 53.33% women<br>**Country of origin:** USA<br>**Population:** Parkinson's disease	quasi	test the feasibility, safety, and signal of efficacy of a coordinated telehealth program, consisting of speech therapy, physiotherapy, and pharmaceutical care, for people with PD living in some rural US communities.	Zoom, laptop computer equipped with necessary software	SLP (hierarchical drill and practice sessions), physio (aerobic, strength, balance, active rest and stretching), pharmaceutical care program	**Who provided:** four SLPs, two physiotherapists, two clinical pharmacists<br><br>**Where:** therapist unknown location, patient in their own homes.	full telerehab synchronous Zoom	Speech therapy: four 60-minute sessions per week for the first 4 weeks of the program.<br><br>Physiotherapists supervised the real time, one-on-one, 60-minute physiotherapy program once per week for the 8 weeks. Participants were also assigned a participant-specific home exercise program to complete two additional 60-minute sessions per week.<br><br>All medication management sessions were scheduled for up to 60 minutes (ranging from 15 minutes to 60 minutes, depending on the number of medications taken by the participant and the number of drug therapy problems identified) per week for the 8-week study	**Tailoring:** physiotherapists tailored the intensity and the modality of the exercise within the study parameters<br>**Modifications:** N/A<br>**Adherence:** All 15 participants of the 8-week intervention completed more than 80% of their scheduled treatment sessions	11 adverse events/30 patients<br>11 adverse events/360 sessions<br>severity: non-serious<br>type: physical<br>relatedness: N/A<br>description: hoarse voice, sore throat, strained neck, coughing, strain/sprain, muscle soreness
Colon-Semenza et al. [84]	**Publication Year:** 2023<br>**Post-Covid**<br>**Sample Size:** total participants = 55<br>**Mean age:** 69.5<br>**Sex**: 34.55% women<br>**Country of origin:** USA<br>**Population:** Parkinson's disease	mixed methods	To (1) determine the characteristics and participation rate of adults with Parkinson disease (PD) in physical therapy (PT) delivered via telehealth, (2) identify the outcome measures and interventions implemented, (3) determine the safety of and (4) patient and therapist satisfaction with PT via telehealth in a clinic specializing in the care of people with PD during the coronavirus pandemic.	virtual meeting platform, screened home for pre-existing types of devices (smartphone, tablet, desktop/laptop computer, web-<br>cams, etc.) available, physical space, exercise (e.g., treadmill) and medical equipment<br>(e.g., home blood pressure monitor) available,	therapeutic exercise and balance training, gait training	**Who provided:** physiotherapist<br><br>**Where:** therapist unknown location, patient in their own homes.	full telerehab synchronous virtual meeting platform	There was a total of 227 telehealth sessions over the reviewed<br>period, with a mean of 4.4 (SD 3.3) sessions per patient. The mean treatment duration was 5.3weeks (SD 4.2) and ranged from 1 to 15weeks. The most common frequency of treatment was 1x per week. The intervention most frequently implemented (Table 4) was therapeutic exercise (74%, 41/55). This was followed by gait and balance training (54.5%, 30/55) and patient education (43.6%, 24/55). Only four patients received treatment that was focused on functional activity training.	**Tailoring:** individualized plan of care.<br>**Modifications:** N/A<br>**Adherence**: 71.4% participation rate.	0 adverse events/55 participants<br>0 adverse events/227 sessions<br>severity: N/A<br>type: N/A<br>relatedness: N/A<br>description: N/A
Colas et al. [44]	**Publication Year**: 2022<br>**Post-Covid**<br>**Sample Size**: intervention group = 9, control group = 8, total participants = 17<br>**Mean age:** 52.2<br>**Sex:** 47% women<br>**Country of origin:** France<br>**Population:** COVID	quasi	assess fatigue in patients with prolonged symptoms after COVID-19<br>infection and who received a mixed program of remote adapted physical activity and therapeutic education. evaluate the efficacy and safety<br>of this training method thanks to aerobic and anaerobic parameters.	secure video conferencing software (Cisco Webex Meetings, equipment was composed of a connected device with frontal camera (smartphone, tablet, laptop computer, or computer and webcam) and a microphone (a headset can be used for a better sound quality); a good quality internet connection. For exercise sessions, the patient needed to have an exercise bike, a floor mat, and a sufficient and calm work space. connected watch to monitor heart rate.	endurance training, strength training	**Who provided:** physiotherapist<br><br>**Where:** therapist unknown location, patient in their own homes.	hybrid telerehab synchronous video conferencing	Supervised sessions were realized at home by videoconferencing for 3 weeks (three live sessions of 1 h/week, 45 min of aerobic exercise, and 15 min of resistance exercise; Aerobic training was performed at ventilatory threshold 1 (continuous training) the 1st week and progressed to intermittent work at ventilatory threshold 2 the last week (Figure 2). Resistance training was performed in whole body circuit training with body weight from a light intensity to a moderate intensity according to perceived exertion using a modified Borg scale from 0 (no exertion)<br>to 10 (extremely hard exertion).	**Tailoring:** exercise program was personalized based on the results of the functional tests carried out during the initial evaluation.<br>**Modifications:** N/A<br>**Adherence:** Participants achieved an average of 81% of the sessions	0 adverse events/9 participants<br>0 adverse events/27 sessions<br>severity: N/A<br>type: N/A<br>relatedness: N/A<br>description: N/A

Coats et al. [57]	**Publication Year:** 2020<br>**Pre-Covid**<br>**Sample Size:** total participants = 5<br>**Mean age:** 62<br>**Sex:** 40% women<br>**Country of origin:** Canada<br>**Population:** lung cancer	quasi	investigate the feasibility, adherence and satisfaction of a home-based telerehabilitation program (TELERP) with real-time physiological parameters acquisition in patients with unresectable thoracic neoplasia receiving chemotherapy and to explore its effects on patients' functional capacity.	eChez-Soi telerehabilitation platform. "All-in-one" computer with a touch screen and Windows 8 friendly user interface, as well as a small external screen, a webcam, all sensors and instruments for bio-mechanical and physiological measurement and the software solution. videoconferencing solution Vidyo (VidyoTM Desktop Software) weight scale (AriaTM Wi-Fi Smart Scale) and blood pressure (A&D Medical Blood Pressure Monitor) Exercise ball and elastics, Xbox Dance Mat and a Wii Balance Board (NintendoVR Wii FitTM), via a game software environment developed for this purpose. educational intervention through short presentations given remotely by videoconference via the telerehabilitation platform.	warm-up, Cardiorespiratory exercises, resistance exercises	**Who provided:** clinical exercise physiologist/cancer exercise trainer certified by the American College of Sports Medicine.<br><br>**Where:** therapist unknown location, patient in their own homes.	full telerehab synchronous video conferencing	8 weeks. 15 supervised sessions and 9 unsupervised sessions were planned for a total of 24 exercise sessions. The mean duration of supervised sessions was 67 ± 12 minutes.<br>20 minutes of cardiovascular exercises per session at moderate intensity (corresponding to a heart rate between 60–80% of the VO_ 2peak measured by the cardiorespiratory test) with brief period of high intensity (>80% of the VO_ 2peak).<br><br>Exercise ball and elastics were used to train muscle groups of the upper limbs (biceps curl, wall pushup, lateral shoulder raise) and lower limbs (wall squat and lunges). Ten repetitions of each movement were initially performed. The number of repetitions was progressively increased, as tolerated, until two sets of 15 repetitions were achieved.	**Tailoring:** individualized exercise prescription,<br>**Modifications:** N/A<br>**Adherence:** All patients completed 100% of the supervised prescribed exercise sessions (15/15 sessions prescribed) over 8 weeks	0 adverse events/5 participants<br>0 adverse events/75 sessions<br>severity: N/A<br>type: N/A<br>relatedness: N/A<br>description: N/A
Choi et al. [55]	**Publication Year:** 2016<br>**Pre-Covid**<br>**Sample Size:** total participants = 4<br>**Mean age:** 55<br>**Sex:** 25% women<br>**Country of origin:** USA<br>**Population**: lung transplant	mixed methods	We evaluated the feasibility, safety, system usability, and intervention acceptability of Lung Transplant Go (LTGO), an 8week in-home exercise intervention for lung transplant recipients using a telerehabilitation platform and described changes in physical function and physical activity from baseline to post-intervention.	telerehabilitation platform, Versatile and Integrated System for TeleRehabilitation (VISYTER) including (1) video conferencing, (2) camera control that allowed the interventionist to remotely change the visual field using the pan-tilt-zoom mode, (3) remote real-time demonstration of exercise and observation of responses, and (4) eye contact and a teleprompter, an essential feature to help participants perceive credibility and promote flow of communication. Exercise diary, pulse oximeter, pedometer, and automatic blood pressure monitor used.	warm-up, cool-down, strengthening exercises, aerobic exercise, balance exercises	**Who provided:** physiotherapist, exercise physiologist<br><br>**Where:** therapist unknown location, patient in their own homes.	full telerehab synchronous video conferencing	weekly basis, 8 weeks, Weekly LTGO sessions (≈ 40 minutes each x 8 sessions): Exercises included warm-up and cool down exercises, strengthening exercises (using cuff weights), aerobic exercise (walking) and balance exercises to promote endurance, flexibility, balance, and strengthening. Exercise prescription and progression were based on guidelines of the American College of Sports Medicine (ACSM) and American Association of Cardiovascular and Pulmonary Rehabilitation Participants are instructed to practice their exercises daily and keep an exercise diary, monitor SpO2 and blood pressure, and record daily steps (with a pedometer).	**Tailoring:** The interventionist (1) reviews the participant's baseline physical function and clinical data, (2) discusses participant's goals and preference and (3) develops a daily exercise regimen.<br>**Modifications:** N/A<br>**Adherence**: Three participants completed eight sessions and one (# 2) completed 7 of 8 sessions.	0 adverse events/4 participants<br>0 adverse events/31 sessions<br>severity: N/A<br>type: N/A<br>relatedness: N/A<br>description: N/A

Chen et al. [74]	**Publication Year:** 2021<br>**Post-Covid**<br>**Sample Size**:<br>intervention group = 40, control group = 40, total participants = 80<br>**Mean age:** 59.6<br>**Sex:** 27.5% women<br>**Country of origin:** China<br>**Population**: esophageal cancer	RCT	The outbreak of COVID-19 imposed physical and emotional obstacles for traditional face-to-face rehabilitation. Meanwhile, the effectiveness of telerehabilitation remained unknown. In this study, we aimed to investigate the effectiveness of telerehabilitation.	small tips, training videos, and regular online consulting, WeChat group	All patients received standard consultation whenever they returned to the thoracic surgery clinic. The telerehabilitation group received additional online consulting and training, including (I) precautions for nutritional support; (II) swallowing function training; (III) respiratory function training; (IV) guidance and feedback on matters such as patient's current vital signs, wound status, medication, and sleep status.	**Who provided:** medical care group which included the head of the nursing department in thoracic surgery, at least one doctor who participated in the surgery, and three experienced nurses.<br><br>**Where:** therapist unknown location, patient in their own home	hybrid telerehab synchronous intervention delivered by daily small tips, training videos, and regular online consulting on WeChat mobile app	3-month home-based telerehabilitation intervention after discharge, and a WeChat group including the medical care group, the patient, and the patient's family was created at discharge.<br>frequency, intensity not reported.	**Tailoring**: N/A<br>**Modifications**: N/A<br>**Adherence**: N/A	0 adverse events/40 participants<br>severity: N/A<br>type: N/A<br>relatedness: N/A<br>description: N/A
Cerdan de Las Heras et al. [103]	**Publication Year**: 2022<br>**Post-Covid**<br>**Sample size**:<br>intervention group = 15, control group = 15, total = 30<br>**Mean age**: 51.6<br>**Sex:** 33.33% women<br>**Country of origin:** Denmark<br>**Population**: sarcoidosis	RCT	investigated the usefulness and effectiveness of TR on exercise capacity in patients with sarcoidosis. Method:	Virtual Autonomous Physiotherapist Agent (VAPA)(17) - a service platform for therapists to create customized rehabilitation programs, video consultations, e-learning packages, physical exercise programs, online questionnaires, patient digital file and direct chat function in the same tool. mobile app that patients can install on a smartphone or tablet is connected directly with a biometric sensor attachable to the patient's chest, arms or fingers to collect data and adjust the rehabilitation program in real time. kit composed of a smart tablet and a biometric sensor to track pulse	aerobic and strength training	**Who provided:** physiotherapist<br><br>**Where:** therapist unknown location, patient in their own homes.	hybrid telerehab asynchronous video consultations, direct chat on mobile app	12 week asynchronously supervised study period - The physiotherapist programmed VAPA to motivate the participant to physically train at least 60 min a week without pauses between exercises, creating tailored exercise sets divided in 4 different intensity categories combining exercises from 250 aerobic and strength 3D exercises stored in a digital database. Up to 6 online meetings were scheduled with the participant to follow-up on improvements and to adjust the training program during the first 12 weeks. After this period patients were offered daily use of VAPA during follow-up but without physiotherapist guidance.	**Tailoring:** The physiotherapist individualized the telerehabilitation program based on baseline 6-minute walk test and a first interview focused on participant daily activity.<br>**Modifications:** N/A<br>**Adherence:** Exercise adherence in the intervention group was 64% and average exercise time was 28 minutes per exercise session during the first 3 months.	0 adverse events/15 participants<br>0 adverse events/180 sessions<br>severity: N/A<br>type: N/A<br>relatedness: N/A<br>description: N/A

Capin et al. [45]	**Publication Year**: 2022<br>**Post-Covid**<br>**Sample size**: intervention group = 28, control group = 13, total participants = 41<br>**Mean age:** 52<br>**Sex:** 46% women<br>**Country of origin:** USA<br>**Population**: COVID	RCT	Determine the safety, feasibility and initial efficacy of a multicomponent telerehabilitation programme for COVID-19 survivors.	Zoom, a package of materials including an automated blood pressure cuff, pulse oximeter, Kindle Fire tablet (Amazon Inc, Seattle, Washington, USA), Fitbit Inspire 2 activity monitor (Fitbit, San Francisco, California, USA) and an equipment instruction manual. ankle weights and resistance bands, Health in Motion application used for self-directed intervention outside of supervised sessions, and enabled the physical therapist to monitor remotely the patient's adherence to the app-guided exercises and education.	breathing and clearance techniques, high intensity strength training (8-rep max), aerobic/cardiovascular exercise, balance exercises, functional activities, stretching, lifestyle coaching/motivational interviewing	**Who provided:** physiotherapist<br><br>**Where**: therapist unknown location, patient in their own homes.	full telerehab hybrid (synchronous and asynchronous) video conferencing	12 individual, supervised telerehabilitation sessions were provided three times per week in week 1, twice per week in weeks 2–4, once per week in weeks 5–6 and a single 'booster' visit session during week 9 or 10.<br>- breathing and clearance techniques (based on symptoms, needs, and goals, often 1-5x per day for 5-15 mins)<br>- high intensity strength training (8-rep max)<br>- aerobic/cardiovascular exercise (low intensity: focus on increasing duration, high intensity: focus on increasing intensity (pace, resistance) and/or number of short intervals (ranging from 10s to 3-5 min)<br>- balance exercises (target difficulty level that achieves 50-80% success rate<br>- functional activities (based on symptoms, needs, goals)<br>- stretching (typically 2-3 sets of 30s per stretch)<br>- lifestyle coaching/motivational interviewing (based on symptoms, needs, goals)	**Tailoring:** individual biobehaviourally informed, app-facilitated, multicomponent telerehabilitation sessions<br>**Modifications**: N/A<br>**Adherence:** 27 of 29 participants (93%; 95% CI 77% to 99%) receiving the intervention attended ≥75% of sessions.	0 adverse events/28 participants<br>0 adverse events/336 sessions<br>severity: N/A<br>type: N/A<br>relatedness: N/A<br>description: N/A
Campbell et al. [94]	**Publication Year**: 2022<br>**Post-Covid**<br>**intervention** = 17, control = 56, total participants = 73<br>**Mean age**: 38.34<br>**Sex:** 52.94% women<br>**Country of origin:** USA<br>**Population:** mild traumatic brain injury	RCT	explored feasibility and adoption of an in-person rehabilitation program for subacute mTBI delivered through telerehabilitation.	video conferencing (Webex). Daily home exercise program and a log, treadmill	exercises under 4 subcategories: (1) cervical spine, (2) cardiovascular, (3) static balance, and (4) dynamic balance	**Who provided:** physiotherapist<br><br>**Where:** therapist unknown location, patient in their own homes.	full telerehab synchronous video conferencing	8 visits virtually, completed over 6 weeks. The frequency of visits began with 2 visits in the first 2 weeks, and then reduced to once per week for the remaining 4 weeks. Each visit was 60 minutes.<br>exercises under 4 subcategories:<br>(1) cervical spine, (2) cardiovascular (walking or jogging on a treadmill at 80% of their symptom-provoking HR for each session, determined by initial visit BCTT results, progressed by increasing their HR 5 beats/minute (bpm) every 5 minutes by increasing either treadmill speed or incline with minimal increase in symptoms (≤2 points), (3) static balance, and (4) dynamic balance	**Tailoring:** intensity of cardiovascular exercise determined by initial visit BCTT results<br>**Modifications**: N/A<br>**Adherence:** telerehabilitation withdrawal rate 12%, high session attendance 97%,	0 adverse events/17 participants<br>0 adverse events/136 sessions<br>severity: N/A<br>type: N/A<br>relatedness: N/A<br>description: N/A
Brocki et al. [38]	**Publication Year**: 2022<br>**Post-Covid**<br>**Sample Size**: total participants = 5<br>**Mean age:** 83 (median), 74-87 range<br>**Sex:** 40% women<br>**Country of origin**: Denmark<br>**Population:** transcatheter aortic valve implantation	mixed methods	to examine the feasibility of exercise-based cardiac telerehabilitation after TAVI.	tablet (iPad, Apple) along with a SIM card for data coverage. For the web-based training sessions, we used an encrypted videoconferencing system (Videosamtale)	cardiac rehab, aerobic and strength training	**Who provided:** physiotherapist<br><br>**Where**: therapist in hospital, patient in their own homes.	hybrid telerehab synchronous video conferencing	The number of web-based sessions was set at 5. combination of aerobic and strength training twice weekly, with each session lasting 30 to 45 minutes. target intensity for the aerobic exercises was either a heart rate of 80 to 100 beats/min. Patients were offered further sessions if they were able to attend. In addition, the patients were instructed to take a 30-minute walk daily with moderate intensity. Before hospital discharge, the patients were instructed to perform 3 exercises on alternate days until the home visit took place (home exercise program)	**Tailoring:** individualization of exercises<br>**Modifications:** N/A<br>**Adherence**: 60% (8/13) included patients did not complete the study. Number of training sessions per participant ranged from 2 (n=1) to 7 (n=1),	0 adverse events/5 participants<br>0 adverse events/25 sessions<br>severity: N/A<br>type: N/A<br>relatedness: N/A<br>description: N/A

Bianchini et al. [85]	**Publication Year:** 2022<br>**Pre-Covid**<br>**Sample Size:** total participants = 23<br>**Mean age**: 64.1<br>**Sex**: 43.5% women<br>**Country of origin:** Italy<br>**Population**: Parkinson's disease	quasi	investigate feasibility, safety, and efficacy of telerehabilitation in mild-to-moderate PD patients.	digital platform for telemedicine freely available by Regione Lazio, named "Salute Digitale" with easy-to-access audio/video remote conference call interface, tablet, laptop, or computer/webcam), diary for self-conducted sessions.	general mobility, static, and dynamic balance, coordination, dexterity, postural transitions, and facial mobility.	**Who provided:** physiotherapist<br><br>**Where:** therapist unknown location, patient in their own homes.	full telerehab synchronous video conferencing	5-week telerehabilitation program consisting of a remote session with a physiotherapist once weekly and at least two self-conducted sessions per week. patients had free access to video tutorials, showing the exercises performed with physiotherapists and were instructed to exercise at least twice weekly with a minimum of 30 min for each session. A number of exercises ranging from 8 to 12, for duration of 40–60 min were included in each session depending on the patients' condition, functional demands, and reported difficulties.	**Tailoring:** adjusted depending on the patients' condition, functional demands, and reported difficulties.<br>**Modifications**: N/A<br>**Adherence:** The dropout rate was 0%. Over 85% of patients reached acceptable adherence cut-off and around 70% reached optimal one.	0 adverse events/23 participants<br>0 adverse events/115 sessions<br>severity: N/A<br>type: N/A<br>relatedness: N/A<br>description: N/A
Benvenuti et al. [69]	**Publication Year**: 2014<br>**Pre-Covid**<br>**Sample size**: intervention group = 143, control group = 45, total sample size = 188<br>**Mean age:** 69.1<br>**Sex:** 73.7% women<br>**Country of origin**: Italy<br>**Population:** Upper limb paresis	quasi	evaluate the safety, acceptance, adherence, and effectiveness of a community-based exercise program for upper limb paresis in patients with chronic stroke and the effects of telerehabilitation monitoring in kiosks distributed through the community.	Habilis platform (Web-based platform). Kiosks with computers with touch screens and webcams located in social centers or voluntary associations in local municipalities. The exercises used very simple rehabilitation devices, low cost and easily available in the community (different shape and size objects to manipulate, puzzles, printed paths to follow with pens of different shape and size, etc).<br><br>material for home practice was given to each subject in a "training suitcase" by the hospital physiotherapist. Home Program Sheet that specified their individualized exercises prescription. Logbook.	exercises that encompassed the essential compo-nents of reaching, grasping, holding and manipulation	**Who provided:** physiotherapist<br><br>**Where:** therapist unknown location, patient at kiosk in social centers or voluntary associations in local municipalities	full telerehab synchronous video conferencing	instructed to practice the exercises at least twice a week at the kiosk and at least 3 more days a week at home.<br>subjects were progressed through exercises that encompassed the essential components of reaching, grasping, holding and manipulation with increasing level of difficulty. The level of difficulty was chosen so as to make the task "challenging, but not impossible."	**Tailoring:** individualized the exercise prescription, based on characteristics of the impairments observed in baseline and follow-up assessments.<br>**Modifications:** N/A<br>**Adherence:** Only 30% of the subjects attended kiosks regularly. Only 50 of the 165 (143 treatment group + 22 from control group accepting treatment after the effectiveness study completed) were highly adherent to the study protocol, 85 demonstrated average adherence, and 30 low adherence.	0 adverse events/143 participants<br>0 adverse events/3718 sessions<br>severity: N/A<br>type: N/A<br>relatedness: N/A<br>description: N/A
Batalik et al. [37]	**Publication Year**: 2021<br>**Post-Covid**<br>**Sample Size**: total participants = 19<br>**Mean age:** 60.4<br>**Sex:** 26.31% women<br>**Country of origin:** Czech Republic<br>**Population:** coronary heart disease	quasi	investigated an alternative home-based cardiac telerehabilitation model in consideration of the recommendations for the COVID-19 quarantine of people diagnosed with coronary heart disease (CHD).	PolarFlow web platform. Polar M430 heart rate (HR) monitor and an H10 chest sensor (Kempele, Finland) compatible with the web platform.	cardiac rehab, exercises aimed at improving cardiorespiratory fitness	**Who provided:** physiotherapist<br><br>**Where:** therapist unknown location, patient in their own homes.	full telerehab asynchronous telephone	8 week intervention, perform regular physical exercise 3–5 times per week, for a minimum of 30 min at the target HR zone determined by calculation based on the 200 m fast walking test (200 mFWT) result.	**Tailoring:** at the target heart rate zone determined by calculations based on individual 200 mFWT results.<br>**Modifications:** N/A<br>**Adherence:** A total of 84% of participants completed the 8-week intervention.	0 adverse events/19 participants<br>0 adverse events/456 - 760 sessions<br>severity: N/A<br>type: N/A<br>relatedness: N/A<br>description: N/A
Batalik et al. [25]	**Publication Year:** 2020<br>**Pre-Covid**<br>**Sample Size**: intervention = 25, control = 26, total participants = 51<br>**Mean age:** 56.5<br>**Sex:** 20% women<br>**Country of origin:** Czech Republic<br>**Population:** cardiac patients	RCT	the use of the wrist heart rate monitor as a TR device, defines detected limitations, and compares the effect between home-based TR and regular outpatient CR methods related to physical fitness, QOL, and training adherence.	Polar Flow web application, wrist heart rate monitor M430 (Polar, Kempele, Finland) monitoring heart rate, time, training mode, duration, and distance of training physical activity, mobile phone and free internet access), educational booklet (healthy diet advice, cardiovascular risk factors management, and smoking cessation,	cardiac rehab	**Who provided:** physiotherapist<br><br>**Where:** therapist unknown location, patient in their own homes.	full telerehab asynchronous telephone	3 times a week for 12 weeks altogether. One session consisted of 10 minutes warm-up, 60 minutes aerobic phase (walking or cycling, according to predefined training heart rate set at 70–80% heart rate reserve), and 10 minutes cool-down phase.	**Tailoring:** N/A<br>**Modifications:** N/A<br>**Adherence**: ITG performed 31.7±8.9 training units (88.2% of all sessions, ranging from 10 to 48).	0 adverse events/25 participants<br>0 adverse events/900 sessions<br>severity: N/A<br>type: N/A<br>relatedness: N/A<br>description: N/A

Ashikaga et al. [39]	**Publication Year**: 2023<br>**Post-Covid**<br>**Sample Size:** intervention = 11, control = 6, total participants = 17<br>**Mean age:** 80.4<br>**Sex:** 45% women<br>**Country of origin:** Japan<br>**Population:** TAVI	quasi	investigate the efficacy of HBTR in patients who have undergone TAVI.	telemonitoring app from the tablet PC (iPad), a blood pressure manometer. and a pulse oximeter, wireless ECG transmitter, cycle ergometer	cardiac rehab	**Who provided:** physiotherapist<br><br>**Where:** therapist unknown location, patient in their own homes.	hybrid telerehab synchronous video conferencing	12-week stage, performed HBTR twice weekly. The target intensity was based on the AT from the CPET at the start of the second stage. exercise duration was initially at 15 minutes, was gradually increased to 30 minutes within the first 2 weeks. Exercise load was arranged according to the participant's perceived exertion (ie, a score of 11-13 on the Borg scale). Participants were instructed to perform 3 sets of 10 repetitions of resistance training (standing calf raises and sit-to-stand exercises) every day. The medical staff checked whether the participants were able to perform the resistance training every day during every video call.	**Tailoring:** The target intensity was based on the AT from the CPET at the start of the second stage.<br>**Modifications**: N/A<br>**Adherence:** All patients underwent 24 HBTR sessions during the 12-week training period. (24/24)	0 adverse events/11 participants<br>0 adverse events/264 sessions<br>severity: N/A<br>type: N/A<br>relatedness: N/A<br>description: N/A
Alwakeel et al. [51]	**Publication Year:** 2022<br>**Pre-Covid**<br>**Sample Size:** intervention = 177, control = 89, total participants =266<br>**Mean age**: 68.4<br>**Sex:** 55.9% women<br>**Country of origin**: Canada<br>**Population**: COPD	quasi	provide information on implementing a novel province-wide standardized community-based tele-PR program and determine its accessibility, feasibility, and safety.	video conferencing, exercise equipment, (free weights and stationary bicycle and/or treadmill)), exercise programs and education based on the Living Well with COPD (LWWCOPD),	pulmonary rehabilitation, cardiovascular and strengthening component	**Who provided:** a PR physician, nurse, physiotherapist, occupational therapist, respiratory therapist, and nutritionist.<br><br>**Where:** therapists at Mount Sinai, patients at tele-PR sites (Tele-PR sites were community-based primary and secondary healthcare centers, have access to appropriate space and exercise equipment (free weights and stationary bicycle and/or treadmill))	Hybrid telerehab synchronous video conferencing	The program was offered five times per year with 2-week breaks between each program. Each program was 8 weeks with three sessions per week. Sessions lasted 2 hours and were divided into 1 hour of education and 1 hour of exercise. The exercise program included a 30-minute cardiovascular and 30-minute strengthening component	**Tailoring:** N/A<br>**Modifications**: N/A<br>**Adherence:** the tele-PR program had an 83% (177 out of 214) completion rate.	0 adverse events/266 participants<br>0 adverse events/4248 sessions<br>severity: N/A<br>type: N/A<br>relatedness: N/A<br>description: N/A
Ackerley et al. [95]	**Publication Year:** 2023<br>**Post-Covid**<br>**Sample Size**: total participants = 109<br>**Mean age:** 57<br>**Sex**: 39% women<br>**Country of origin:** England<br>**Population:** neurological rehabilitation	mixed methods	to implement and evaluate a multidisciplinary group-based telerehabilitation approach for people engaging in neurological rehabilitation.	NeuroRehabilitation OnLine (NROL), online platform Microsoft Teams.	neurological rehabilitation talking (Cognitive education, Cognitive processing, Living Well, Fatigue, Dysarthria, Dysphasia and Bookclub) and physical (Balance & Mobility and Upper Limb) targeted therapy groups and community groups (NROL entry, exit, follow-up and Café NROL).	**Who provided:** multidisciplinary team comprising therapy staff (allied health professional and psychology), assistant practitioners and patient volunteers.<br><br>**Where:** therapist unknown location, patient in their own homes.	full telerehab synchronous video conferencing	over six-week recurring blocks. Most patients participated in 1 NROL block (55%), but some participated in 2 (36%), 3 (7%) or 4 (2%)**.**	**Tailoring:** Session content was iteratively modified to meet the requirements of NROL patient cohorts<br>**Modifications:** N/A<br>**Adherence**: Overall NROL attendance rate was 68%.	1 adverse event/109 participants<br>1 adverse event/265 sessions<br>severity: non-injury<br>type: physical<br>relatedness: N/A<br>description: fall

Wilson et al. [70]	**Publication Year**: 2021<br>**Pre-Covid**<br>**Sample Size**: intervention = 10, control = 7, total participants = 17<br>**Mean age**: 69.9<br>**Sex**: 30% women<br>**Country of origin:** Australia<br>**Population**: stroke patients with upper-extremity dysfunction following a unilateral stroke,	RCT	compare use of the EDNA system with an active control (Graded Repetitive Arm Supplementary Program—GRASP training) group using a parallel RCT design.	VR, EDNA software, 22-inch touchscreen tablet	four goal-based and three exploratory movement activities that require manipulation of handheld objects (or tangible user interfaces—TUIs) on the surface of the display. Unimanual movements involving the more-affected and less-affected hand are required, together with bimanual movement for the exploratory tasks.	**Who provided: o**ccupational therapist<br><br>**Where:** therapist unknown location, patient in their own homes.	full telerehab asynchronous phone as communication (VR study)	30-min of upper-limb training per session, with a minimum of three and maximum of four sessions per week, for an 8-week period	**Tailoring:** N/A<br>**Modifications:** N/A<br>**Adherence**: total number of training sessions completed by patients in the EDNA =28	0 adverse events/10 participants<br>0 adverse events/ 280 sessions<br>severity: N/A<br>type: N/A<br>relatedness: N/A<br>description: N/A
van der Kolk et al. [86]	**Publication Year**: 2019<br>**Pre-Covid**<br>**Sample Size**: intervention = 65, control = 65, total participants = 130<br>**Mean age**: 59.3<br>**Sex:** 35% women<br>**Country of origin:** Netherlands<br>**Population:** Parkinson's disease	RCT	evaluate the effectiveness of aerobic exercise—gamified and delivered at home, to promote adherence—on relieving motor symptoms in patients with Parkinson's disease with mild disease severity who were on common treatment regimes	customised tablet based motivational app, telephone for coaching, stationary home-trainer cycle/bike	aerobic exercise done on a stationary home-trainer (aerobic	**Who provided:** coach (physical therapists or research assistant).<br><br>**Where:** therapist unknown location, patient in their own homes.	hybrid telerehab asynchronous telephone	6 month intervention - cycle on a stationary home-trainer for 30–45 min (30 min aerobic and 15 min warming up and cooling down) at least three times per week, within a predetermined heart rate zone on the basis of their heart rate reserve. Lower boundary of the target heart rate zone was set between 50% and 70% of HRR and was gradually increased as patients became fitter during the trial; the upper boundary was set at 80% of HRR.	**Tailoring**: programmes were personalised to the patient's abilities to ensure all eligible patients could complete the programme.<br>**Modifications**: N/A<br>**Adherence**: 25% of participants completed the intervention ≥3 times per week, 54% of participants completed the intervention 2–3 times per week, 22% of participants completed the intervention<2 times per week	27 adverse events/65 participants<br>27 adverse events/4394 sessions<br>severity: non-serious, serious<br>type: N/A<br>relatedness: potentially related, unrelated<br>description: arthralgia/back pain, palpitations, vestibular disorder, vasovagal collapse, knee injury
Van De Winckel et al. [65]	**Publication Year**: 2018<br>**Pre-Covid**<br>**Sample Size**: total participants = 6<br>**Mean age**: 61<br>**Sex**: 50% women<br>**Country of origin:** USA<br>**Population:** stroke	quasi	assess the feasibility and safety of a telerehabilitation program consisting of tDCS and finger tracking training through questionnaires on ease of use, adverse symptoms, and quantitative assessments of motor function and cognition.	StarStim Home Research Kit - a Neoprene head cap with marked positions for electrode placement, a wireless cap mounted stimulator and a laptop control computer. Saline-soaked, 5 cm diameter sponge electrodes. remote access application (TeamViewer), video conferencing application (Skype), computer. repetitive finger tracking training system - included an angle sensor mounted to a lightweight brace and aligned with the metacarpophalangeal (MCP) joint of the index finger, a sensor signal conditioning circuit, and a target tracking application loaded on a table computer.	Home-based transcranial direct current stimulation plus tracking training therapy	**Who provided:** physical therapist<br><br>**Where:** investigator was at the university and the participant was at home.	Hybrid telerehab synchronous video conferencing	5 treatment sessions at home/assisted living	**Tailoring**: N/A<br>**Modifications**: N/A<br>**Adherence**: 100% adherence to the sessions and all recommended telerehabilitation	0 adverse events/6 participants<br>0 adverse events/30 sessions<br>severity: N/A<br>type: N/A<br>relatedness: N/A<br>description: N/A

Song et al. [36]	**Publication Year:** 2020<br>**Pre-Covid**<br>**Sample Size:** intervention = 48, control = 48, total participants = 96<br>**Mean age:** 54.17<br>**Sex**: 10.4% women<br>**Country of origin:** China<br>**Population**: Coronary Heart Disease	RCT	investigate the effects of telemonitored exercise rehabilitation on patients with coronary heart disease (CHD) in China.	telemonitoring software (MEMRS-CRS), smartphones, heart rate belts, Medicus monitoring device computer terminal,	walking	**Who provided**: "researchers" - cardiologist, research assistants.<br><br>**Where:** therapist unknown location, patient in their own homes.	full telerehab synchronous telephone/text messaging	given exercise prescription according to their results of CPET. Exercise prescription was set according<br>to their anaerobic threshold (AT). Exercise intensity<br>was determined based on heart rates (HR). The target<br>HR during exercise was set as heart rate@AT (HR@AT)<br>± 5 bpm. The exercise type was walking. Exercise frequency<br>was 3–5 times per week, with each exercise duration<br>of 30 min and 5–10 min of warm-up and relaxation<br>before and after exercise [<br>6 months?	**Tailoring:** given exercise prescription according to their results of CPET.<br>**Modifications**: N/A<br>**Adherence**: The exercise frequency of patients in group A was 5.1 ± 0.6 times a week	0 adverse events/48 participants<br>0 adverse events/6364 sessions<br>severity: N/A<br>type: N/A<br>relatedness: N/A<br>description: N/A
Snoek et al. [26]	**Publication Year:** 2020<br>**Pre-Covid**<br>**Sample Size:** intervention = 89, control = 90, total participants = 179<br>**Mean age**: 72.4<br>**Sex**: 22% women<br>**Country of origin**: Europe: Netherlands, Denmark, Spain, Switzerland, France<br>**Population**: cardiac patients	RCT	assess whether a 6-month guided mobile cardiac rehabilitation (MCR) program<br>is an effective therapy for elderly patients who decline participation in cardiac rehabilitation	heart rate monitor and smartphone with a special application, MobiHealth Rehabilitation™ portal	cardiac rehab	**Who provided:** researcher/nurse/physician<br><br>**Where:** therapist unknown location, patient in their own homes.	full telerehab asynchronous telephone	6 months of mobile telemonitoring guidance and in addition another 6 months without mobile telemonitoring<br><br>Patients are instructed to exercise while wearing the heart rate monitor at 5 days per week for at least half an hour at an individual selected level of intensity and self-chosen type of activity.	**Tailoring**: The outcomes of the maximal exercise tests on the first visit will enable to personalise the heart rate zones per patient in order to see (after every training/activity) in what zone the patient is training<br>**Modifications:** N/A<br>**Adherence:** N/A	12 adverse events/89 participants<br>12 adverse events/10680 sessions<br>severity: severe<br>type: physical<br>relatedness: unrelated<br>description: all-cause mortality, cardiovascular mortality, near sudden cardiac death, acute coronary syndrome, hospitalization cardiac reason, chronic coronary syndrome, endocarditis
Simpson et al. [66]	**Publication Year**: 2020<br>**Pre-Covid**<br>**Sample Size:** total participants = 10<br>**Mean age:** 73.6<br>**Sex**: 60% women<br>**Country of origin**: Australia<br>Population: stroke	quasi	determine whether using the internet, a tablet<br>application, and a chair sensor that connected to a therapist was feasible in monitoring adherence<br>and progressing a functional exercise at home.	therapist app and a participant system, connected via the Internet. Tablet, custom app connected via Bluetooth to a chair-based sensor, consisting of force-sensitive resistors mounted in a flexible belt wrapped around the seat to detect sitting, and wired to a Bluetooth controller module	stroke rehab, sit-to-stand exercises	**Who provided:** physiotherapist/physician<br><br>**Where:** therapist unknown location, patient in their own homes.	full telerehab asynchronous mobile application	4 weeks, 10 participants<br><br>184 sessions prescribed = 4.6 sessions/week/participant (frequency intensity time type were individualized based on baseline measures and performance).<br><br>A mean of 724 reps were prescribed = 18.1 reps/week/participant	**Tailoring**: Baseline measures of physical function informed the initial exercise prescription. The exercise challenge was progressed according to participant performance during the previous week<br>**Modifications**: N/A<br>**Adherence**: participants performed an average of 125% (range 85% to 175%) of prescribed sessions.	0 adverse events/10 participants<br>0 adverse events/224 sessions<br>severity: N/A<br>type: N/A<br>relatedness: N/A<br>description: N/A
Simpson et al. [46]	**Publication Year**: 2023<br>**Post-Covid**<br>**Sample Size**: total participants = 27<br>**Mean age:** 58<br>**Sex:** 43% women<br>**Country of origin:** UK<br>**Population:** COVID-19	RCT	investigate the feasibility and efficacy of pulmonary telerehabilitation for COVID-19 survivors.	Cisco WebEx video conference platform	pulmonary rehab - structured warm-up, guidance/demonstration and observations of exercises, consisting of cardiovascular, flexibility, strength-based movements, balance work and a cool down. Each session finished with a guided relaxation element.<br><br>Education session on relevant topics, followed by time for participants to socialise with their peers.	**Who provided:** physiotherapist<br><br>**Where:** therapist unknown location, patient in their own homes.	full telerehab synchronous video conferencing	12 exercise classes, six education events and opportunity for peer support.<br><br>Twice a week, for 6 weeks, participants completed a synchronised exercise session in a group of between three and five people, lasting 45–60 min<br><br>Participants received an individualised exercise programme and were advised to undertake exercise on up to three additional days each week.	**Tailoring**: N/A<br>**Modifications**: N/A<br>**Adherence**: Median (IQR) participation in the available exercise classes was 92% (83–100%). 11 participants had 100% attendance, and all but one participant attended ⩾50% of available classes.	2 adverse events/27 participants<br>2 adverse events/324 sessions<br>severity: serious<br>type: N/A<br>relatedness: unrelated<br>description: N/A

Sheehy et al. [93]	**Publication Year:** 2022<br>**Post-Covid**<br>**Sample Size:** total participants = 11<br>**Mean age:** 78<br>**Sex**: 36.36% women<br>**Country of origin**: Canada<br>**Population**: Mild Cognitive Impairment	mixed methods	to determine if it is feasible and safe to use home-based VR exercise, focusing on physical and cognitive function, in individuals with MCI; and to obtain pilot data on a battery of physical and cognitive clinical outcome measures, administered before and after a 6-week home-based VR exercise program.	Jintronix nonimmersive VR software, Alienware Alpha desktop gaming computer, color camera and infrared sensors on a Kinect camera, logbook	Several games were available to train sitting and standing balance, reaching, and stepping. One game (with several variations) was targeted at cognition (primarily short-term memory and attention).Also within the Jintronix VR platform were exercises, demonstrated by an avatar, designed to improve strength, flexibility, and aerobic conditioning	**Who provided:** physiotherapist<br><br>**Where:** therapist unknown location, patient in their own homes.	full telerehab asynchronous VR, phone call	They were instructed to do their VR program five times a week for 6 weeks. Once the games became too easy, the difficulty was increased to require greater movement speed, distance, and/or accuracy.	**Tailoring**: A customized VR exercise program was created for each participant by the intervention physiotherapist<br>**Modifications:** N/A<br>**Adherence**: Participants completed 99% of the prescribed exercise	unknown number of adverse events/11 participants<br>severity: minor<br>type: physical, non-physical<br>relatedness: N/A<br>description: increased pain due to flareups in pre-existing knee and shoulder conditions, fatigue, concerns about possibility of injury or falls
Seidler et al. [87]	**Publication Year**: 2017<br>**Pre-Covid**<br>**Sample Size**: intervention = 10, control = 10, total participants = 20<br>**Mean age:** 68.1<br>**Sex:** 60% women<br>**Country of origin:** USA<br>**Population**: Parkinson's disease	quasi	investigate feasibility of a telerehabilitation approach to group tango instruction for people with PD and to compare key outcomes from a class taught virtually to a class with in-person instruction.	Acrobat Connect, Latitude E6410 laptops, PTZ pro webcams, XGA projectors, YVC-1000 speaker and microphone systems	tango	**Who provided:** instructor was a graduate student in physical therapy with 15 years of dance experience, who completed a course approved by the American Council on Exercise for adapted tango plus additional individual instruction with the developer<br><br>**Where:** instructor taught from laboratory space on the Washington University School of Medicine in St. Louis campus, participants at community location of the American Parkinson Disease Association	full telerehab synchronous video conferencing	hour-long, twice-weekly dance classes during the same twelve-week period. 10 min warm-up, 20 min of rhythm and partner work, 25 min of practice with a new step plus integration with previously learned patterns and a 2–3 min cool-down	**Tailoring:** N/A<br>**Modifications:** N/A<br>**Adherence**: Participant retention was 85% in both groups. Attendance was 87% in the Telerehab group	0 adverse events/10 participants<br>0 adverse events/240 sessions<br>severity: N/A<br>type: N/A<br>relatedness: N/A<br>description: N/A

Schlichting et al. [89]	**Publication Year**: 2022<br>**Post-Covid**<br>**Sample Size**: total participants = 10<br>**Mean age**: 26.2 weeks chronological age<br>**Sex**: 60% women<br>**Country of origin:** Brazil<br>**Population:** Infants at Risk of Cerebral Palsy	mixed methods	verify the effects of a telerehabilitation program for infants at high risk for Cerebral Palsy (CP) during the COVID-19 pandemic	diary, standardized instructions in written form and voice messages via WhatsApp, video calls, manual of each assessment tool, doll to demonstrate positions and movements	four main components: activities to enhance motor development, environmental enrichment, guidance about the infant positioning during the day, and educational strategies for the caregivers.	**Who provided:** pediatric physiotherapists<br><br>**Where:** therapist unknown location, patient in their own homes.	full telerehab synchronous video conferencing	Once a week, the caregiver applied the intervention to the infant under the supervision of one researcher. The supervised session lasted about 60 minutes. Researchers called the caregivers by video, explained the goal-directed activities, and observed the caregivers applying each goal Four times a week, the caregivers provided the rehabilitation program at home without supervision. The non-supervised sessions lasted around 20 minutes per session. The program had a total duration of 12 weeks. The expected total dosage of the rehabilitation program was 1680 minutes (60 minutes, once a week, of supervised sessions + 20 minutes, four times a week, of unsupervised sessions; for a total of 12 weeks, total dose 28 hours)	**Tailoring:** activities were chosen based on goals determined by the caregivers, leading, focusing on functional activities according to the infant's age and level of ability. Each activity was contextualized to the environment and incremented according to the ability and the progression of each infant<br>**Modifications:** N/A<br>**Adherence:** Adherence to the telecare program was high (90%).	0 adverse events/10 participants<br>0 adverse events/120 sessions<br>severity: N/A<br>type: N/A<br>relatedness: N/A<br>description: N/A
Sari et al. [92]	**Publication Year:** 2023<br>**Post Covid**<br>**Sample Size:** total participants = 30<br>**Mean age**: 71.5<br>**Sex**: 63.3% women<br>**Country of origin**: Indonesia<br>**Population:** dementia	quasi	evaluate the feasibility of a telehealth home-based exercise program for older people with dementia living in Indonesia with support from their informal carers.	computer/tablet and internet connection, Zoom software, stable chair or bench, an exercise booklet (sent via email, exercises in simple terms and with pictures), YouTube video links of the prescribed exercises,	warm up, balance, resistance and walking exercises. exercise program was modified from the Otago exercise program (an exercise program for the prevention of falls)	**Who provided:** physiotherapist<br><br>**Where:** therapist unknown location, patient in their own homes.	full telerehab synchronous video conferencing	12 weeks, with four exercise sessions (online visits, in real-time) delivered via video.<br><br>The dyads were instructed to continue the home exercise intervention for 5 days per week, 20–30 min each day in between the scheduled online visits. aiming for moderate-intensity exercise level, in terms of exercise demands and difficulty for each participant. The four online (visit) sessions were scheduled at weeks 1, 2, 6 and 10 during the 12-week intervention phase	**Tailoring:** program was individualized and tailored. The exercise level was gradually progressed over time for participants as they improved their ability to complete the program's current exercises<br>**Modifications:** N/A<br>**Adherence:** Median adherence was 84.1% during the 12-week intervention, and 66.7% in the self-maintenance period	0 adverse events/30 participants<br>0 adverse events/120 sessions<br>severity: N/A<br>type: N/A<br>relatedness: N/A<br>description: N/A
Saitoh et al. [27]	**Publication Year**: 2022<br>**Post-Covid**<br>**Sample Size:** intervention = 6, control = 5, total participants = 11<br>**Mean age:** 74<br>**Sex:** 33% women<br>**Country of origin:** Japan<br>**Population**: cardiac disease	RCT	investigate the feasibility on remote CR support program (Remote-CR) in older patients with cardiac disease.	tablet computer (iPad), remote real-time telemedicine system with videoconferencing capabilities (Nipro HeartLine), original exercise video file detailing the aerobic, resistance, balance, and flexibility exercises, pedometer, logbook, ECG, pulse oximeter, electronic sphygmomanometer	cardiac rehab- aerobic, resistance, balance, and flexibility exercises	**Who provided:** physiotherapist<br><br>**Where:** therapist unknown location, patient in their own homes.	full telerehab asynchronous video conferencing: using video calling for feedback, but doing exercises during unsupervised time	4 weeks. Physiotherapists contacted patients via a remote real-time monitoring system weekly. In addition, physical therapists checked handwritten logbook including exercise diary, pedometer-assessed daily steps, and disease management status through the display of videoconference, and provided feedback to the patients<br><br>asked to continue unsupervised exercises for three or more times a week for 4 weeks. The exercise program was 20 - 30 min at Borg scale of 11 - 13.	**Tailoring**: N/A<br>**Modifications:** N/A<br>**Adherence:** only about 30% of the patients performed exercise at home at least three times a week using an exercise video.	0 adverse events/6 participants<br>0 adverse events/72 sessions<br>severity: N/A<br>type: N/A<br>relatedness: N/A<br>description: N/A

Rosenbek Minet et al. [52]	**Publication Year:** 2015<br>**Pre-Covid**<br>**Sample Size:** total participants = 37<br>**Mean age:** 69.2<br>**Sex:** 86% women<br>**Country of origin:** Denmark<br>**Population**: severe COPD	quasi	assess the feasibility of an individualized home-based training and counselling programme via video conference to patients with severe COPD after hospitalization including assessment of safety, clinical outcomes, patients' perceptions, organisational aspects and economic aspects.	telemedicine videoconferencing equipment was designed to look like a briefcase and was known as the "Patient Briefcase". contained a screen, microphone, an on/off switch and a volume control. pulse oximeter	Thoracic mobilization exercises, cardio training, strength training, and breathing exercises.<br><br>The training involved: warming up, consisting of thoracic mobilization exercises, exercises for upper extremities, lower extremities and neck/shoulder<br>- cardio training, consisting of swing exercises, walking on the spot, seated exercises and, if possible, a stair workout<br>- strength training, consisting of elastic exercises for upper and lower extremities, standing squats and stand and sit chair exercises.<br>- Breathing exercises such as pursed lip breathing and diaphragmatic. breathing were used between the exercises to alleviate intercostal breathing.<br>In addition, patients were asked to train on their own on days when there was no telemedicine session with a physiotherapist.<br><br>There were 1–2 sessions with the occupational therapist, which consisted of training and counselling on energy conservation techniques.	**Who provided:** physiotherapist<br><br>**Where**: therapist unknown location, patient in their own homes.	full telerehab synchronous video conferencing	3 weekly sessions, lasting 30–45 minutes, over a 3 week period, i.e. a total of 9 supervised sessions. thoracic mobilization exercises, cardio training with an intended intensity of 60%-90% of max capacity, strength training with an intended intensity of 60% of 1 Repetitions Maximum (RM) and breathing exercises.<br><br>The training involved: 5–10 minutes warming up; 10–15 minutes cardio training; 10–15 minutes strength training. Breathing exercises between the exercises to alleviate intercostal breathing. The training intensity was continuously progressed. In addition, patients were asked to train on their own on days when there was no telemedicine session with a physiotherapist.<br><br>There were 1–2 sessions with the occupational therapist. The first session was 60 minutes long, delivered in the second week of the intervention. If required, a second session of 30 minutes was given in the third week	**Tailoring**: individualized home-based training and counselling programme<br>**Modifications:** N/A<br>**Adherence**: Thirty seven (74%) participants completed the programme	0 adverse events/37 participants<br>0 adverse events/333 sessions<br>severity: N/A<br>type: N/A<br>relatedness: N/A<br>description: N/A
Rosen et al. [47]	**Publication Year:** 2020<br>**Post-Covid**<br>**Sample Size**: total participants = 12<br>**Mean age:** 52.8<br>**Sex:** 9% women<br>**Country of origin:** USA<br>**Population**: COVID	observational	ascertain how the inpatient telerehabilitation program was implemented and whether it was a viable option for COVID-19 patients.	Zoom for video communications, iPads, external telemeter and oxygen saturation (SpO2) monitors	COVID rehab - exercises based on muscles important to maintain functional mobility such as ambulation and sit-to-stand transfers, lower extremity muscle groups including gluteals and quadriceps were targeted during supine, seated, and standing exercises [2, 24, 25]. Upper extremity exercises were provided to aid in respiratory function, posture, and functional mobility	**Who provided:** physiotherapist<br><br>**Where:** therapists in hospital, patients in separate hospital room	full telerehab synchronous video conferencing	The average patient length of stay was 9.1 days, with telerehabilitation evaluation occurring on average 5 days after admission.<br><br>performed one set of five to ten repetitions of each exercise based on rate of perceived exertion (RPE)with physical therapist supervision via telerehabilitation.<br><br>Patients were encouraged to complete the same set of exercises an additional two times, without supervision throughout the day, for a total of three sets per day.	**Tailoring:** provided individualized therapeutic exercises<br>**Modifications:** N/A<br>**Adherence:** N/A	0 adverse events/12 participants<br>0 adverse events/108 sessions<br>severity: N/A<br>type: N/A<br>relatedness: N/A<br>description: N/A

Qiu et al. [67]	**Publication Year:** 2020<br>**Pre-Covid**<br>**Sample Size**: total participants = 15<br>**Mean age**: 56.67<br>**Sex:** 13.3% women<br>**Country of origin:** USA<br>**Population:** chronic stroke	quasi	present a system designed and implemented in our lab: the Home based Virtual Rehabilitation System (HoVRS).	Home based Virtual Rehabilitation System (HoVRS) cross-platform virtual reality training application, Leap Motion Controller (LMC) - infrared tracking device consisting of three infrared LEDs and two cameras. multiple engaging games designed to train the hand and arm using commercial gaming mechanics to optimize players' motivation to perform these activities for long periods of time, monitoring and archiving software that will allow clinicians to design custom rehabilitation interventions, track a patient's progress, and modify a patient's rehabilitation program, in-person or remotely, and (4) a secure wireless data connector web portal	intense upper extremity rehabilitation - subset of 5 games (Maze, Wrist Flying, Finger Flying, Car, Fruit Catch) out of the 12-game library, at least one from each category (Elbow-Shoulder, Wrist, Hand, Whole Arm)	**Who provided:** physiotherapist and engineer<br><br>**Where:** therapist unknown location, patient in their own homes.	hybrid telerehab hybrid (synchronous and asynchronous) VR, video conferencing	HoVRS was placed in subjects' homes for 3 months. Each weekday, subjects were encouraged to play at least 3 rehabilitation activities for a total minimum of 15 min.	**Tailoring**: performed a series of calibrations that were used to customize the games to accommodate the movement abilities of each subject.<br>**Modifications**: N/A<br>**Adherence:** 27% had 100% adherence, 47% had >80% adherence	0 adverse events/15 participants<br>0 adverse events/707 sessions<br>severity: N/A<br>type: N/A<br>relatedness: N/A<br>description: N/A
Plaza et al. [97]	**Publication Year:** 2023<br>**Pre-Covid**<br>**Sample Size:** intervention = 23, control = 22, total participants = 45<br>**Mean age:** 48.4<br>**Sex:** 30.4% women<br>**Country of origin:** Australia<br>**Population:** burn injury	RCT	determine if exercise programs delivered via HBT were as effective as in-person (IP) programs with respect to clinical outcomes and participant and therapist satisfaction.	eHAB® - an online, web-based platform and clinically validated telerehabilitation system	burn specific stretching and strengthening exercises for each body area underlying the burn injury.<br><br>education specific to their burn injury and a home exercise program to continue independently with encouragement via resources including exercise sheets, a burn exercise DVD produced by the RBWH Physiotherapy Department entitled "Stretching your Limits" and a summary of the treatment plan.	**Who provided:** physiotherapist<br><br>**Where:** therapist unknown location, patient in their own homes.	full telerehab synchronous video conferencing	6-week exercise program, minimum frequency of one session per fortnight and a maximum of two sessions per week. could therefore access between 3 and 12 physical therapy sessions in the six-week intervention period. Exercise sessions were 30–60 min in duration	**Tailoring**: N/A<br>**Modifications**: N/A<br>**Adherence:** total 76 HBT appointments completed	0 adverse events/23 participants<br>0 adverse events/76 sessions<br>severity: N/A<br>type: N/A<br>relatedness: N/A<br>description: N/A
Piraux et al. [104]	**Publication Year:** 2019<br>**Pre-Covid**<br>**Sample Size:** intervention = 9, control = 8, total participants = 17<br>**Mean age:** 47.2<br>**Sex:** 33.33% women<br>**Country of origin**: Belgium<br>**Population:** HIV	RCT	investigated feasibility and preliminary effects of telerehabilitation for people living with HIV (PLWH) and taking antiretroviral therapy.	website with all information needed to perform the resistance training. For each exercise, initial position, execution of the movement, duration of exercise, number of repetitions, and rest time were described and illustrated by a video.	combined aerobic and resistance training	**Who provided:** physiotherapists<br><br>**Where:** therapist unknown location, patients in public fitness center	full telerehab asynchronous telephone	exercised for 6 weeks (3 sessions/week). Aerobic training (50 min) consisted of a 30-min cycle ergometer (10-min warm-up period 1 20 min of interval training; 1:2 at 80% and 60% maximum heart rate), a 10-min walk on a treadmill (at the speed obtained at the 6-min walk test [6MWT]), and 10-min on a rowing machine (between 70% and 80% maximum heart rate). Resistance training (25 min) of large muscle groups (back, quadriceps, and chest) was performed with three sets of 10 repetitions at 60% to 80% of the participant's estimated 1 repetition maximum.	**Tailoring:** intensity based on HR and 6MWT and 1-RM<br>**Modifications**: N/A<br>**Adherence:** Recruitment and retention rates were 93% and 69%	0 adverse events/9 participants<br>0 adverse events/162 sessions<br>severity: N/A<br>type: N/A<br>relatedness: N/A<br>description: N/A

Piraux et al. [72]	**Publication Year**: 2020<br>**Pre-Covid**<br>**Sample Size:** total participants = 22<br>**Mean age:** 61.7<br>**Sex:** 30.4% women<br>**Country of origin**: Belgium<br>**Population:** Esophagogastric Cancer	quasi	assess the feasibility and the preliminary effects of a "tele-prehabilitation" program in esophagogastric cancer patients requiring surgery	online tele-prehabilitation platform - Virtuagym© fitness application. On the account, the patient found an exercise schedule, a description of each exercise session and a tab to send an e-mail to the physiotherapist. Website was composed of a set of exercise videos and was available on computer, tablet or mobile phone.<br><br>heart rate monitor<br>inspiratory threshold-loading device	cancer prehabilitation, aerobic and resistance training and inspiratory muscle strengthening. Aerobic - Patients chose an activity such as walking, cycling or rowing according to their preferences and capabilities.	**Who provided:** physiotherapist<br><br>**Where:** therapist unknown location, patient in their own homes.	Hybrid telerehab asynchronous telephone, email	started 2 or 4 weeks before surgery, depending on the treatment, and ended the day before surgery. It included aerobic and resistance training 3 times a week and inspiratory muscle strengthening 5 times a week.<br><br>Aerobic training - 30 min at an intensity of 65–74% of maximum heart rate (HRmax = 220□age) controlled with a heart rate monitor. The progressive resistance training consisted of 8–10 weight-bearing exercises targeting major muscle groups of the trunk, upper limbs and lower limbs for an average of 30 min. Subjects performed 1 to 4 sets of 8–12 repetitions at a moderate intensity, rated as 4–6 on rated perceived exertion (range 0–10 on the modified Borg scale). The volume of resistance exercises varied over the course of the sessions. Inspiratory muscle training (IMT) was undertaken with an inspiratory threshold-loading device for 15 min. Patients were asked to maintain a respiratory rate of 15 to 20 breaths per minute. The inspiratory resistance of the IMT started at 30% of the maximal inspiratory mouth pressure measured at baseline with a lung function test and was gradually increased by 5% if the rate of perceived exertion on the Borg scale remained under 5	**Tailoring:** program was personalized based on the patient's initial functional capacity.<br>**Modifications**: N/A<br>**Adherence**: Recruitment and retention rates were both 96%. Attendances to aerobic and resistance sessions and inspiratory muscle training were 77% and 68%, respectively	0 adverse events/182 sessions<br>0 adverse events/22 participants<br>severity: N/A<br>type: N/A<br>relatedness: N/A<br>description: N/A
Piotrowicz et al. [28]	**Publication Year**: 2019<br>**Pre-Covid**<br>**Sample Size:** intervention group = 425, control group = 425, total = 850<br>**Mean age:** 62.6<br>**Sex:** 11.3% women<br>**Country of origin**: Poland<br>**Population:** heart failure	RCT	assess whether potential improvements in quality-of-life outcomes after a 9-week HCTR intervention in patients with heart failure translate into improvement in clinical outcomes during extended 12 to 24 months of follow-up, compared with usual care	monitoring system included (1) a special remote device for supervised exercise training monitored with tele-ECG (also called a telerehabilitation set; Pro PlusCompany), which consists of an EHO mini device, blood pressure device, and body-weight scale; (2) a data transmission set via a mobile telephone; and (3) a monitoring center capable of receiving and storing patients' medical data. The EHO mini device is able to record ECG data from 3 precordial leads and transmit them via a mobile telephone network to the monitoring center. The device has training sessions preprogrammed individually for each patient (with defined exercise duration, breaks, and timing of ECG recording	Aerobic endurance training, Respiratory muscle training, Resistance and strength training	**Who provided:** medical team (physicians, physiotherapists, nurses, and a psychologist)<br><br>**Where:** therapist unknown location, patient in their own homes.	Hybrid telerehab asynchronous EHO mini device, phone	9-week HCTR program consisting of 2 stages: an initial stage (1 week) conducted in hospital and a basic stage (8 weeks) of home-based HCTR 5 times weekly.	**Tailoring:** tailored patient's rehabilitation program<br>**Modifications:** N/A<br>**Adherence:** The adherence to HTCR was very high during the 9-week training period. There were 350 patients who were adherent (88.4%), 39 who were partially adherent (9.8%), and 7 patients who were nonadherent (1.8%).	63 adverse events/425 participants<br>63 adverse events/17000 sessions<br>severity: not serious<br>type: physical<br>relatedness: 51 unrelated, 12 undefined relatedness<br>description: dyspnea/tachypnea >30 breaths/min, syncope, weight gain of at least 1.8kg during 1-3 days, complex ventricular arrhythmia

Piotrowicz et al. [33]	**Publication Year:** 2014<br>**Pre-Covid**<br>**Sample Size:** intervention group = 365, total participants = 365<br>**Mean age:** 58.3<br>**Sex:** 16% women<br>**Country of origin**: Poland<br>**Population:** Cardiovascular disease	quasi	evaluate a wide implementation and feasibility of home-based cardiac telerehabilitation (HTCR) in patients suffering from CVD and to assess its safety, patients' acceptance of and adherence to HTCR	remote- controlled equipment for tele-electrocardiogram (ECG)-monitoring and supervised ET, which consisted of an EHO mini device and blood pressure measuring. The device allowed to record ECG data from 3 pre- -cordial leads and transmit them via mobile phone network (using an integrated mobile phone) to the monitoring center. The mobile phone was also used for voice communication. The telemonitoring system had details of the training sessions pre-programmed for each patient (defined exercise duration, breaks, timing of ECG recording). The times of automatic ECG recording were pre-set and coordinated with the ET. The planned training sessions were executed with a device indicating what should be done via sound and light signals.	cardiac rehab - two stages: an initial stage — conducted within an outpatient center (3 days), and a basic stage — conducted at home (4 weeks). The goals of the initial stage were: a baseline clinical examination, optimization of treatment, education, individual planning of ET, performing a few (3–6) monitored educational training sessions, and psychological assessment.<br><br>The basic stage, which was conducted at home and consisted of two parts, was performed prior to each training session: the first part — the training consent procedure was required for a patient to access each training session, and the second part — the training session. The training consent procedure included: telephone conversation with a nurse during which the patient answered questions about their present condition, symptoms, medications taken and sent rest ECG and blood pressure data.	**Who provided:** nurse, physician<br><br>**Where:** therapist unknown location, patient in their own homes.	Hybrid telerehab asynchronous EHO mini device, phone	The target training HR was 60–80% of the HR reserve. Each training session consisted of three parts: (1) a warm up lasting for 5–10 min, consisting of breathing, light resistance exercises and calisthenics; (2) an aerobic endurance training based on different forms i.e. either walking or Nordic walking or cycloergometer training for 30 min each; (3) a 5-min cooling down period. Patients trained 5 times a week.<br>4 week	**Tailoring:** Exercise training was planned individually for each patient in line with the published guidelines<br>**Modifications:** N/A<br>**Adherence:** There were only 0.8% non-adherent patients.	0 adverse events/365 participants<br>0 adverse events/7300 sessions<br>severity: N/A<br>type: N/A<br>relatedness: N/A<br>description: N/A
Pinto et al. [29]	**Publication Year**: 2022<br>**Post-Covid**<br>**Sample Size:** total participants = 116<br>**Mean age:** 62.6<br>**Sex**: 22.1% women<br>**Country of origin**: Portugal<br>**Population:** Cardiovascular disease	quasi	to assess physical activity (PA) levels after completing a home-based digital CR program.	consisted of recorded videos and real-time online exercise training sessions (via computer, tablet or smartphone with internet access).<br>Body weight and/or household objects (e.g. books, bottles of water, packs of rice, etc.). a pictorial exercise training guidebook was available.<br>personal health diary	Exercise training - Sessions included warm-up, circuit training and cool-down). Instructed to perform eight different exercises (two upper limbs, two lower limbs, two core stability, two balance).<br><br>Also had patient assessment, risk assessment, physical activity counseling, nutritional counseling, weight control management, blood pressure management, diabetes management, smoking cessation, psychosocial management, education.	**Who provided:** exercise physiologists and physiotherapists.<br><br>**Where:** therapist unknown location, patient in their own homes.	full telerehab synchronous computer/ tablet/ smartphone (video conferencing?)	supervised: Once a month, real-time CR exercise sessions lasting 60 min. 3 month program.<br>unsupervised: Participants were recommended to do each session three times a week, for 60 min (10 min warm-up, 40 min circuit training and 10 min cool-down), performing eight different exercises (two upper limbs, two lower limbs, two core stability, two balance). Each exercise had three levels of progression (easy, moderate, difficult) with two or three sets of 10-15 repetitions, and all exercises were done with body weight and/or household objects (e.g. books, bottles of water, packs of rice, etc.). The aim, using the Borg Rating of Perceived Exertion scale (6-20), was to reach moderate intensity, i.e. a score of 11-14.	**Tailoring**: N/A<br>**Modifications**: N/A<br>**Adherence:** Nearly half (46.9%) of the participants did at least one online exercise training session per week. Among those who did training sessions, 58% did two or three times per week, 27% once per week and 15% more than four times per week.	1 adverse event/116 participants<br>1 adverse event/348 sessions<br>severity: minor<br>type: physical<br>relatedness: N/A<br>description: sprained ankle

Pfister et al. [99]	**Publication Year**: 2021<br>**Pre-Covid**<br>**Sample Size:** total participants = 14<br>**Mean age**: 59<br>**Sex**: 50% women<br>**Country of origin:** Switzerland<br>**Population**: inflammatory myopathies	quasi	evaluate the feasibility of our newly developed blended therapy approach, combining a tablet-based exercise application (app) with face-to-face physiotherapy sessions in patients with IM, and [2] to evaluate potential impacts on muscle strength, muscle function, activity limitation, disability and healthrelated quality of life (HRQOL).	tablet-based exercise application (app), training diary, phone	open and closed chain exercises for the most commonly affected muscle groups in IM. Resistance training	**Who provided:** physiotherapist<br><br>**Where**: therapist unknown location, patient in their own homes.	hybrid telerehab asynchronous mobile application	12 week program, 6 to 8 exercises. 2–3 sets with 8–15 repetitions at perceived intensities between levels 13 and 17 on the 6 to 20 points Borg scale. if the participants tolerated these intensities without pain and signs of an inflammation, the intensity was increased to 15 to 17.	**Tailoring:** individually tailored resistance training program based on their examination<br>**Modifications**: N/A<br>**Adherence**: Mean adherence to exercise program was 84%	1 adverse event/14 participants<br>severity: non-severe<br>type: physical<br>relatedness: unrelated<br>description: renal colic
Peng et al. [34]	**Publication Year**: 2018<br>**Pre-Covid**<br>**Sample Size**: intervention group = 49, control group = 49, total participants = 98 participants.<br>**Mean age:** 66.3<br>**Sex:** 40.8% women<br>**Country of origin**: China<br>**Population:** heart failure	RCT	examine the effect of our telehealth exercise training program on health outcomes in patients with HF in China	printed brochure, online webcam communication and supervision using QQ and Wechat software, instant messaging,	cardiac rehab - first stage - endurance exercises<br>second stage - resistance and muscular strengthening exercises	**Who provided:** multi-disciplinary team consisting of physiotherapists (exercise training), cardiac nurses (follow-up and self-care instruction), and psychiatric nurses (psychological instruction).<br><br>**Where:** therapist unknown location, patient in their own home.	full telerehab synchronous video conferencing	8-week home-based telehealth exercise training program, including 32 exercise training sessions, with regular telephone or instant messaging follow-ups and consultation. The 2-month exercise training program consisted of 2 stages:<br>the first stage (1–4 weeks) was focused on endurance exercises, with 3 20-minute sessions per week. The training modalities included walking and jogging. The patients received a total of 12 20-minute sessions of exercise training in the first stage, with 3 sessions per week. The second stage (5–8 weeks) included resistance and muscular strengthening exercises in 5 30-minute sessions per week. The patients performed endurance exercises before progressing to resistance exercises. The training modalities included walking, jogging, and calisthenics for muscular training. The muscular strengthening exercises included multiple weight-bearing calisthenics, such as single-leg squats, deep squats and partial squats. Resistance exercises were performed using the elastic band. The patients received a total of 20 30-minute sessions of exercise training in the second stage, with five sessions per week.<br><br>The target training HR was 40% to 70% of the HR reserve plus the resting HR	**Tailoring**: based on HR - The target training HR was 40% to 70% of the HR reserve plus the resting HR<br>**Modifications:** N/A<br>**Adherence:** the attrition rates of the experimental and control groups at 4 months post-test were 14.3% and 16.3%, respectively.	0 adverse events/49 participants<br>0 adverse events/1568 sessions<br>severity: N/A<br>type: N/A<br>relatedness: N/A<br>description: N/A

Paul et al. [91]	**Publication Year:** 2019<br>**Pre-Covid**<br>**Sample Size:** intervention group = 45, control group = 45, total participants = 90<br>**Mean age**: 55.6<br>**Sex:** 71.1% women<br>**Country of origin:** UK<br>**Population:** multiple sclerosis	RCT	examine the feasibility of a trial to evaluate web-based physiotherapy compared to a standard home exercise programme in people with multiple sclerosis	www.webbasedphysio.com, website containing exercises (videos, text and audio description) and disease-specific advice and education	cardiovascular, strengthening and balance exercises, as<br>well as warm up, cool down and stretching	**Who provided:** physiotherapist<br><br>**Where:** therapist unknown location, patient in their own homes.	full telerehab asynchronous website, email	Participants were advised to undertake their physiotherapy programme twice per week for six months (2 × 26 weeks = 52 diary entries).	**Tailoring:** different levels of difficulty and a prescribed number of sets/repetitions individualized to<br>meet the participants' needs.<br>**Modifications:** N/A<br>**Adherence:** Adherence was 40%-63% in the intervention group.	60 adverse events/45 participants<br>60 adverse events/2340 sessions<br>severity: N/A<br>type: physical<br>relatedness: unrelated<br>description: falls, skin reactions
Parraguez et al. [75]	**Publication Year:** 2023<br>**Post-Covid**<br>**Sample Size:** total participants = 72<br>**Mean age:** 69.3<br>**Sex:** 48.6% women<br>**Country of origin:** Chile<br>**Population**: cancer - patients who are candidates for elective cancer surgery	observational	describe the implementation of a teleprehabilitation program during the COVID-19 pandemic for patients who are candidates for elective cancer surgery in a low-income Chilean public hospital. Secondarily, describe the perspectives and satisfaction of patients with the program	smartphone, Zoom platform, work kit which contained: a set of elastic bands of yellow, red and blue colors, a volumetric incentive, (Coach 2®Incentive Spirometer/22–4000); a printed guide with support materials and a booklet for recording daily activities carried out, including basic daily activities	breathing, flexibility, strength, proprioceptive, balance and aerobic exercises, based on standardized guidelines	**Who provided:** physiotherapist<br><br>**Where:** therapist unknown location, patient in their own homes.	hybrid telerehab synchronous video conferencing, phone	8 sessions approximately 45 min, 2–3 times a week. The first session was face-to-face and the remaining<br>7 sessions were executed remotely.	**Tailoring:** Mixed exercise plan: This considered modifiable clinical risk factors, perceived needs, and was personalized based on the baseline condition of each patient evaluated in the first session.<br>**Modifications:** N/A<br>**Adherence:** 99.3% recruitment, and a retention rate of 46.7%,	0 adverse events/72 participants<br>0 adverse events/576 sessions<br>severity: N/A<br>type: N/A<br>relatedness: N/A<br>description: N/A
Palmcrantz et al. [68]	**Publication Year:** 2017<br>**Pre-Covid**<br>**Sample Size**: total participants = 15<br>**Mean age**: 66<br>**Sex:** 46.67% women<br>**Country of origin:** Sweden<br>**Population**: stroke	qualitative	explore the feasibility and safety of using the DISKO-tool, customized for interactive stroke rehabilitation in the home setting, in different rehabilitation phases after stroke	VR The DISKO-tool integrated a video communication system, remote patient monitoring, and evaluation of stroke-specific individualized movement controlled exercises using a motion capture system.<br>The set-up included a big screen, Kinect sensor, separate sound unit including speakers and microphone and computer connected to internet. Technical components in the clinic included: a computer connected to internet, web-camera and headset. Manual provided.	six exercises focusing on balance and arm movements through weight shifting, stepping and reaching.	**Who provided:** physiotherapist<br><br>**Where:** therapist unknown location, patient in their own homes.	full telerehab hybrid (synchronous and asynchronous) video conferencing (VR study)	The intervention lasted for 3 weeks, with 15 planned training sessions (including training during follow-ups by video communication with the physiotherapist).<br>6 supervised, 9 unsupervised.	**Tailoring:** Based on the initial assessments of functioning and disability and the patients' performance of the test exercises.<br>**Modifications:** N/A<br>**Adherence:** Fourteen of the 15 included patients finalized the three-week training period.	0 adverse events/15 participants<br>0 adverse events/195 sessions<br>severity: N/A<br>type: N/A<br>relatedness: N/A<br>description: N/A

Ozturk et al. [102]	**Publication Year**: 2022<br>**Post-Covid**<br>**Sample Size:** intervention group = 21, control group = 20, total participants = 41<br>**Mean age:** 41.05<br>**Sex:** 57% women<br>**Country of origin**: Turkey<br>**Population:** overweight and obese	RCT	examine the effects of exercise training through telerehabilitation applied during COVID-19 isolation period on overweight and obese individuals on physical fitness and quality of life.	The interviews took place synchronously (live) from a convenient computer program that allowed remote exercise training, mat	warm-up and cooling exercises consisting of flexibility exercises for the lower and upper extremities and torso. Mat exercises, which included a total of 21 body stabilizers performed in five different positions (back, side down, knee up, sitting and face up). Core stabilization exercises were performed to provide abdominal hallowing. 5 breathing exercises including diaphragmatic, chest breathing exercises and thoracic expansion exercises.	**Who provided:** physiotherapist<br><br>**Where:** therapist unknown location, patient in their own homes.	full telerehab synchronous video conferencing	3 sessions per week, an average of 45 min, for a total of 6 weeks. Mat exercises, which included a total of 21 body stabilizers performed in five different positions (back, side down, knee up, sitting and face up) were initially performed 10 repetitions.<br>The degree of difficulty was increased when individuals performed 10 repetitions (approximately every 2 weeks), maintaining spinal smoothness in each movement	**Tailoring:** N/A<br>**Modifications:** N/A<br>**Adherence**: At the end of the 6-week study periods all participants were able to complete the study protocol.	0 adverse events/21 participants<br>0 adverse events/378 sessions<br>severity: N/A<br>type: N/A<br>relatedness: N/A<br>description: N/A
Øra et al. [71]	**Publication Year:** 2020<br>**Pre-Covid**<br>**Sample Size**: intervention group = 32, control group = 30, total participants = 62<br>**Mean age:** 64.7<br>**Sex:** 40.6% women<br>**Country of origin**: Norway<br>**Population:** patients with post-stroke aphasia	RCT	Pilot a definitive randomized controlled trial of speech-language telerehabilitation in poststroke aphasia in addition to usual care with regard to recruitment, drop-outs, and language effects.	Norwegian translation of the Newcastle University Aphasia Therapy Resources and a computer training program targeting all language modalities called Lexia. We also used "Sareptas afasikrukke," a collection of Norwegian tasks comprising individual aphasia exercises training all modalities, for example, oral and written naming, reading sentences and text. In addition, text, maps, and pictures from the Internet were used as resources in therapy sessions.<br><br>videoconference software Cisco, laptops, software LogMeIn, external speaker and a wide-angle web camera	mixed approach to design an intervention aiming to enhance functional expressive communication. This included different impairment- based methods like functional-orientated<br>therapy to phonological, semantic, cognitive-linguistic, and cognitive-neuropsychological approaches.	**Who provided:** speech-language pathologist<br><br>**Where:** therapist unknown location, participants at their home or admitted to secondary rehabilitation centers.	full telerehab synchronous video conferencing	5 hours a week, over four consecutive weeks (total dose of 20 h of therapy). Participants with >16 sessions over 32 days will be considered to be per protocol. Participants usually received 60 minutes of speech-language therapy via videoconference per day, five days per week. In some cases, more prolonged therapy time (70–120 minutes) was delivered over fewer days per week, to adjust to the participant's timetable and other planned activities	**Tailoring:** The therapy was tailored to the individual participant's language impairment, needs and goals in all language modalities (reading, writing, spoken language, and auditory comprehension).<br>**Modifications:** N/A<br>**Adherence:** N/A	0 adverse events/30 participants<br>0 adverse events/480 sessions<br>severity: N/A<br>type: N/A<br>relatedness: N/A<br>description: N/A
Moriichi et al. [101]	**Publication Year**: 2022<br>**Post-Covid**<br>**Sample Size:** total participants = 9<br>**Mean age:** 84.7<br>**Sex:** 88.9% women<br>**Country of origin:** Japan<br>**Population:** elderly	quasi	proposed a novel procedure<br>for fall-preventive telerehabilitation for elderly people, which combined a physical therapist (PT)-assisted and personalized telerehabilitation program with a caregiver-education program.	VidyoRoom HD230 software program, tablet or laptop, wall with a handrail, rehabilitation manual for caregivers	fall prevention rehab - six exercises (rising training, high knee training, straight leg raise training, side lying leg lift training, heel raise training and a knee straightening exercise), which mainly focused on muscular strength training of the lower limbs and the improvement of sitting balance.	**Who provided:** Specialists, including medical doctors (MDs) from the rehabilitation department, PTs and nurses<br><br>**Where**: therapist is located in Telemedicine Center of Asahikawa Medical University, patients in elderly group homes and nursing homes	full telerehab synchronous video conferencing	At the first session, the rehabilitation session was observed by MDs, nurses and PTs using the telerehabilitation system. The interval between rehabilitation sessions depended on the participants' rehabilitation program and condition.<br>All participants underwent telerehabilitation five times in three months (first time, one week, one month, two months and three months later, respectively).<br>The participants were required to continue the rehabilitation two or three times per week by themselves with caregiver's support in their nursing home.	**Tailoring**: created a personalized telerehabilitation plan for each patient.<br>**Modifications:** N/A<br>**Adherence**: N/A	0 adverse events/9 participants<br>0 adverse events/45 sessions<br>severity: N/A<br>type: N/A<br>relatedness: N/A<br>description: N/A

Mayer et al. [48]	**Publication Year**: 2021<br>**Post-Covid**<br>**Sample Size:** intervention group = 10, control group = 22, total participants = 32<br>**Mean age:** 55<br>**Sex:** 50% women<br>**Country of origin:** USA<br>**Population:** Adult patients surviving acute respiratory failure due to critical COVID	quasi	Examine the safety and feasibility of a multimodal in-person or telehealth treatment program, administered in acute recovery phase for patients surviving critical coronavirus disease 2019	home pulse oximeters and automated blood pressure monitors, free weights, elastic bands, exercise diary	pulmonary rehab - aerobic, strength, breathing and mindfulness	**Who provided:** physiotherapist<br><br>**Where:** therapist unknown location, patient in their own homes.	full telerehab synchronous teleconference	8 weeks total. one or two supervised sessions per week (either in-person or supervised telehealth) and prescribed a supplemental unsupervised home program (3 or 4 d/wk).<br>Aerobic training: 15–30 minutes, targeted range of 4–6 on the modified rating of perceived exertion (RPE). Progressively increased when patients rated an activity less than 4 on the RPE, and their heart rate did not exceed the established target rates. Strength training: RPE with an initial rating of 5–6 of 10 performing 10–15 repetitions, progressed if a patient rated an exercise less than or equal to 4 of 10 on RPE and demonstrated ability to complete three sets of 15 repetitions. Supplemental home-exercise plan: Patients participated in a home-exercise plan, walk at home for at least 30 minutes per day with RPE less than or equal to 4, perform strengthening exercise (visual handouts provided) unsupervised 3–4 days per week, and to perform diaphragmatic breathing two to three times per day. Patients were instructed to log daily adherence to the HEP in an exercise diary.	**Tailoring:** Treatment dosing was pragmatic and multifactorial including patients' need and social determinant of health (transportation availability and access to caregiver).<br>**Modifications:** Breathing and mindfulness techniques were added to the program as a modification to original protocol during the first week of study enrollment; thus, all patients engaged in breathing and mindfulness techniques<br>**Adherence:** The group's attrition rate was equal (10% telehealth and 9% in-person).	9 adverse events/10 participants<br>9 adverse events/80 sessions<br>severity: minor, undefined<br>type: physical, non-physical<br>relatedness: related, unrelated<br>description: bouts of hypertension at rest and during exercise, rolling ankle, fall, increasing episodes of anxiety with chest tightness and palpitations
Martin et al. [49]	**Publication Year**: 2021<br>**Post-Covid**<br>**Sample Size**: intervention group = 14, control group = 13, total participants = 27<br>**Mean age**: 60.8<br>**Sex**: 21.43% women<br>**Country of origin**: Belgium<br>**Population**: severe COVID	observational	assess the functional exercise capacity of patients with severe COVID-19 and to evaluate the effect of a telerehabilitation program in the specific context of the COVID-19 pandemic.	videoconferencing platform (Teams, Microsoft), laptop, phone or tablet and a web camera, water bottles, chair	pulmonary rehabilitation program	**Who provided:** physiotherapist<br><br>**Where:** therapist unknown location, patient in their own homes.	full telerehab synchronous video conferencing	Patients performed home based exercises twice a week for 6 weeks. Each session was composed of 50 min including 30 min of endurance exercises followed by upper and lower body muscular strengthening. The intensity of the endurance training was fixed based on a 6-point score on the Borg scale. The upper and lower body muscles training was performed with materials available in the home environment of the participants (bottles of water and a chair). The participants were instructed to do 2–3 series of 8–12 repetitions for each exercise. After each session, patients received a file summarizing all the executed exercises. They were encouraged to perform unsupervised exercises 3 times a week, using the provided templates.	**Tailoring**: N/A<br>**Modifications:** N/A<br>**Adherence:** all the participating patients attended and completed the program	0 adverse events/14 participants<br>0 adverse events/168 sessions<br>severity: N/A<br>type: N/A<br>relatedness: N/A<br>description: N/A
