# Supplementary material for: Adverse events associated with the delivery of telerehabilitation across rehabilitation populations: A scoping review
Source: PLoS One. 2024 Nov 19;19(11):e0313440. doi: 10.1371/journal.pone.0313440 (PMC11575805; doi:10.1371/journal.pone.0313440)
Supplement: S6 Appendix — (DOCX) [file pone.0313440.s006.docx]

**S4. Appendix. Prisma-ScR Flow Diagram.**

**What is the extent of literature on adverse events when delivering telerehabilitation: A scoping review.**

Studies from databases/registers **(n = 11863)**

MEDLINE (R) ALL [Ovid] (n = 3,857)

Embase Classic + Embase [Ovid] (n = 4,214)

EBM Reviews – Cochrane Central Register of Controlled Trials (n = 1,798)

APA PsycInfo (n = 322)

CINAHL [Ebscohost] (n=1,672)

References from other sources **(n = 0)**

Citation searching (n = 0)

Grey literature (n = 0)

**Identification**

Studies included in review **(n = 81)**

Studies excluded **(n = 7346)**

Studies not retrieved **(n = 0)**

Studies assessed for eligibility **(n = 178)**

Studies sought for retrieval **(n = 178)**

Studies screened **(n = 7524)**

Studies excluded **(n = 97)**

Outcomes were not relevant (n = 24)

Ineligible intervention (n = 42)

Ineligible study design (n = 30)

Not written in English language (n = 1)

References removed **(n = 4339)**

Duplicates identified by Endnote (n = 3506)

Duplicates identified by Covidence (n = 833)

**Screening**

**Included**
