# Supplementary material for: Adverse events associated with the delivery of telerehabilitation across rehabilitation populations: A scoping review
Source: PLoS One. 2024 Nov 19;19(11):e0313440. doi: 10.1371/journal.pone.0313440 (PMC11575805; doi:10.1371/journal.pone.0313440)
Supplement: Attachment — Submitted filename: Response to Reviewers.docx [file pone.0313440.s009.docx]

September 12, 2024

Subject: Revision submission of our revised manuscript, “*Adverse events associated with the delivery of telerehabilitation across rehabilitation populations: A scoping review”* for consideration for publication in *PLOS ONE.*

Dear Reviewers:

Please find enclosed the revised manuscript, “*Adverse events associated with the delivery of telerehabilitation across rehabilitation populations: A scoping review”*  for consideration for publication as a scoping review in *PLOS ONE*. We would like to thank you for the opportunity to resubmit a revised copy of this manuscript. We would also like to take this opportunity to express our thanks to the reviewers for the positive feedback and helpful comments for correction or modification. The manuscript has been revised to address the reviewers’ comments which are marked using track changes. We also provided our response to their comments below. The revisions have been developed in consultation with all coauthors, and each author has given approval to the final form of this revision.

Responses to Reviewers’ Comments

**Reviewer 1**

**Comment 1:** Telerehabilitation can also provide services to those who would not normally be

able to access traditional rehabilitation, such as those living in remote communities or patients

with disabilities which hinder participation in in-person sessions. This is assuming that appropriate internet connections are available

**Response 1:** Thank you for the comment. The revised sentence now reads: *“Telerehabilitation can also provide services to those who would not normally be able to access traditional rehabilitation, such as those living in remote communities or patients with disabilities which hinder participation in in-person sessions [2], assuming that appropriate internet connections are available.”* *(page 5)*

**Comment 2:** Quality/risk of bias assessment was not completed as this is not the purpose of a scoping review. It should be “was”.

**Response 2:** Thank you for the comment. The revised sentence now reads: “*Quality/risk of bias assessment was not completed as this was not the purpose of a scoping review.” (page 9)*

**Comment 3:** The purpose of this scoping review was to map the existing research on adverse events during the delivery of telerehabilitation. Also, please briefly restate the problem you identified and the rational/need for this study.

**Response 3:**

Thank you for the suggestion. “The purpose of this scoping review was to map the existing research on adverse events during the delivery of telerehabilitation” has been rephrased to include the full aim statement and problem identified. *“There has yet to be any synthesis of the existing literature that summarizes the currently available research on adverse events related to telerehabilitation. This scoping review aimed to conduct a systematic search of published literature on adverse events during the delivery of telerehabilitation, across rehabilitation populations, and map out the extent of existing research. This included identifying characteristics of adverse events (frequency, physical versus non-physical, relatedness to telerehabilitation, severity) and examining adverse events for different modes of telerehabilitation delivery and disease states.” (page 17)*

**Comment 4:** In addition, most of the adverse events were defined as non-serious, mild, or minor. instead of stating "most" please provide an explicit percentage.

**Response 4:** Thank you for the comment. The sentence has been revised to be more explicit with a percentage. “*In addition, for the adverse events with severity information available, 87.4% were defined as non-serious, mild, or minor*.” *(page 20)*

**Comment 5:** When categorized by disease type, cardiac rehabilitation studies had the most frequent adverse events. Add “number of” adverse events.

**Response 5:** Thank you for the comment. The revised sentence now reads: “*When categorized by disease type, cardiac rehabilitation studies had the most frequent number of adverse events.” (page 24)*

**Reviewer 2**

**Comment 1:** Language problems should be corrected

**Response 1:** Thank you for the comment. The paper has been run through the Grammarly software and any identified language problems have been corrected.

**Comment 2:** The identified problem and rationale for conducted study should be presented explicitly

**Response 2:** Thank you for the comment. As per above, the discussion now starts with the full aim statement and problem identified. This is also reflected in the abstract and introduction sections. The rationale for the current review is as follows: *“There has yet to be any synthesis of the existing literature that summarizes the currently available research on adverse events related to telerehabilitation. Thus, this scoping review aimed to conduct a systematic search of published literature on adverse events during the delivery of telerehabilitation, across rehabilitation populations, and map out the extent of existing research. This included identifying characteristics of adverse events (frequency, physical versus non-physical, relatedness to telerehabilitation, severity) and examining adverse events for different modes of telerehabilitation delivery and disease states.” (page 17)*

**Comment 3:** not needed column in Appendix S3 table should be removed.

**Response 3:** Thank you for the comment. Appendix S3 table’s extra column has been removed, and remaining column edited to include reference number as per the following instructions: “For S3 Appendix, it is sufficient to list only the authors (e.g., 'Lundgren et al.') as in the second column. Just need to include the reference number that was cited in the text after the authors' name e.g., Lundgren et al. [ ] in the second column. First column is to be omitted.”

**Comment 4:** estimation and comparison of measurement errors should be presented or lack of it should be listed as limitation of the study

**Response 4:** Thank you for the suggestion. The limitations section has been revised to include this: No risk of bias assessment, *or estimation and comparison of measurement errors were* conducted, consistent with the PRISMA-ScR guidelines, as the aim of a scoping review is to map out the extent of literature rather than assess the quality of studies [17, 18].

We hope that we have satisfactorily addressed the reviewers’ comments. Please do not hesitate to contact us if you require any further information. Thank you and have a wonderful day.

Kind Regards,

**Sarah Munce, PhD** on behalf of the Team

Bloorview Research Institute, Holland Bloorview Kids Rehabilitation Hospital & Institute of Health Policy, Management and Evaluation, Rehabilitation Sciences Institute

E-mail: sarah.munce@uhn.ca
